# Carbonic anhydrase IX downregulation linked to disruption of HIF-1, NFκB and STAT3 pathways as a new mechanism of ibuprofen anti-cancer effect

**DOI:** 10.1371/journal.pone.0323635

**Published:** 2025-05-23

**Authors:** Katarina Grossmannova, Petra Belvoncikova, Barbora Puzderova, Veronika Simko, Lucia Csaderova, Jaromir Pastorek, Monika Barathova

**Affiliations:** 1 Institute of Virology, Biomedical Research Center, Slovak Academy of Sciences, Bratislava, Slovakia; 2 Mabpro, a.s., Bratislava, Slovakia; University of Navarra School of Medicine and Center for Applied Medical Research (CIMA), SPAIN

## Abstract

Numerous studies have highlighted the anti-cancer effects of nonsteroidal anti-inflammatory drugs (NSAIDs), although the underlying mechanisms remain unclear. This study focuses on elucidating the impact of the NSAID ibuprofen (IBU) on cancer cells exposed to hypoxia, as the hypoxic microenvironment significantly influences tumor progression, metastatic potential, and therapy resistance. Given that carbonic anhydrase IX (CA IX) is a key hypoxia-associated protein and a promising therapeutic target due to its tumor-specific expression, we primarily examined the impact of IBU on CA IX and the transcription factors regulating CA IX expression. We found that IBU downregulates expression and protein level of CA IX in hypoxic colon carcinoma and head and neck cancer cells, resulting in a reduction of membranous CA IX. To elucidate the mechanism of this phenomenon, we analyzed the key CA IX-regulating transcription factor HIF-1 and found decreased levels of the HIF-1α subunit in IBU-treated cells, leading to its impaired binding to the *CA9* promotor. Analysis of another transcription factor involved in CA IX expression, NFκB, showed suppressed NFκB pathway under IBU treatment. Moreover, we demonstrated IBU-mediated induction in apoptosis in cancer cells, as well as a decrease in their ability to migrate. Our study is the first to demonstrate that ibuprofen downregulates carbonic anhydrase IX expression in hypoxic colon and head and neck tumor cells by decreasing HIF-1α levels. Additionally, ibuprofen impairs key transcription factors NFκB and STAT3, leading to reduced adaptation to hypoxic stress, decreased tumor cell viability, and migration. This indicates its potential as a therapeutic agent in combination therapy for colon carcinoma or head and neck cancer.

## Introduction

The nonsteroidal anti-inflammatory drugs (NSAIDs) belong to the most commonly prescribed medications for the treatment of fever, pain and inflammation worldwide. Moreover, several epidemiologic studies have observed that the long-term and regular use of NSAIDs (ibuprofen, aspirin) is associated with reduced risk of various cancer types including prostate [1], colorectal [2], lung [3], breast [4], head and neck [5] or gastric cancers [6]. In general, NSAIDs block cyclooxygenase (COX)-catalyzed prostaglandin biosynthesis and thus inhibit cellular processes mediated by prostaglandin E_2_ (PGE_2_) and its receptors such as activation of signaling pathways involved in cell proliferation, migration, apoptosis, and/or angiogenesis [7]. There are two COX isoforms, COX-1 and COX-2, and based on COX selectivity, NSAIDs are classified as non-selective and COX-2 selective. Both COX-2 and prostaglandins also play a key role in the generation of the inflammatory response. PGE_2_ mediates augmentation of arterial dilatation, increases microvascular permeability resulting in edema and acts on the peripheral sensory neurons causing pain. It also contributes to the development of inflammatory granulation and regulate the function of many cell types including macrophages, T and B lymphocytes, and dendritic cells [8]. Numerous studies provide evidence that chronic inflammation increases risk of cancer, promotes tumor progression and metastasis [9]. Thus, the key anti-cancer mechanism of NSAIDs is believed to be caused by their anti-inflammatory properties, but it has also been demonstrated that NSAIDs induce cancer cell apoptosis and inhibit tumor angiogenesis [10]. However, the exact molecular mechanism of the anti-tumor action of NSAIDs remains unclear.

One of the key microenvironmental condition that occur in a majority of malignant tumors is hypoxia characterized by low oxygen concentration (between 1%-2% O_2_ and lower). Tumor cells in such microenvironment induce a number of signaling pathways allowing them to adapt to the unfavorable conditions. Most of the changes are activated by a crucial survival transcriptional program mediated by hypoxia-inducible factor (HIF) composed of HIF-1α and HIF-1β subunits. In the presence of oxygen, HIF-1α is hydroxylated by prolyl hydroxylase (PHD) and factor inhibiting HIF (FIH) enzymes, which facilitate HIF-1α binding to the von Hippel-Lindau (VHL) tumor suppressor protein leading to HIF-1α ubiquitination and degradation. In contrast, under hypoxia, the activity of PHD is inhibited, so HIF-1α accumulates, binds to HIF-1β and activates the transcription of more than 100 direct target genes [11,12]. To date, few studies analyzing the effect of NSAIDs on hypoxic-related processes (mainly angiogenesis) have shown that NSAIDs inhibit hypoxia-induced *in vitro* angiogenesis in gastric cells *via* increased expression of VHL leading to reduced accumulation of HIF-1α and its target gene vascular endothelial growth factor (VEGF) [13]. Similarly, NSAID-treatment of prostate cancer cells resulted in reduced levels of HIF-1α and HIF-2α and down-regulated VEGF and Glut-1 [14].

Critical for the survival of hypoxic tumor cells is the hypoxia-triggered shift toward glycolytic metabolism resulting in the development of an acidic extracellular microenvironment. Both hypoxia and acidosis are hallmarks of cancer associated with aggressiveness, metastasis, and resistance to chemotherapy and radiotherapy [15]. One of the most important hypoxia-induced proteins helping tumor cells adapt to the hostile acidic environment is carbonic anhydrase IX (CA IX). It is an enzyme facilitating bicarbonate production for the purpose of intracellular alkalinization. It was shown that CA IX contributes to many aspects of tumor progression such as altered expression of extracellular matrix components [16], reduced cell-cell adhesion, increased migration and invasion [17]. CA IX is present in a broad range of tumors and its expression correlates with resistance to conventional therapy and poor prognosis of cancer patients. Moreover, there is accumulating evidence of CA IX presence in non-cancerous diseases linked to hypoxia, for example abdominal aortic aneurysm [18] or cirrhosis [19]. All these facts indicate that CA IX is a promising biomarker and therapeutic target [20].

In this study, we evaluated the effect of a non-selective NSAID ibuprofen (IBU) on CA IX expression in cell lines derived from various cancer types cultivated as monolayers in hypoxic conditions or in 3D models of spheroids. Since CA IX expression is primarily dependent on the HIF transcription factor [21], we also analyzed changes in HIF-1α levels and its binding to the *CA9* promotor after IBU treatment. Moreover, we studied the effect of IBU on major pro-inflammatory transcription factors nuclear factor κB (NFκB) and signal transducers and activators of transcription 3 (STAT3), both of which are also involved in regulation of CA IX expression. Finally, we showed IBU-mediated impairment of cancer cell proliferation and migration.

## Results

### Ibuprofen downregulates carbonic anhydrase IX expression in colon cancer and head and neck cancer cell line monolayers at protein and mRNA level

**Fig 1:**
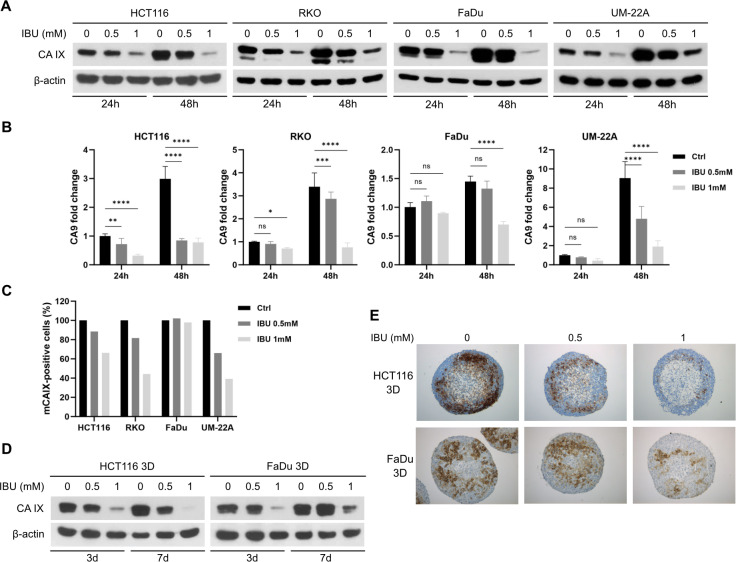
Effect of ibuprofen (IBU) on carbonic anhydrase IX (CA IX) expression. HCT116, RKO, FaDu and UM-22A cells cultured in monolayers in hypoxic conditions (1% O2) were lyzed at 24h and 48h of treatment with 0.5 mM IBU, 1 mM IBU, or DMSO as control (0 mM). (A) Detection of CA IX protein by Western blot showed that IBU downregulated CA IX protein levels in a time- and dose-dependent manner. (B) Detection of CA9 mRNA by q-PCR showed that IBU downregulated CA IX at mRNA level in a time- and dose-dependent manner. The results represent the mean from three independent biological experiments performed in triplicate. Control samples treated with DMSO for 24 hours were set to 1. Significance of differences was assessed by two-way ANOVA with Tukey´s multiple comparisons tests. P < 0.05 was considered significant. * denotes P < 0.05, **P < 0.01, *** P < 0.001, and **** P < 0.0001, respectively. (C) Detection of cells positive for CA IX PG-domain performed on live cells by flow cytometry showed, that IBU decreased the number of cells positive for membranous CA IX (mCA IX) in all four cell lines incubated in hypoxia under IBU treatment (0.5 mM and 1 mM) for 48 hours. Representative graph of the data from one of three independent experiments. For each of the samples, 10 000 cells were analyzed. Control cells were set to 100%. (D) 3D model spheroids formed from HCT116 and FaDu cells were incubated under IBU treatment (0.5 mM and 1 mM), or with DMSO as control. After 3 days and 7 days of treatment, spheroids were lyzed and CA IX was detected by Western blot. Results show that IBU downregulated CA IX protein in both types of spheroids. (E) HCT116 and FaDu spheroids treated with 0.5 mM and 1 mM IBU for 7 days were fixed in 4% paraformaldehyde followed by CA IX expression analysis on paraffin-embedded slides by immunohistochemistry. Results demonstrate that IBU reduced the level of CA IX protein in 3D models.

To evaluate the effect of IBU on CA IX expression in different cancer cell lines, we treated HCT116, RKO, FaDu, and UM-22A monolayers with 0.5 mM and 1 mM IBU for 24 h or 48 h in hypoxia (1% O_2_). DMSO treatment was used as negative control. After the required IBU exposure time, cells were lyzed and CA IX was detected by Western blot with the specific monoclonal antibody M75. As shown in Fig 1A, IBU suppressed CA IX expression in time- and dose- dependent manner in all treated cell lines. To analyze the effect of IBU on *CA9* mRNA levels, we isolated mRNA from treated monolayers and performed q-PCR. The results proved that IBU downregulated *CA9* mRNA levels similarly to reduction of CA IX protein (Fig 1B).

CA IX is a transmembrane enzyme with an active extracellular catalytic (CA) domain and proteoglycan (PG) domain. Since the specific monoclonal antibody M75 recognizes its extracellular PG domain, CA IX staining on live cells by flow cytometry allowed us to investigate the level of membranous CA IX (mCA IX). HCT116, RKO, FaDu and UM-22A monolayers were treated with 0.5 mM or 1 mM IBU or DMSO as a control. After 48 h treatment in hypoxia, live cells were incubated with M75 antibody and population of mCA IX-positive cells from a total of 10 000 single cells was detected by flow cytometry. Results in Fig 1C show that 0.5 mM IBU caused decrease in the amount of mCA IX level by 8% in HCT116 cells and by 13% in RKO cells and 1 mM IBU caused decrease of mCA IX level by 23% in HCT116 cells and by 33% in RKO cells. Interestingly, detection of mCA IX in head and neck cancer cell lines revealed that neither concentration of IBU affected level of mCA IX in FaDu cells. On the other hand, in UM-22A cells 0.5 mM IBU caused a 34% decrease and 1 mM IBU a 61% decrease of mCA IX-positive cells.

### Ibuprofen downregulates carbonic anhydrase IX expression in spheroids formed from colon cancer and head and neck cancer cells

Two-dimensional cell cultures have been widely used as a primary tool to investigate anticancer drug activity, although it is known that monolayers poorly mirror three-dimensional growth of tumors. One strategy to improve drugs transitioning into the clinic is the implementation of three-dimensional (3D) multicellular tumor spheroids that more accurately mimic human solid tumor architecture and biology such as cell-cell interactions, cell-extracellular matrix interactions and local gradients of nutrients, growth factors, secreted factors and oxygen. Additionally, cells in multi-layer tumor spheroids constitute a permeability barrier through which therapeutic agents must penetrate [22]. Spheroid cells form necrotic, hypoxic or quiescent, and proliferating zones, which differ in oxygen levels and thus naturally create conditions for hypoxia-induced CA IX expression.

To evaluate the effect of IBU on CA IX expression in 3D models, we analyzed the level of the CA IX protein in HCT116 and FaDu spheroids formed in hanging drops with subsequent treatment with 0.5 mM IBU, 1 mM IBU or DMSO (as a negative control) for 7 days. We then analyzed CA IX expression either in protein lysates by Western blot, or in paraformaldehyde-fixed paraffin-embedded slides by immunohistochemistry. As shown in Fig 1D, 1E, IBU suppressed CA IX expression in both types of cell spheroids in a dose-dependent manner similarly to the results obtained in monolayer treatment. Decreased CA IX level in IBU-treated spheroids was confirmed by both Western blot (Fig 1D) and immunohistochemistry (Fig 1E).

### Ibuprofen downregulates CA IX expression *via* HIF-1 pathway

Hypoxia-inducible factor 1 (HIF-1) is the key transcription factor regulating hypoxia-associated protein expression. The hypoxia response element (HRE) sequence, which is located immediately upstream of the transcription initiation site in the *CA9* promotor and is recognized by HIF-1, is the primary reason why CA IX is one of the best responders to hypoxia. [21].

**Fig 2:**
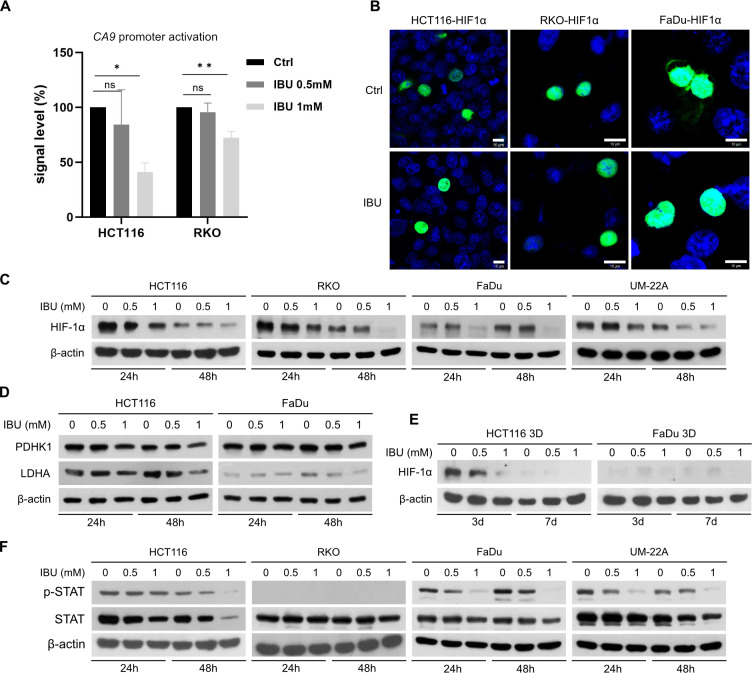
Effect of ibuprofen (IBU) on HIF-1α transcriptional activity and protein level. (A) Analysis of the HIF-1 transactivation potential in hypoxic HCT116 and RKO cells treated with IBU (0.5 mM and 1 mM). Cells were co-transfected with pGL3 luciferase vector with -174/ + 37 *CA9* genomic fragment containing HRE and pRL-TK plasmids. After 24 h incubation of transfected cells in normoxia, cells were passaged and subsequently treated with 0.5 mM IBU, 1 mM IBU, or DMSO. After incubation of treated cells for 24 h in hypoxia (1% O_2_), cells were harvested and analyzed by dual-luciferase assay. The graph shows that IBU administered during incubation of cells in hypoxia reduced the activity of the *CA9* promoter in a dose-dependent manner. Significance of differences between controls and IBU treated samples was assessed by one-way ANOVA with Dunnett’s multiple comparisons test, * denotes P < 0.05, ** P < 0.01. (B) Analysis of the ability of HIF-1α to translocate to the nucleus. HCT116, RKO and FaDu cells were transfected with pcDNA 3.1 plasmid expressing HIF-1α and treated with 0.8 mM IBU. After 48h incubation, cells were fixed and HIF-1α was detected by immunofluorescence staining using specific anti-HIF-1α primary antibody and secondary antibody with AlexaFluor488. Nuclei were stained with DAPI (blue). Samples were analyzed by Zeiss LSM 510 Meta confocal microscope, Plan Neofluar 40x/1.3 oil objective. Scale bar represents 10 μm. (C) Detection of HIF-1α protein by Western blot showed that IBU downregulated HIF-1α protein level in a time- and dose-dependent manner in all four analyzed cell lines incubated in monolayer in hypoxia. (D) Detection of HIF-1 target proteins PDHK1 and LDHA in HCT116 and FaDu cells treated with IBU in hypoxia revealed decreased levels of all proteins under 1 mM IBU treatment. (E) 3D model spheroids formed from HCT116 and FaDu cells were incubated under IBU treatment (0.5 mM and 1 mM), or with DMSO in cultured media as control. After 3 days and 7 days of treatment, spheroids were lyzed and HIF-1α was detected by Western blot. Results show that both IBU concentrations downregulated HIF-1α protein in HCT116 spheroids (HCT116 3D) and 1 mM IBU downregulated HIF-1α in FaDu spheroids (FaDu 3D). (F) Detection of phosphorylated STAT3 (p-STAT3) and total form of STAT3 in HCT116, RKO, FaDu and UM-22A cell monolayers incubated in hypoxia under IBU treatment (0.5 mM and 1 mM) by Western blot confirmed decreased levels of both proteins in HCT116, FaDu and UM-22A cells treated with IBU.

To evaluate the mechanism by which IBU downregulates CA IX expression, we analyzed the effect of IBU on HIF-1 ability to bind to the *CA9* promoter using pGL3 luciferase vector with a -174/ + 37 *CA9* genomic fragment containing HRE. Luciferase assay showed that IBU decreased the activity of the *CA9* promoter in HCT116 and RKO cells (Fig 2A). The nuclear translocation of HIF-1 is also required for successful transcriptional activation of hypoxia-regulated genes. To analyze whether IBU disrupts this process, we transiently transfected HIF-1α expressing vector to HCT116, RKO and FaDu cells. After 48 h of 0.8 mM IBU treatment (or DMSO treatment as control) of these cells, we visualized HIF-1α protein localization by immunofluorescence. Results showed nuclear HIF-1α staining in both DMSO and IBU-treated cell lines indicating that IBU has no effect on HIF-1α translocation to the nucleus (Fig 2B).

Next we examined the level of hypoxia-stabilized HIF-1α (oxygen-regulated subunit of HIF-1) and its downstream targets PHDK1 and LDHA in IBU-treated hypoxic cells by Western blot. Results proved, that both 0.5 mM and 1 mM IBU decreased the level of HIF-1α protein in HCT116, RKO, FaDu and UM-22A cells after 24 h or 48 h treatment in hypoxia (Fig 2C) and that both IBU concentrations caused decreased HIF-1α levels also in spheroids formed from HCT-116 cells (Fig 2E). Moreover, disruption of the HIF-1 pathway in cell monolayers cultured in hypoxia under 1 mM IBU was also demonstrated by decreased levels of other HIF-1 target proteins, namely PDHK1 and LDHA (Fig 2D).

Together these data suggest that the reduction of the CA IX protein by ibuprofen is mediated by disrupted HIF-1 binding to the *CA9* promoter region caused by decreased HIF-1α levels.

### Ibuprofen downregulates the level of STAT3 and its Y705 phosphorylated form (p-STAT3)

Signal transducer and activator of transcription 3 (STAT3) is commonly hyperactive in many cancers and is associated with cancer cell proliferation, invasion, migration, and angiogenesis. Thus, the STAT3 pathway is considered a promising target for cancer treatment. Moreover, recent studies have shown that STAT3 increases the binding kinetics of HIF-1 on the promoters of HIF-1 target genes, including *CA9* [23]. To determine the effect of IBU on STAT3 in hypoxia-incubated cells, we analyzed levels of total STAT3 and its phosphorylated form in IBU-treated hypoxic HCT116, RKO, FaDu and UM-22A cells. Our results determined that both 0.5 mM and 1 mM IBU concentrations reduced the levels of total STAT3 and its phosphorylated form p-STAT3 after 24 h or 48 h in hypoxia in HCT116, FaDu and UM-22A cells, which suggests an inhibitory effect of IBU on the STAT3 pathway (Fig 2F). Interestingly, we were not able to detect p-STAT3 in RKO cells.

### Ibuprofen disrupts NFκB p50 and p65 translocation to nucleus and inhibits activation of NFκB pathway

Studies analyzing the effects of NSAIDs on the NFκB transcription factor are not consistent, but most of them show NSAID-mediated degradation of the NFκB inhibitor IκBα, followed by translocation of NFκB p65 and p50 subunits to the nucleus, suggesting activation of the NFκB-dependent pathway [24–26]. On the other hand, other studies demonstrate downregulation of NFκB-dependent gene expression after NSAIDs treatment [27,28].

**Fig 3:**
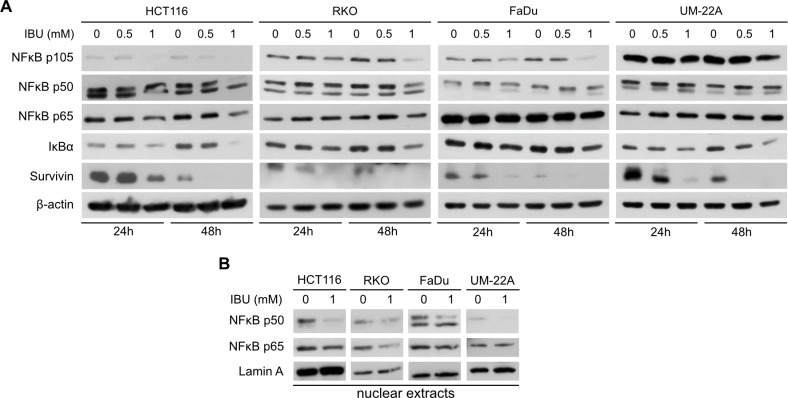
Effect of ibuprofen (IBU) on NF **κB protein level and its transcriptional activity.** (A) Detection of NFκB precursor p105, NFκB subunits p50 and p60 and other NFκB-related proteins in HCT116, RKO, FaDu and UM-22A cells incubated in hypoxia under IBU treatment (0.5 mM and 1 mM) by Western blot demonstrated decreased NFκB levels and impaired transcriptional activity of NFκB. (B) Detection of NFκB subunits p50 and p65 in nuclear extracts prepared from different cell lines treated with 1 mM IBU or DMSO as control for 48 hours in hypoxia showed decreased level of both subunits in all four treated cell line nuclei compared to levels in control nuclei from untreated cells.

Since NFκB belongs to transcription factors that bind to the *CA9* promotor and thus regulate *CA9* transcription [29], we examined the potential relationship between NFκB activity and IBU-mediated downregulation of CA IX expression. To test this hypothesis, we analyzed the levels of IkBα and NFκB p105, p50 and p65 proteins in HCT116, RKO, FaDu and UM-22A cells incubated in monolayers in hypoxia for 24 h or 48 h with or without IBU treatment (0.5 mM and 1 mM). Results shown in Fig 3A demonstrate that only higher concentration of IBU (1 mM) affects the investigated proteins. 1 mM IBU treatment downregulated NFκB precursor p105 in all treated cell lines, while p50 levels were reduced in colon carcinoma HCT116 and RKO cell lines and the level of the p65 protein was decreased only in HCT116 cells. Moreover, 1 mM IBU also caused a decrease of IκBαin all four cell lines, indicating release of NFκB p50/p65 from the IκBα/NFκB complex and its activation/translocation to the nucleus. To verify if NFκB translocate to the nucleus, we prepared nuclear fractions from HCT116, RKO, FaDu and UM-22A monolayers treated with 1 mM IBU for 48h in hypoxia and analyzed p50 and p65 levels in these fractions by Western blot. Results proved that IBU decreased the levels of both p50 and p65 in all four treated cell line nuclei compared to the levels in control nuclei from untreated cells (Fig 3B). To demonstrate IBU-mediated disruption of the NFκB pathway, we analyzed the level of the NFκB target protein survivin and found decreased survivin in all four cell line monolayers affected by IBU (Fig 3A). Together our data indicate, that IBU compromised the translocation of NFκB p50 and p65 to the nucleus and thus disrupted NFκB-mediated signaling despite downregulation of IκBα.

### Ibuprofen induces COX-2 expression via PI3K/AKT and ERK1/2 pathways in HCT116, RKO, FaDu and UM-22A cells

There are only few studies analyzing CA IX and COX-2 (encoded by the *PTGS2* gene) co-expression in tumors, but these studies suggest that COX-2/CA-IX protein expression levels correlate with each other and that this COX-2/CA IX axis promotes malignant behavior in colorectal cancer cells [30]. To explore a possible relationship between the expression of *PTGS2* and *CA9* in colon carcinoma and head and neck carcinoma, we used correlation analysis in GEPIA2 which showed no correlation between the expression of these two genes in COAD and HNSC datasets as the value of correlation coefficients was around 0 (0.062 and -0.018, respectively). We also performed correlation analysis based on TCGA data from colon and head and neck carcinoma provided in UCSC Xena which confirmed that *CA9* and *PTGS2* expression does not correlate (correlation coefficient -0.004 and 0.04 for COAD and HNSC, respectively).

**Fig 4:**
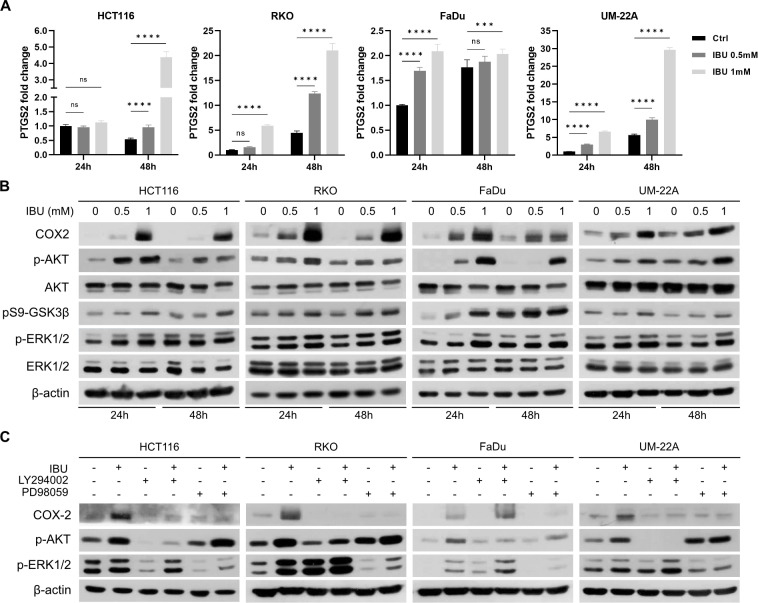
Effect of ibuprofen (IBU) on COX-2 expression. (A) Detection of COX-2 mRNA (*PTGS2*) by q-PCR showed that IBU upregulated COX-2 at mRNA level in a time- and dose-dependent manner in all cell lines treated with 0.5 mM or 1 mM IBU for 24 hours and 48 hours in hypoxia. The results represent the mean from three independent biological experiments performed in triplicate. Control samples treated with DMSO for 24 hours were set to 1. Significance of differences was assessed by two-way ANOVA with Tukey´s multiple comparisons tests. P < 0.05 was considered significant. *denotes P < 0.05, ** P < 0.01, *** P < 0.001, and **** P < 0.0001, respectively. (B) Analysis of COX-2 level and signal transduction pathways affecting COX-2 expression in cell monolayers incubated in hypoxia (1% O_2_) under 0.5 mM and 1 mM treatment for 24 hours and 48 hours. Results from Western blot show increased levels of COX-2, phosphorylated AKT (p-AKT), phosphorylated GSK3β at Serine 9 (pS9-GSK3β) and phosphorylated ERK1/2 (p-ERK1/2) in all four examined cell lines. (C) Analysis of signaling pathways involved in COX-2 expression using specific inhibitors LY294002 (inhibiting PI3K/AKT pathway) and PD98059 (inhibiting ERK1/2 pathway) in hypoxic cell lines show that both pathways are involved in COX-2 expression in HCT116, RKO and UM-22A cells. In FaDu cells, only the ERK1/2 inhibitor decreased the level of IBU-mediated COX-2 expression.

IBU’s mechanism of action is reversible inhibition of the cyclooxygenase enzymes COX-1 and COX-2. COX-1 is constitutively expressed in various tissues, whereas COX-2 is overexpressed in sites of inflammation and cancer including colorectal and head and neck cancer [31,32]. Thus we wanted to analyze, if IBU affects not only activity, but also expression of COX-2 in cancer cells. Interestingly, detection of COX-2 levels in all analyzed cell lines showed higher basal expression of COX-2 at mRNA and protein level in a dose- and time-dependent manner in IBU-treated cells compared to controls (Fig 4A, 4B).

One of the key transcription factor regulating COX-2 expression is NFκB, but since we found that NFκB activity is inhibited by IBU, we sought another mechanism to explain IBU-mediated COX-2 upregulation and analyzed signal transduction pathways that could lead to COX-2 expression. Recent studies showed that AKT-mediated inhibition of GSK3β leads to upregulation of COX-2 [28,33]. Thus we investigated levels of phosphorylated AKT (p-AKT) and GSK3β phosphorylated at inhibitory site Serine 9 (pS9-GSK3β) in hypoxic IBU-treated cells by Western blot. Examination of protein lysates from all four cell lines revealed a dose- and time-dependent increase in phosphorylated AKT and pS9-GSK3β (Fig 4B).

Another pathway involved in COX-2 expression is MAPK/ERK [34]. We discovered that IBU increased the level of phosphorylated ERK1/2 (p-ERK1/2) in all four hypoxia-incubated cell lines (Fig 4B).

Moreover, we proved that inhibition of both PI3K/AKT (using inhibitor LY294002) and ERK1/2 (using inhibitor PD98059) pathways suppressed IBU-mediated basal expression of COX-2 protein in hypoxic HCT116, RKO and UM-22A cells, indicating that both pathways are involved in COX-2 expression. On the other hand, in FaDu cells only the ERK1/2 inhibitor decreased the level of IBU-mediated COX-2 expression, indicating that in this cell line only the MAPK/ERK pathway induces COX-2 (Fig 4C).

### Ibuprofen induces apoptosis and autophagy of colon cancer and head and neck cancer cells cultured in monolayers and spheroids and inhibits cell migration

**Fig 5:**
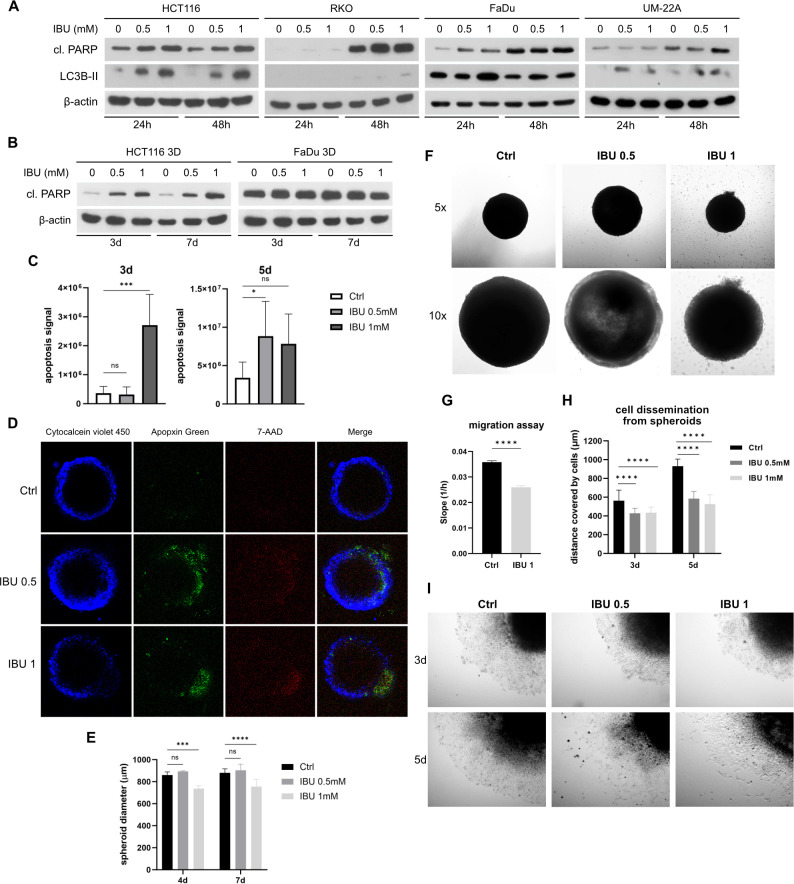
Effect of ibuprofen (IBU) on cancer cell apoptosis, autophagy and migration ability. (A) Western blot analysis of the level of the apoptosis marker cleaved form of poly (ADP‐ribose) polymerase (cl. PARP) and autophagy marker LC3B-II in protein lysates from HCT116, RKO, FaDu and UM-22A monolayers incubated in hypoxia for 24h or 48h with 0.5 mM or 1 mM IBU shows increased levels of both proteins in all four examined cell lines under IBU treatment. (B) Western blot analysis of cl. PARP in 3D models prepared from HCT116 and FaDu cells treated with 0.5 mM and 1 mM IBU show increased level of cl. PARP only in HCT116 spheroids under IBU treatment. (C) Determination of the level of apoptotic cells in HCT116 spheroids after 3 days and 5 days of incubation of spheroids in medium containing 0.5 mM or 1 mM IBU. Spheroids were stained with Apopxin Green and the signal was analyzed using ImageJ 1.41g. Results demonstrate that ibuprofen induced apoptosis in spheroid cells. Significance of differences in the levels of apoptosis between control and treated samples was assessed by one-way ANOVA with Dunnett’s multiple comparisons test, * denotes P < 0.05, ** P < 0.01, *** P < 0.001. (D) Immunofluorescence staining of HCT116 spheroids treated with 0.5 mM or 1 mM IBU for 5 days. Staining was performed using Apoptosis/Necrosis Kit containing Apopxin Green to visualize apoptotic cells, 7-AAD to visualize late apoptotic or necrotic cells and Cytocalcein violet 450 to visualize viable cells. (E) Graph representing diameters of HCT116 spheroids treated with 0.5 mM or 1 mM ibuprofen for 7 days using a Zeiss Axiovert 40 CFL microscope. Results showed that only 1 mM IBU significantly slowed spheroid growth. Significance of differences was assessed by two-way ANOVA with Tukey´s multiple comparisons tests. P < 0.05 was considered significant. *** P < 0.001, and **** P < 0.0001, respectively. (F) Representative images of HCT116 spheroids after 7 days of incubation with 0.5 mM and 1 mM IBU in cultured media. (G) Graph representing result from migration experiments performed on the xCELLigence RTCA DC device showing slowed migration of HCT116 cells under 1 mM IBU treatment. Significance of difference was assessed by t-test, **** denotes P < 0.0001. (H) Examination of cell dissemination from HCT116 spheroids. Graph represents mean values of distance covered by cells from spheroids incubated in cultured medium with 0.5 mM and 1 mM IBU for 5 days. Results show that cells were less capable of migration from spheroid when treated with IBU. (I) Representative images of cells migrating from spheroid under 0.5 mM and 1 mM IBU treatment for 5 days. Significance of differences was assessed by two-way ANOVA with Dunnett´s multiple comparisons tests. **** denotes P < 0.0001.

Since our previously described results showed that IBU decreases the levels of hypoxia-associated proteins HIF-1α and CA IX, which are crucial for cell adaptation to the hypoxic microenvironment, and we also demonstrated decreased levels of the anti-apoptotic protein survivin (Fig 3A), we performed analysis of apoptotic and autophagy markers in IBU-treated cells to see how disruption of cell adaptation affects cell survival in hypoxic condition. To investigate apoptotic status of IBU-treated cells we analyzed the level of cleaved form of poly (ADP‐ribose) polymerase (PARP) in protein lysates from HCT116, RKO, FaDu and UM-22A monolayers incubated in hypoxia for 24h or 48h with 0.5 mM or 1 mM IBU (Fig 5A) and the level of cleaved-PARP in protein lysates from HCT116 and FaDu spheroids treated with 0.5 mM or 1mM IBU on the 3rd and 7th day of treatment (Fig 5B). Moreover, we determined the level of LC3B-II (a standard marker for autophagosomes) in all four cell line monolayers. Western blot results demonstrated higher levels of both cleaved-PARP and LC3B-II in all four analyzed cell monolayers in response to ibuprofen treatment under hypoxic condition (Fig 5A), although in the 3D model the elevated cleaved-PARP was evidenced only in HCT116 spheroids (Fig 5B). These data suggest that ibuprofen induces apoptosis and affects autophagy in hypoxic cancer cells.

To determine the level of apoptotic, necrotic and viable cells in control and IBU-treated spheroids, we performed HCT116 spheroids staining with Apoptosis/Necrosis Kit containing Apopxin Green to visualize apoptotic cells, 7-AAD to visualize late apoptotic or necrotic cells and Cytocalcein violet 450 to visualize viable cells. Spheroids staining was performed on the 3rd and 5th day of 0.5 mM and 1 mM IBU treatment. Results showed that ibuprofen induced apoptosis in spheroid cells in a dose-dependent and time-dependent manner (Fig 5C, 5D). Moreover, in 1 mM IBU-treated spheroids (5th day) we also detected cells positive for 7-AAD. Localization of the 7-AAD signal indicates clusters of late apoptotic/necrotic cells released from the necrotic core (Fig 5D).

To investigate the effect of IBU on spheroid growth, we measured diameters of individually cultivated HCT116 spheroids treated with 0.5 mM or 1 mM ibuprofen for 7 days using a Zeiss Axiovert 40 CFL microscope. Results showed that only 1 mM IBU significantly slowed spheroid growth (Fig 5E), although examination of spheroids by light microscopy revealed less dense cores of 0.5 mM IBU-treated spheroids compared to controls (DMSO-treated spheroids), which could be due to wider necrotic zone within spheroids affected by ibuprofen. In addition, as mentioned above, spheroids treated with 1mM IBU released clusters of cells from the inside (Fig 5F). To evaluate the invasive capacity of these released cells (assuming that these cells are late-apoptotic or necrotic), we further cultivated the extruded cell clusters and found them unable to adhere and proliferate in monolayer.

Cancer cell migration is one of the key characteristics that facilitates the dissociation of cancer cells from the primary tumor. Therefore, the migration plays a crucial role in the metastatic cascade. To investigate the functional effect of IBU on cell migration, we used the xCELLigence RTCA instrument to examine the migration status of 1 mM IBU-treated HCT116 cells under hypoxia. Data demonstrated slower migration of hypoxic monolayer cells affected by ibuprofen compared to control cells (Fig 5G). To analyze the effect of ibuprofen on cell dissemination from spheroids, we used the Zeiss Axiovert 40 CFL microscope to observe the vicinity of adhered IBU- and DMSO-treated spheroids and to measure cell migration distance from the spheroid edge. Results presented in Fig 5H and 5I show that ibuprofen caused a 24% reduction in cell migration from spheroids on 3rd day of treatment and a 38% (0.5 mM IBU) or 44% (1 mM IBU) decrease in migration of spheroid cells on 5th day of treatment suggesting that ibuprofen may decrease migration capability of cells from primary tumor.

## Discussion

Cancer development is a complex process of selective and adaptive steps involving an extensive cast of molecules and signaling pathways. A solid tumor initially grows as an avascular bulk of cells with limited diffusion of nutrients, oxygen and metabolic waste products from the nearest functional blood vessels. These microenvironmental changes result in regional hypoxia and acidosis that put tumor cells under pressure to select a phenotype capable of activating angiogenesis, enhancing invasiveness and metastatic propensity, and gaining therapeutic resistance [35].

Carbonic anhydrase IX is one of the most important proteins promoting different phases of cancer development. Its expression is restricted to a few healthy tissues, but it is overexpressed in response to tumor hypoxia in many cancers. CA IX plays a critical role in hypoxia-associated tumor acidosis and development of the metastatic phenotype. Therefore, CA IX represents an attractive and promising target for systemic anticancer therapy [35]. Two major therapeutic tools are being studied for CA IX targeting: monoclonal antibodies and small molecule inhibitors (compounds based on sulfonamide/sulfamates and coumarins), but the aim of many studies is to find novel anti-CA IX therapeutics [36].

In our study we analyzed the possible effect of the non-steroidal anti-inflammatory drug ibuprofen on CA IX protein. NSAIDs belong to the most commonly prescribed medications used to relieve pain and to reduce inflammation. Moreover, it has been shown that the long-term and regular use of NSAIDs (ibuprofen, aspirin) is associated with a reduced risk of various cancer types [1–4,6,37]. Although the number of experimental studies investigating the effect of NSAIDs on cancer has increased recently, only few of them focused on monitoring the effects on cancer cells in hypoxic conditions. Thus, in the present study we analyzed the effect of IBU on hypoxic human cancer cell lines representing different types of cancers (colon carcinoma and head and neck cancer). The aim of our study was to find whether ibuprofen influences CA IX in these human cancer cells and if so, reveal the possible mechanism of this effect. The concentration of IBU in *in vitro* studies varies and reaches up to 2.5 mM. In the present study we treated four different carcinoma cell lines HCT116, RKO, FaDu and UM-22A with 0.5 mM or 1 mM ibuprofen, since it has been shown that the peak plasma drug concentration in humans can range from low levels up to 0.73 mM [38]. Hypoxic conditions of treated cells were set not only by incubating monolayers in the hypoxic chamber at 1% O_2_, but in our experiments we also used a 3D model (spheroids) that more accurately mimics the human solid tumor architecture and biology, such as cell-cell interactions, cell-extracellular matrix interactions and local gradients of nutrients, growth factors, secreted factors and oxygen and thus naturally creates hypoxic conditions for HIF-1α stabilization followed by CA IX expression [22]. Our results clearly show that ibuprofen decreased CA IX protein and mRNA levels in a dose- and time-dependent manner in all four examined cell line monolayers (Fig 1A, 1B) and also in spheroids formed from HCT116 and FaDu cells (Fig 1D, 1E). Since the extracellular domain of human CA IX is extended by a proteoglycan-like region, CA IX PG staining on live cells by flow cytometry allowed us to investigate the level of membranous CA IX (mCA IX). 0.5 mM IBU treatment in hypoxia resulted in a decrease in the amount of mCA IX by 8%, 13% and 34% in HCT116, RKO and UM-22A cells respectively, and 1 mM IBU decreased mCA IX by 23%, 33% and 61% in HCT116, RKO and UM-22A cells respectively. Similar results were described in an earlier study from our laboratory showing a decrease in mCA IX levels in cells treated with the chemotherapeutic drug doxorubicin [39]. Interestingly, detection of mCA IX in head and neck cancer cell line FaDu demonstrated that neither concentration of IBU affected the level of mCA IX, although Western blot analysis of whole cell lysate revealed a decrease in total CA IX in IBU-treated FaDu cells (Fig 1B). More experiments are needed to explain this phenomenon. Taken together, our results show that IBU affects CA IX expression in hypoxic cancer cells.

To elucidate the mechanism of CA IX downregulation, we analyzed HIF-1α expression in IBU-treated monolayers and spheroids. Since HIF-1 is the primary regulator of CA IX expression in contrast to many other hypoxia-inducible genes, due to hypoxia response element (HRE) localized immediately upstream of the transcription start site in the *CA9* promoter [21], using luciferase reporter assay we demonstrated that ibuprofen reduced *CA9* promoter activation (Fig 2A). Decreased HIF-1 activity under ibuprofen treatment was confirmed by the detection of decreased levels of HIF-1-responsive PDHK1 and LDHA (Fig 2D). We also showed that IBU caused a reduction in HIF-1α accumulation in IBU-treated hypoxic monolayers and spheroids by Western blot (Fig 2C, 2E). Our findings are consistent with a 2003 study that found that ibuprofen treatment reduced the levels of transcription factors HIF-1 and HIF-2 in hypoxic prostatic cancer cell lines PC3 and DU-145 [14].

Disruption of the HIF-1 pathway by ibuprofen may be the key mechanism of CA IX reduction in treated cells, but to elucidate the possibility of IBU-mediated influence on other important transcription factors/pathways, we analyzed the effect of IBU on STAT3 and nuclear factor kappa B (NFκB).

Signal transducer and activator of transcription protein 3 (STAT) affects various basic cell functions such as cell growth, survival, differentiation, regeneration, immune response, and cellular respiration. Constitutive activation of STAT3 plays an important role in tumor formation, development, metastasis, and recurrence. Therefore, the STAT3 pathway is a promising target for cancer therapy [40]. Moreover, it was shown that treating DU145 and A2058 tumor cells with a STAT3 inhibitor led to a reduction in HIF-1α [41]. STAT3 also increases the binding kinetics of HIF-1 on the promoters of HIF-1 target genes including *CA9* and thus STAT3 is involved in the HIF-1-mediated hypoxic transcriptional response [23,42]. Investigation of the STAT3 protein in our present study showed that ibuprofen caused a decrease in both the total STAT3 protein and its Y705 phosphorylated form p-STAT3 in HCT116, FaDu and UM-22A cell lines under hypoxic conditions in a dose- and time-dependent manner, indicating STAT3 pathway inhibition (Fig 2F). This is in line with studies demonstrating celecoxib-mediated inhibition of STAT3 phosphorylation in nasopharyngeal carcinoma cell lines [43] and ibuprofen- and diclofenac-mediated decrease in the level of phosphorylated STAT3 in glioma cell lines [44].

The NFκB transcription factors (complexes of homo- and heterodimers consisting of the subunits RelA/p65, c-Rel, RelB, p50 and p52) are key regulators of innate and adaptive immune responses. They control the expression of important regulatory genes involved in many critical physiological responses such as cell proliferation, differentiation, apoptosis, migration, invasion, angiogenesis and metastasis. Thus NFκB plays a critical pathological role in inflammatory disease including auto immunity and atherosclerosis, as well as in neurodegeneration and cancer. The canonical NFκB pathway is activated after degradation of the specific inhibitor of NFκB (IκBα) followed by the release of NFκB (predominantly the p50/p65 dimer) and its translocation to the nucleus, where it activates the expression of target genes [45,46].

Data from studies analyzing effect the of NSAIDs on the NFκB pathway are inconsistent. A study from 2004 showed that NSAIDs differ in their ability to suppress NFκB activation. Their results showed that none of the 11 investigated NSAIDs (including ibuprofen) by themselves activated basal NFκB. However, all NSAIDs inhibited TNF-induced NFκB activation and NFκB-regulated gene expression in a dose-dependent manner. The 50% inhibitory concentration for ibuprofen was 3.49 mM [47]. Studies of Stark’s group investigating the effect of aspirin on the NFκB pathway in colorectal cancer cells revealed aspirin-induced IκBα degradation followed by NFκB translocation to the nucleus [24]. Further experiments of this group brought evidences that aspirin and non-aspirin NSAIDs decrease the amount of IκBα followed by translocation of NFκB to the nucleus, indicating activation of the NFκB pathway, but the transcription of the NFκB target gene ICAM-1 was downregulated. They showed that the studied NSAIDs caused nucleolar compartmentalization of RelA/p65, which caused a reduction in the basal levels of NFκB transcriptional activity [27]. Similar results were demonstrated by a study from 2011, where it was shown that ibuprofen induced both IκBα degradation and nuclear localization of NFκB in human colon adenoma cells, however the activation of NFκB target genes (*Bcl-2*, *survivin*, *cyclin D1*) was suppressed [28]. Most of the studies investigating the effects of NSAIDs/ibuprofen on NFκB do not include cancer cells and hypoxic conditions. In our study, we examined the effect of IBU on NFκB p50 and p65 subunits in colon cancer cell lines (HCT116 and RKO) and head and neck cancer cell lines (FaDu and UM-22A) under hypoxia. We showed that 1mM IBU concentration caused a decrease in IκBα levels in all four analyzed cell lines suggesting release of NFκB from the IκBα/NFκB complex and activation of the NFκB pathway. On the other hand, 1 mM IBU also decreased precursor protein p105 in all four cell lines and its proteolytically cleaved p50 subunit in colon cell lines (Fig 3A). To elucidate NFκB activity, we investigated NFκB target protein survivin and found decreased levels of this protein in IBU treated cells (Fig 3A), although this downregulation could be also related to an impaired STAT3 pathway. Since nuclear localization of p50 and p65 is necessary for proper NFκB activity, we analyzed nuclear subunit levels and demonstrated impaired p50 and p65 translocation to the nucleus by detecting p50 and p65 in nuclear extracts prepared from untreated and IBU-treated cancer cells under hypoxia (Fig 3A). Importantly, there is accumulating evidence for a direct relationship between glycogen synthase kinase-3 beta (GSK3β) activity and the NFκB signaling pathway [48]. GSK3 is a serine/threonine kinase that was identified as a regulator of glycogen synthesis. Its activity is inhibited through phosphorylation of serine 21 in GSK3α and serine 9 in GSK3β. These serine residues of GSK3 have been previously identified as targets of AKT, a serine/threonine kinase located downstream of phosphatidylinositol 3-kinase (PI3K). It was shown that GSK3β inhibition disrupts NFκB-mediated gene transcription in pancreatic cancer [49]. In our experiments we found that IBU increased the phosphorylation of AKT, leading to increased inhibitory phosphorylation of GSK3β at Serine 9 (pS9-GSK3β) (Fig 4B). These results are in line with a study from 2011, where the authors demonstrate elevated levels of inhibitory phosphorylation of GSK3β under IBU treatment, although, in contrast to our results, they show increased translocation of NFκB to the nucleus [28]. Discrepancies in the results regarding NFκB translocation to the nucleus might be explained by the concentrations of IBU used in the studies. In a study from 2011, the authors treated cells with 2.5 mM IBU and in our study a maximum of 1 mM IBU was examined. Moreover, we investigated the effect of IBU on NFκB in hypoxic conditions, while only normoxic cells were analyzed in the other studies.

Studies analyzing the relationship between CA IX and COX-2 in colorectal cancer showed co-expression of these proteins and that this CA IX/COX-2 axis enhances malignant features of CRC cells [30]. Through *in silico* analysis, we were able to demonstrate that there was no relationship between the expression of the *CA9* and PTGS2 genes in colon carcinoma and head and neck cancer. IBU inhibits the production of prostaglandins by decreasing the activity of the enzymes COX-1 and COX-2, but we also wanted to analyze, if IBU affects COX-2 expression. Interestingly, we found that both IBU concentrations (0.5 mM and 1 mM) increased COX-2 mRNA and protein levels (Fig 4A, 4B). Only a few studies have analyzed the level of COX mRNA/protein in NSAID-treated cells. Similar data to ours have been described in a study of celecoxib in rat renal mesangial cells [25] and in Lewis Lung Carcinoma [50]. COX-2 expression was also induced by IBU in normal human bladder urothelial cell line UROtsa, but not in JIMT-1 (breast ductal adenocarcinoma) cells [51]. In a study of flufenamic acid, the authors conclude that it shows two opposing effects on COX-2 expression: it induces COX-2 expression in the colon cancer cell line HT-29 and macrophage cell line RAW 264.7, but it conversely inhibits tumor necrosis factor α (TNFα)- or lipopolysaccharide (LPS)-induced COX-2 expression. [52]. COX-2 expression is mediated by NFκB, but since our results demonstrated an impaired NFκB pathway, we sought an alternative mechanism to explain COX-2 upregulation under IBU treatment. It has been shown that several important pathways, for example PI3K/AKT and MAPK/ERK, are involved in regulation of COX-2 expression [53,54]. To elucidate the possible role of these pathways in IBU-mediated upregulation of COX-2 expression, we analyzed the levels of phosphorylated AKT and phosphorylated ERK1/2 in IBU-treated hypoxic cancer cell lines and confirmed that IBU activated both signaling pathways in a dose- and time- dependent manner (Fig 4B). Moreover, using the PI3K/AKT inhibitor LY294002 and ERK1/2 inhibitor PD98059 we proved that both inhibitors suppressed IBU-mediated basal expression of COX-2 protein in hypoxic HCT116, RKO and UM-22A cells indicating, that both pathways are involved in COX-2 expression. On the other hand, in FaDu cells only ERK1/2 inhibitor decreased the level of IBU-mediated COX-2 expression, which suggest that in this cell line only the MAPK/ERK pathway induces COX-2 (Fig 4C). A study from 2006 showed that inhibition of GSK3β by SB415286 induced the expression of COX-2 mRNA and protein, as well as its activity in gastric cancer cells. In contrast to inhibition of the PI3K/AKT pathway, inhibitors of ERK1/2 did not alter COX-2 expression in these cells [33]. Considering our results that show increased inhibitory phosphorylation of GSK3β (pSer9-GSK3β) under IBU treatment (Fig 4B), we suggest that IBU-mediated inhibition of GSK3β influences COX-2 expression in hypoxic colon cancer and head and neck cancer cell lines.

Since our findings revealed IBU-mediated inhibition of HIF-1α, NFκB and STAT3 transcription factors which are important for cell survival, we further investigated cell autophagy and apoptosis under ibuprofen treatment. Similarly to previous studies on the effect of NSAIDs on cell proliferation, we demonstrated that ibuprofen increased the levels of the apoptotic marker cleaved-PARP and the marker of autophagosomes LC3B-II in a dose- and time-dependent manner in hypoxic cell monolayers (Fig 5A), and decreased the level of the anti-apoptotic marker survivin (Fig 3A). Moreover, a higher number of apoptotic cells was also confirmed in the 3D model. HCT116 spheroids cultured in the presence of 1 mM ibuprofen for 3 days contained more apoptotic cells than controls. Spheroids cultured in the presence of both 0.5 mM and 1 mM ibuprofen for 5 days had impaired integrity and clusters of late apoptotic/necrotic cells released from the inside (Fig 5D). These results are similar to our results obtained in an earlier study where we observed such clusters after beta-adrenergic receptor blockade with propranolol [55]. Our findings indicate that ibuprofen-mediated changes in signaling pathways attenuated the ability of cells to adapt to hypoxic conditions and thus promote apoptosis in those cells. This phenomenon could be also related to the reduction of CA IX on the cell surface, since it has been shown, that the level of membranous CA IX decreases during the cell death progress due to increased CA IX ectodomain shedding mediated by disintegrin and metalloproteinase ADAM17 [39]. Even though we realize that we did not monitor autophagic flux, the increased level of LC3B-II in IBU-treated cells indicate changes in autophagosome formation.

Migration of cells is one of the crucial factors promoting metastasis. A study by Ferreira et al. (2021) showed that ibuprofen inhibited glioblastoma cell migration [56] and similarly, a study by Wynne and Djakiew demonstrated that ibuprofen suppressed cell migration of prostatic cancer PC-3 cells [57]. In the present study we investigated migration of HCT116 cells cultured in monolayer under hypoxic conditions. Our data revealed that ibuprofen slowed the migration of treated cells in comparison to controls (Fig 5G). Next, we examined the migration of cells from HCT116 spheroids. Our results show that the cells were less capable of migrating from the spheroid when treated with ibuprofen, which also indicates that ibuprofen may decrease the ability of cells to migrate from the primary tumor (Fig 5H).

**Fig 6:**
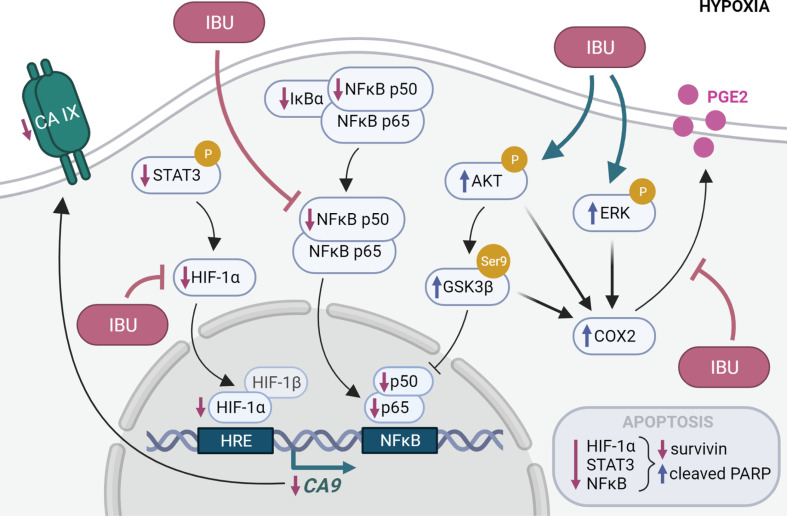
Proposed mechanism of action of ibuprofen (IBU) on hypoxic cancer cells. IBU impairs the STAT3 pathway and downregulates HIF-1α protein leading to impaired HIF-1 binding on the *CA9* promoter. The disruption of CA IX transcription results in a decreased level of CA IX on cancer cell membrane. Additionally, IBU impairs NFκB transcriptional activity by reducing the translocation of p50 and p65 to the nucleus despite decreased IκBα level. NFκB-mediated gene transcription is also hindered via inhibition of GSK3β. This involvement in crucial pathways results in apoptosis and autophagy of cancer cells. Our study also proved that despite the fact that IBU inhibits COX-2 activity, it paradoxically upregulates COX-2 expression *via* the PI3K/AKT and ERK1/2 pathways. Created with BioRender.com.

To our knowledge, our study is the first to show that ibuprofen downregulates carbonic anhydrase IX expression in hypoxic colon and head and neck tumor cells and decreases the level of membranous CA IX on cancer cell surface. Moreover, we proved that this downregulation is mediated by decrease in HIF-1α levels, resulting in impaired HIF-1 binding to the *CA9* promoter. We also demonstrated that ibuprofen impairs other important transcription factors, NFκB and STAT3. This involvement in key hypoxia-related signaling pathways and proteins results in reduced adaptation to hypoxic stress, followed by reduced tumor cell viability and migration (Fig 6). Taken together, our data revealed novel mechanisms through which NSAIDs may exert their anti-cancer effects and thus could be considered as therapeutics in colon or head and neck cancer in combination therapy.

## Conclusions

In conclusion, our study represents a precise investigation into the impact of NSAID ibuprofen on hypoxic colon and head and neck tumor cells. We have demonstrated for the first time, that ibuprofen significantly downregulates the expression of CA IX in these cells. Moreover, we revealed the mechanism of this phenomenon, which involves functional impairment of the key CA IX-regulating transcription factor HIF-1 and also inhibition of NFκB, an additional CA IX-regulating protein. Analysis of IBU-treated hypoxic cancer cell viability revealed increased apoptosis and affected autophagy, resulting in a decreased ability of cells to migrate.

This study not only enhances our understanding of the molecular mechanisms involved in NSAID action but also opens new strategies for the development of effective anti-cancer strategies. On the other hand, our results demonstrating a reduction of CA IX on hypoxic cell surfaces indicate a lower concentration of the target for CA IX-directed immunotherapy. Most importantly, our study brings new insight into a clinically relevant scenario that may arise while administering IBU treatment to patients with solid tumors.

## Materials and methods

### Monolayer cell culture and treatment

Colon carcinoma cells HCT116 (kind gift from RNDr. Borivoj Vojtesek, DrSc. from Masaryk Memorial Cancer Institute) and RKO and head and neck cancer cells UM-SCC-22A (designated UM-22A) (kind gifts from Nicolas C. Denko, MD, PhD from The Ohio State University) and FaDu (ATCC), were routinely cultured in Dulbecco´s modified Eagle´s medium (DMEM) supplemented with 10% fetal calf serum (HyClone Laboratories) and gentamicine (Sandoz) in humidified air (21% O_2_, 5% CO_2_) at 37°C. For hypoxia experiments (1% O_2_, 2% H_2_, 5% CO_2_, 91% N_2_), cells were cultured in InVivo2 anaerobic work station (Ruskinn Technology). Cells were treated with ibuprofen (IBU, dissolved in DMSO) added to culture medium at the final concentration 0.5 mM and 1 mM. Control cells were cultivated in the presence of DMSO alone. To inhibit PI3K/AKT or MAPK/ERK signaling pathway, cells were incubated with LY294002 (20 μM, Sigma), or PD98059 (20 μM, Sigma) respectively for 48 h in hypoxia in the absence/presence of 0.8 mM IBU.

### Formation of spheroids in hanging drops and treatment

The HCT116 and FaDu spheroids were formed in 20 μl hanging drops containing 100 cells/μl (HCT-116, FaDu) or 350 cells/μl (UM-22A) in Dulbecco´s modified Eagle´s medium supplemented with 10% fetal calf serum and gentamicine for 4 days of incubation in a humidified atmosphere on the lid of φ100 mm Petri dish. PBS was added into the dish to prevent drying of the drops. Resulting multicellular spheroids were carefully transferred to plates with a non‐adherent surface and cultivated in suspension in DMEM with IBU or DMSO (as control) for 7 more days. The spheroids were examined with a Zeiss Axiovert 40 CFL microscope, A‐Plan 5 × /0.12 objective (Zeiss) and processed by Axiovision 4.8 software. To examine the distance covered by cells migrating from spheroids, spheroids incubated in culture medium containing 0.5 mM, 1 mM IBU, or DMSO as control were allowed to attach to Petri dish surface (designated as time 0 min) and photographed in the transmitted light on the 3rd and 5th day. The migration of cells from a spheroid was measured as the distance which the cells reached from the edge of the spheroid.

### Western blot

After treatment with IBU or DMSO (as control), cells grown in monolayers or spheroids were lyzed in ice-cold lysis buffer [50 mM TrisHCl pH 7.4; 150 mM NaCl; 1% Triton X100; 0.05% NaDOC; 1 mM EDTA; 0.1% SDS; Protease Inhibitor Cocktail Tablets (Roche); Phosphatase Inhibitor Cocktail (Sigma)]. To separate nuclear extracts, lysates were prepared according to the REAP method [58]. Protein concentration was determined using the bicinochoninic acid kit (Thermo Scientific). Total protein extracts (30 μg/lane) were mixed with Laemmli buffer, separated in 10% SDS-PAGE and transferred onto polyvinylidene difluoride membrane (Immobilon TM-P, Millipore). After 30 min blocking with 5% non-fat dry milk in 0.1% Tween in PBS, membranes were incubated O/N at 4°C with primary antibodies: hybridoma medium containing mouse monoclonal antibody M75 recognizing the PG-domain of CA IX [59] (prepared at the Institute of Virology, Biomedical Research Center, 1:200 in blocking buffer); anti-β-actin (Cell Signaling, 1:8000); anti-HIF-1α (BD Transduction Laboratories, 1:250); anti-PDHK1 (Abcam, 1:1000); anti-LDHA (Cell Signaling, 1:1000); anti-phospho STAT3 (Cell Signaling, 1:500); anti-STAT3 (Cell Signaling, 1:1000); anti-NFκB p105/p50 (Cell Signaling, 1:1000); anti-NFκB p65 (Santa Cruz, 1:700); anti-IκBα (Cell Signaling, 1:1000); anti-survivin (Cell Signaling, 1:1000); anti-Lamin A (Santa Cruz, 1:700); anti-cleaved PARP (Cell Signaling, 1:1000); anti-COX-2 (Cell Signaling, 1:1000); anti-p-AKT (Ser 473) (Cell Signaling, 1:1000); anti-AKT (Cell Signaling, 1:1000); anti-p-ERK1/2 (Cell Signaling, 1:1000); anti-ERK1/2 (Cell Signaling, 1:1000); anti-pS9-GSK3β (Cell Signaling, 1:1000); anti-LC3B (Cell Signaling, 1:1000). After washing, membranes were incubated with HRP-conjugated anti-mouse, or anti-rabbit antibody (Dako, 1:5000 in blocking buffer, 1h, RT). Protein signals were visualized using enhanced chemiluminescence. Western blot analyses were repeated three times.

### Immunohistochemistry

The IBU- and DMSO-treated spheroids (7d treatment) were fixed in 4% Paraformaldehyde and embedded in paraffin. Antigen retrieval was performed in low pH retrieval buffer (EnVision® Flex Target Retrieval Solution Low pH) on PT‐Link (Dako). Immunohistochemical analysis was performed using Dako EnVision®FLEX + detection system (Dako) according to the manufacturer’s instructions. For CA IX staining, the sections were labelled with M75 antibody (hybridoma medium) diluted 1:100 for 1 h at room temperature and after washing incubated with secondary anti mouse‐HRP for 30 min at room temperature. Negative controls were prepared by omission of the primary antibody. The sections were counterstained with Mayer’s haematoxylin and mounted in Aquamount (Merck, Darmstadt, Germany). The stained sections were examined using a Leica DM4500B microscope and images were captured by a Leica DFC480 camera.

### RNA isolation and q-PCR

Total RNA from cell monolayers and spheroids was isolated using TRI Reagent solution followed by reverse transcription of 2 μg RNA with the High-Capacity cDNA Reverse Transription kit (Applied Biosystems) according to manufacturer´s instructions. Quantitative real-time PCR was performed on a StepOne Real-Time PCR System (Applied Biosystems) using POWER SYBR Green PCR Master Mix (Applied Biosystems) and the following primers: β-actin sense: 5´-CCAACCGCGAGAAGATGACC-3´; β-actin antisense: 5´-GATCTTCATGAGGTAGTCAGT-3´; *CA9* sense: 5´-TATCTGCACTCCTGCCCTCTG-3´; *CA9* antisense: 5´-CACAGGGTGTCAGAGAGGGTG-3´; *PTGS2* sense: 5´-TCC CTT GGG TGT CAA AGG TAA A-3´; *PTGS2* antisense: 5´-TGG CCC TCG CTT ATG ATC TG-3´.

### Flow cytometry

After 48h incubation of cells in hypoxia with 0.5 mM or 1 mM IBU added to medium, the cells were scraped from the Petri dishes into culture medium and centrifuged at 300g. Then the pellet was resuspended in culture medium with M75 monoclonal antibody and incubated for 10 min at RT. After washing with versene, cells were incubated with the secondary anti-mouse Alexa Fluor 488 antibody in culture medium for 10 min at RT. After washing, analysis of cell surface CA IX expression was performed on a Guava easyCyte Plus (Millipore) flow cytometer. Data were analyzed with CytoSoft 5.2 software (Millipore). Debris, cell doublets and clumps were excluded from analyses by scatter gating and a total of 10 000 single cells were analyzed for each sample.

### Luciferase reporter assay

HCT116 and RKO cells were seeded in 3.5 cm Petri dishes (5 x 10^5^ cells/dish). Following overnight incubation, cells were transfected with 2 μg of the pGL3 luciferase vector containing *CA9* promoter (-174/ + 37) and 100 ng pRL-TK Renilla vector using Tubofect transfection reagent (ThermoFisher). Human *CA9* promoter construct was generated by an insertion of PCR-amplified -174/ + 37 *CA9* genomic fragments upstream of the firefly luciferase gene in pGL3-Basic vector (Promega) [60]. After 24 h incubation of transfected cells in normoxia, cells were passaged onto 48-well plate and left for another 24 hours in normoxia. Subsequently, 0.5 mM IBU, 1 mM IBU, or DMSO were added to cultivation medium and the cells were transferred into hypoxia. After incubation for 24 h in hypoxia, cells were harvested and a luciferase assay was performed following the manufacturer’s instructions (Promega).

### Transient transfection of HIF-1α and immunofluorescence of HIF-1α

HCT116, RKO and FaDu cells were transiently transfected with TurboFect (ThermoFisher) according to the manufacturer´s instructions with pcDNA 3.1 vector containing HIF-1α and designated as HCT116-HIF1α, RKO-HIF1α and FaDu-HIF1α. Immediately after transfection, 0.8 mM ibuprofen or DMSO (as control) was added to culture medium and cells were incubated for 48 h. Then the cells were fixed with ice-cold methanol, blocked with 2% BSA in PBS and HIF-1α was detected using anti-HIF-1α antibody (BD Transduction Laboratories, 1:40 in 1% BSA in PBS, O/N, 4°C) followed by secondary anti-mouse/AlexaFluor 488 antibody. After washing, cover slips were mounted on glass slides with Mounting medium containing DAPI to visualize nuclei (Abcam). Samples were analyzed by Zeiss LSM 510 Meta confocal microscope, Plan Neofluar 40x/1.3 oil objective.

### *In silico* analysis

Correlation analysis of *PTGS2* and *CA9* in colon carcinoma and head and neck carcinoma was analyzed in GEPIA2 (Gene Expression Profiling Interactive Analysis tool) using COAD (colon adenocarcinoma) and HNSC (head-neck squamous cell carcinoma) datasets and in UCSC Xena (online exploration tool for public and private, multi-omic and clinical/phenotype data).

### Detection of apoptotic, necrotic and live cells

Apoptotic, necrotic and live cells in spheroids treated with 0.5 mM, 1 mM IBU or DMSO as control were stained with dyes from Apoptotis/Necrosis Assay Kit (blue, green, red) (Abcam) according to manufacturer´s instructions. The spheroids were analyzed using confocal microscopy LSM510 Meta (Zeiss), 20 × objective.

### Real-time monitoring of migration with xCELLigence system

Migration experiments were performed using the xCELLigence device (Roche) measuring electrical impedance expressed as cell index, reflecting the number of migrating cells. Cells were trypsinized, resuspended at the density of 4x10^5^ cell/ml in serum-free medium, added to the top chamber of the CIM-Plate 16 and allowed to migrate towards bottom chamber containing medium with 10% FCS as a chemoattractant. The CIM-Plate 16 was placed in the RTCA DP station and migration was monitored every 15 min for 100 h.

### Statistical analysis

Where applicable results were analyzed by two‐tailed unpaired t‐test, one-way or two-way analysis of variance (ANOVA) with appropriate multiple comparison test, and P < 0.05 was considered significant. P < 0.05 is denoted as *, P < 0.01 as **, P < 0.001 as ***, and P < 0.0001 as ****. Statistical analysis was performed in GraphPad Prism 9.5.1. If not stated otherwise the results in the graphs are expressed as means±standard deviation.

## Supporting information

S1 File Original Western blot images. (PDF)

## Abbreviations

AKT: protein kinase B
CA IX: carbonic anhydrase IX
COAD: colon adenocarcinoma
COX: cyclooxygenase
ERK: extracellular signal-regulated kinase
FIH: factor inhibiting hypoxia-inducible factor
GSK3β: glycogen synthase kinase 3 beta
HIF-1: hypoxia-inducible factor 1
HNSC: head-neck squamous cell carcinoma
HRE: hypoxia response element
ICAM-1: intercellular adhesion molecule 1
IBU: ibuprofen
IκBα: inhibitory subunit of NF kappa B alpha
LC3B: microtubule-associated protein-1 light chain 3
LDHA: lactate dehydrogenase A
MAPK: mitogen-activated protein kinase
mCA IX: membranous carbonic anhydrase IX
NFκB: nuclear factor kappa-light-chain-enhancer of activated B cells
NSAID: non-steroidal anti-inflammatory drugs
PARP: poly (ADP-ribose) polymerase
PDHK1: pyruvate dehydrogenase kinase 1
PGE2: prostaglandin E2
PHD: prolyl hydroxylase
PI3K: phosphatidylinositol 3-kinase
STAT3: signal transducer and activator of transcription 3
TNF: tumor necrosis factor
VEGF: vascular endothelial growth factor
VHL: von Hippel-Lindau
