# Supplementary material for: Carbonic anhydrase IX downregulation linked to disruption of HIF-1, NFκB and STAT3 pathways as a new mechanism of ibuprofen anti-cancer effect
Source: PLoS One. 2025 May 23;20(5):e0323635. doi: 10.1371/journal.pone.0323635 (PMC12101644; doi:10.1371/journal.pone.0323635)

HCT116 24 HY – CA IX (Figure 1A), HIF-1alpha (Figure 2C), cl. PARP (Figure 5A)

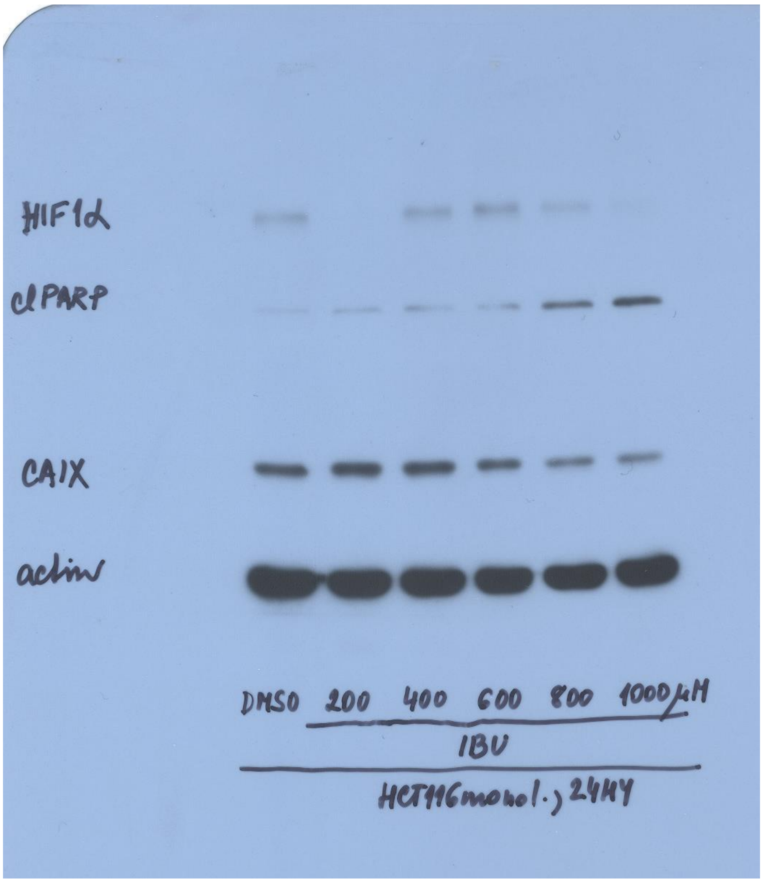

HCT 116 24 HY – CA IX (Figure 1A), p-AKT (Figure 4B)

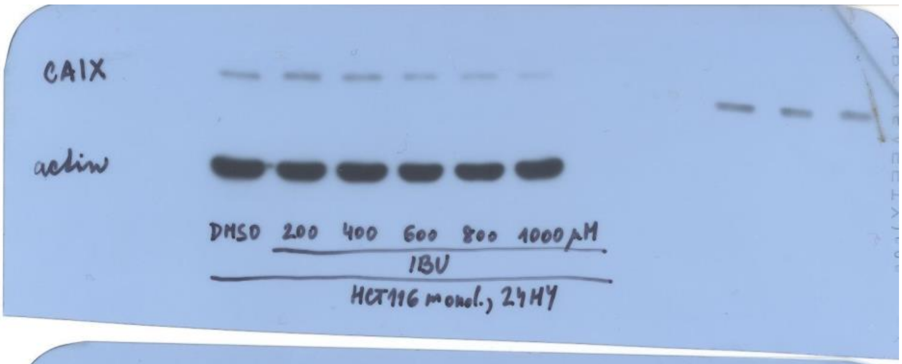

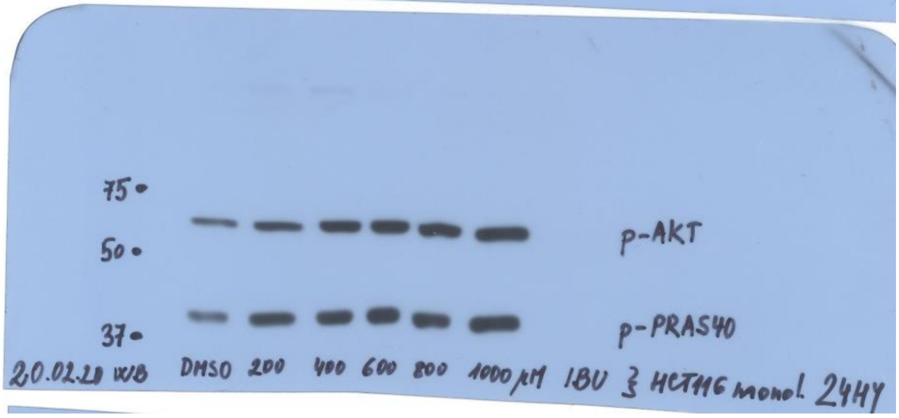

HCT 116 3D – CA IX (Figure 1D), cl- PARP (Figure 5B), aktín = actin

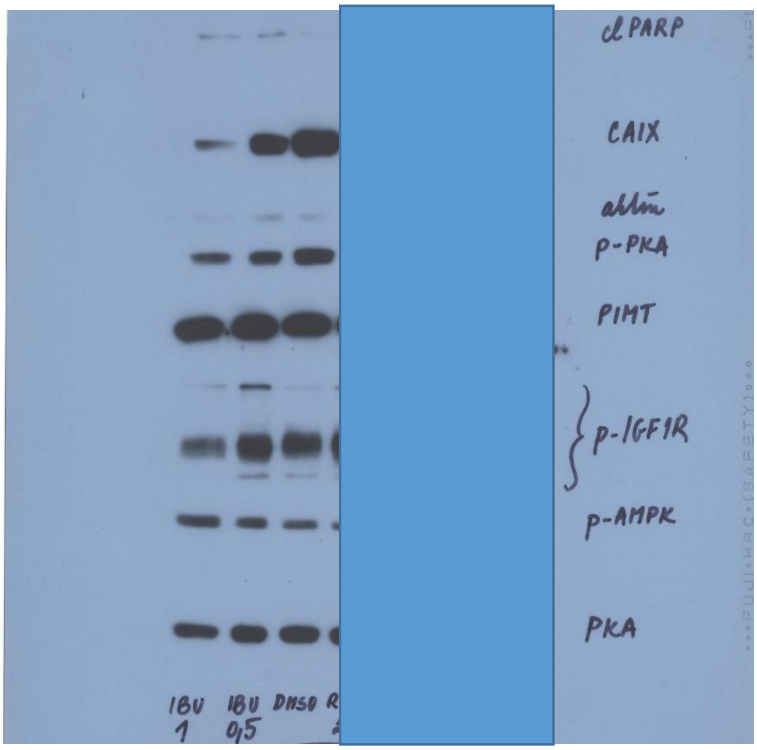

HCT 116 24HY, RKO 24 HY – CA IX (Figure 1A), aktín = actin

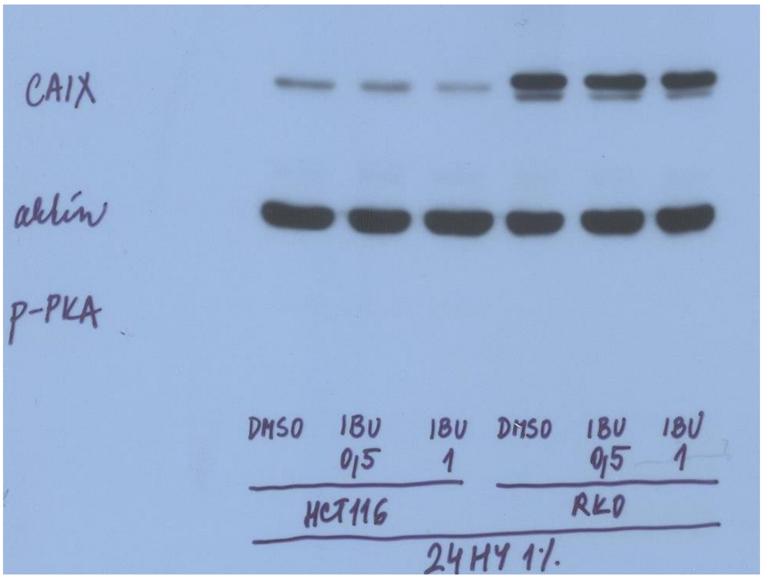

FaDu, UM-22A – CA IX (Figure 1A), HIF-1alpha (Figure 2C), cl. PARP (Figure 5A), SUR (Figure 3A), aktín = actin

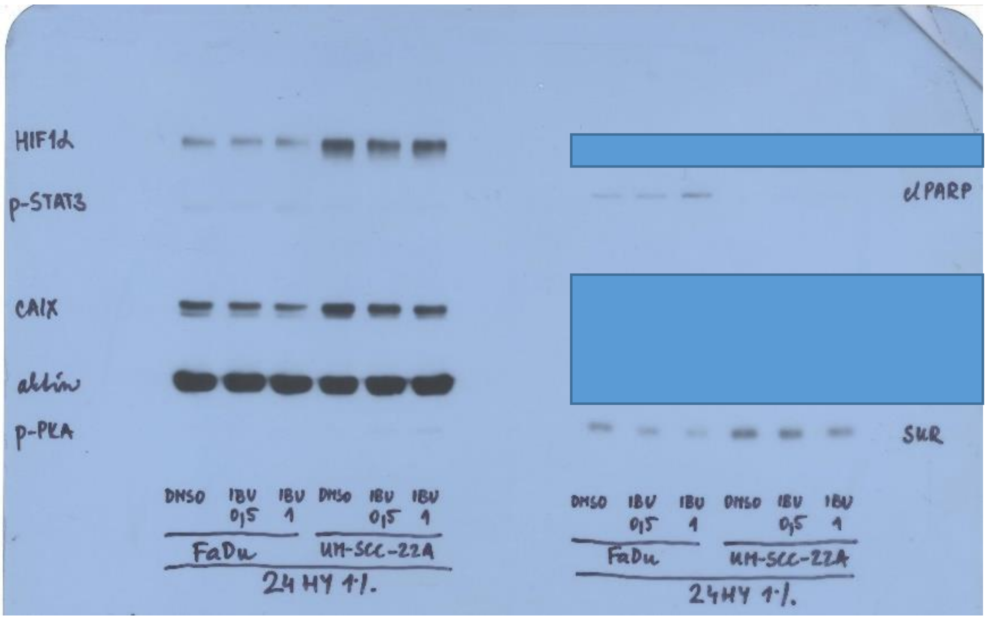

HCT116 24HY, 48HY – HIF-1alpha (Figure 2C – USED IN FIGURE), CA IX (Figure 1A – USED IN FIGURE), cl.PARP (Figure 5A – USED IN FIGURE), actin (USED IN Figure 1A, 2C, 5A)

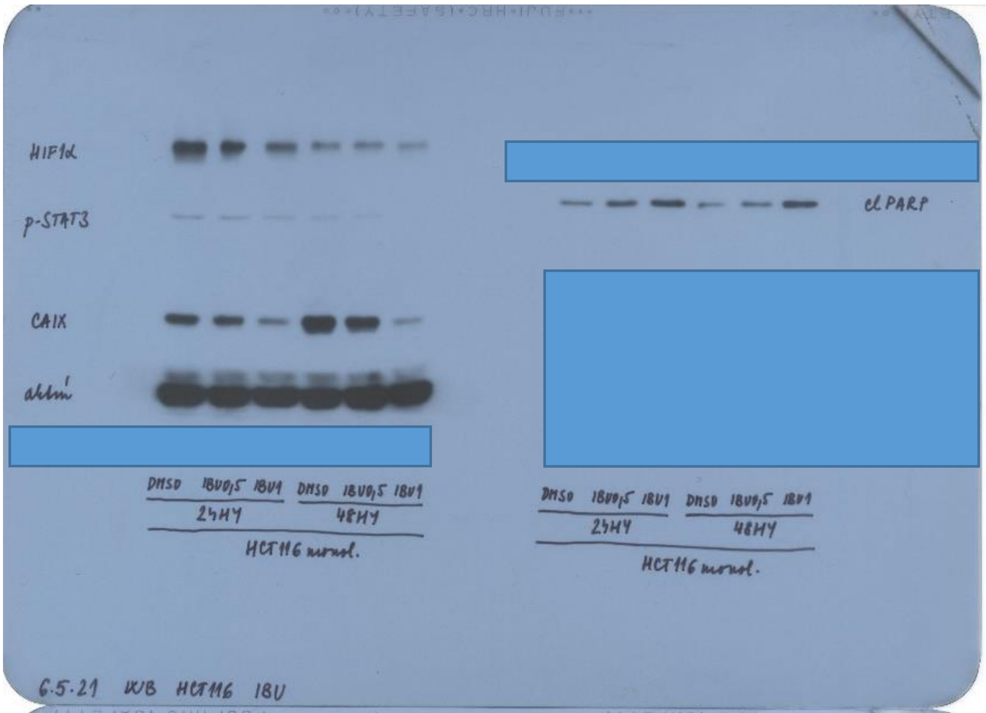

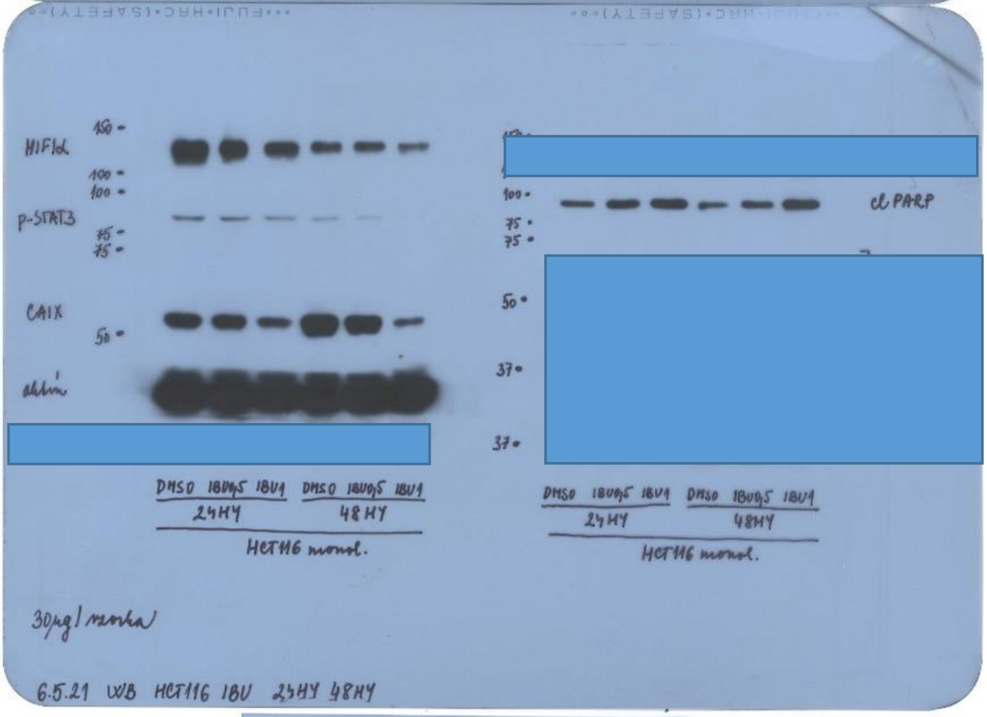

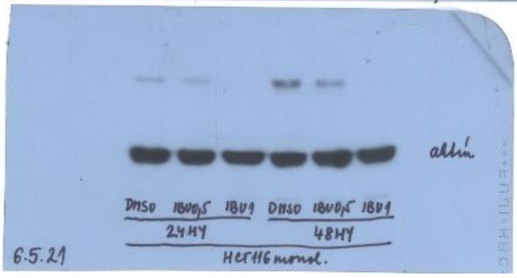

HCT116 24HY, 48HY – LC3B (Figure 5A – USED IN FIGURE), SUR = Survivin (Figure 3A – USED IN FIGURE), actin is above

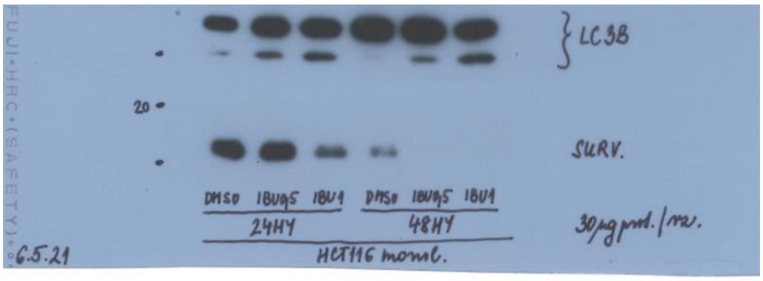

RKO 24HY, 48HY – HIF-1alpha (Figure 3C), p-STAT3 (Figure 2F), CA IX (Figure 1A), cl.PARP (Figure 5A), aktín = actin (USED IN FIGURE 5A)

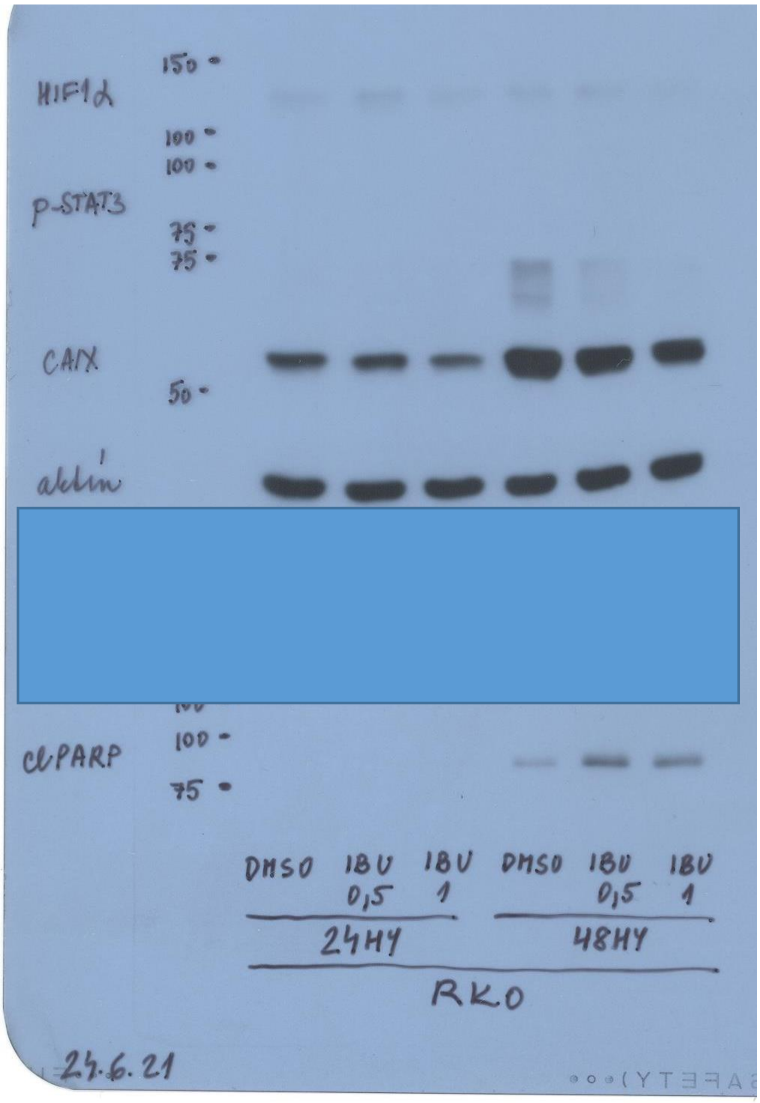

RKO 24HY, 48HY – HIF-1alpha (Figure 3C), p-STAT3 (Figure 2A), CA IX (Figure 1A), LC3B (Figure 5A – USED IN FIGURE), cl. PARP (USED IN FIGURE 5A), SURV = Survivin (Figure 3A)

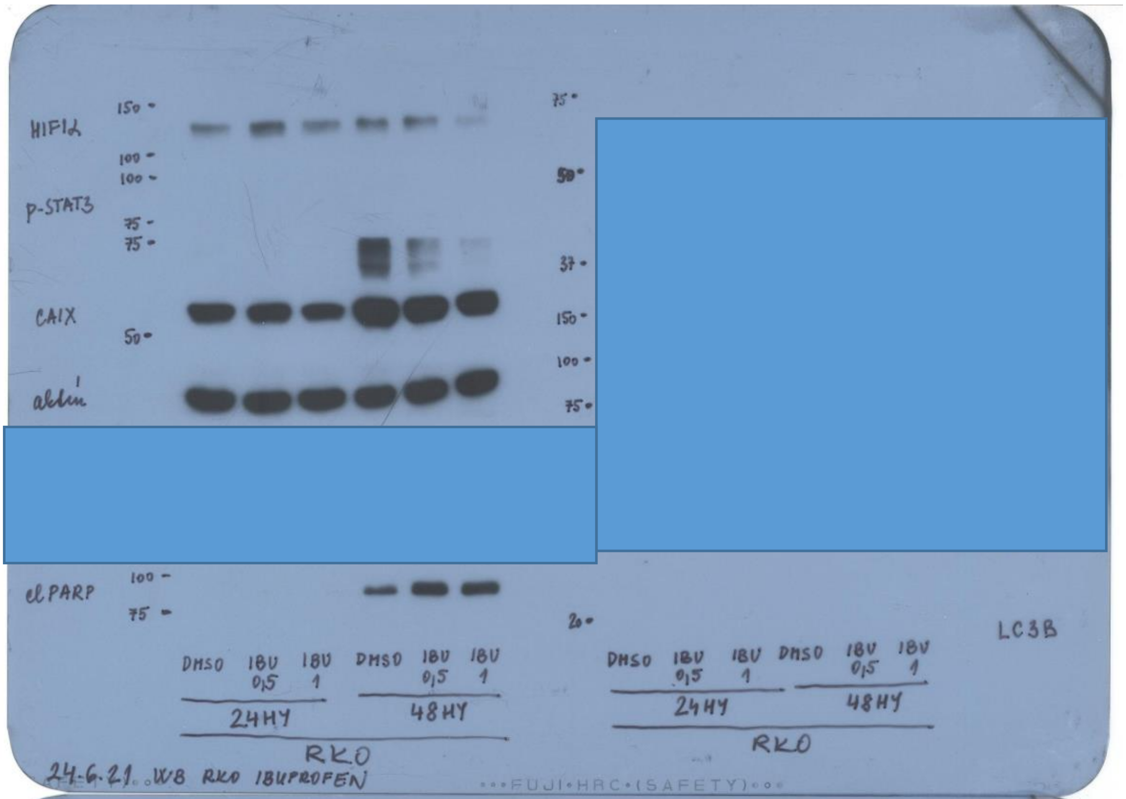

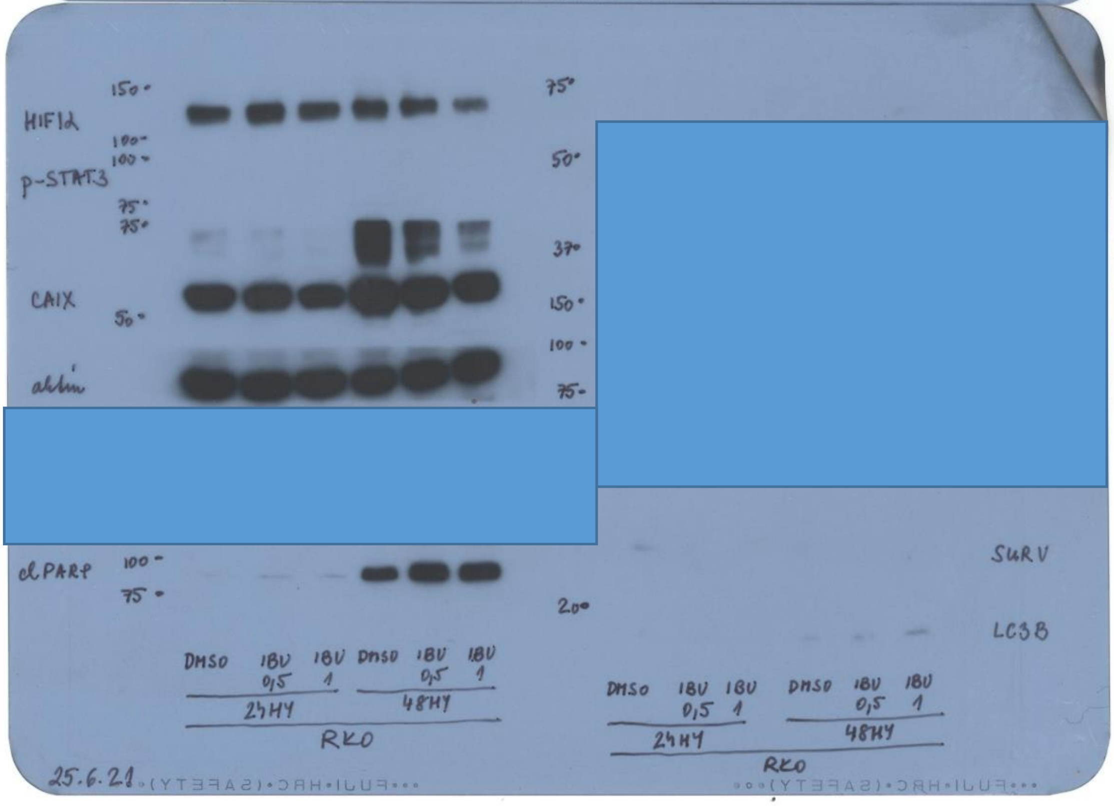

FaDu 24HY, 48HY – CA IX (Figure 1A), cl-PARP (Figure 5A),

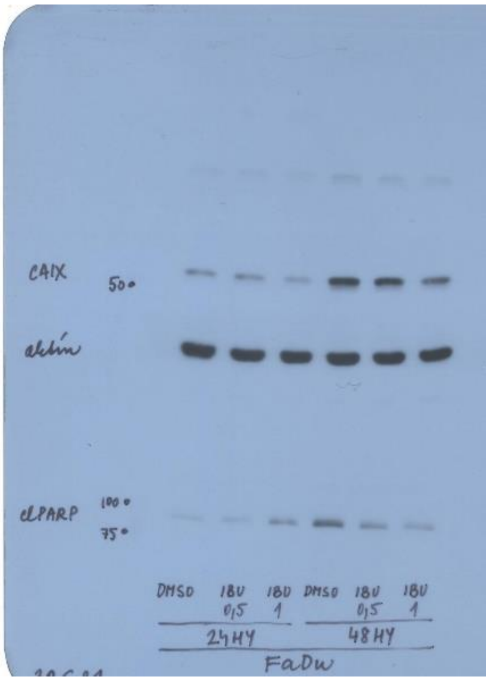

FaDu 24 HY, 48HY – HIF 1alpha (Figure 2C), p-STAT3 (Figure 2F), CA IX (Figure 1A), cl. PARP (Figure 5A), SURV = Survivin (Figure 3A – USED IN FIGURE)

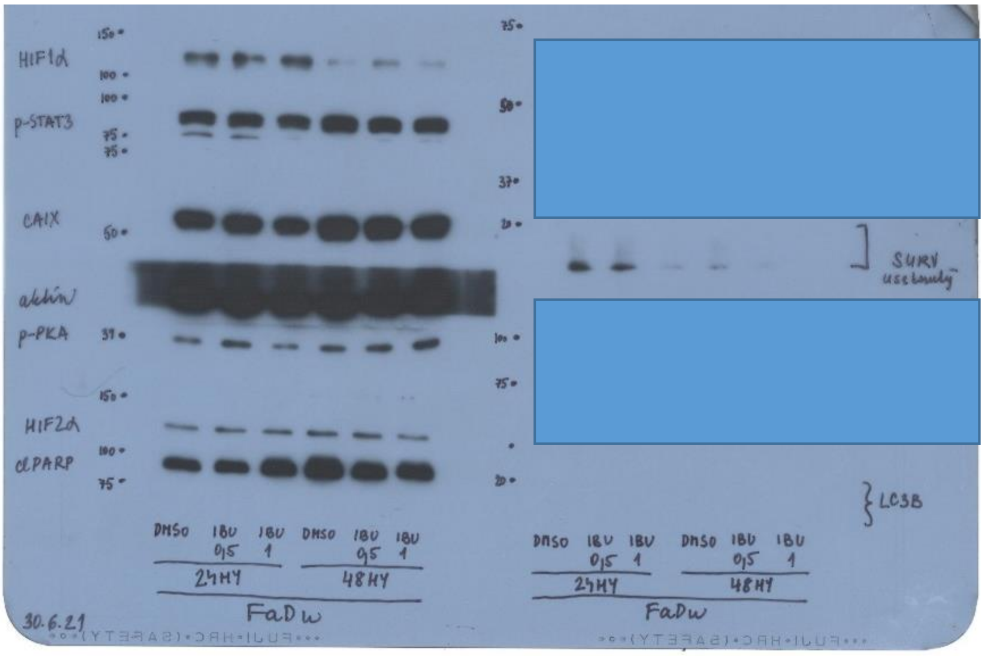

HCT116 3D – CA IX (Figure 1D – USED IN FIGURE – CONFIRMED BY IMMUNOHISTOCHEMISTRY (Figure 1E), aktín = actin (USED IN FIGURE 1D), cl. PARP (USED IN FIGURE 5B with actin)

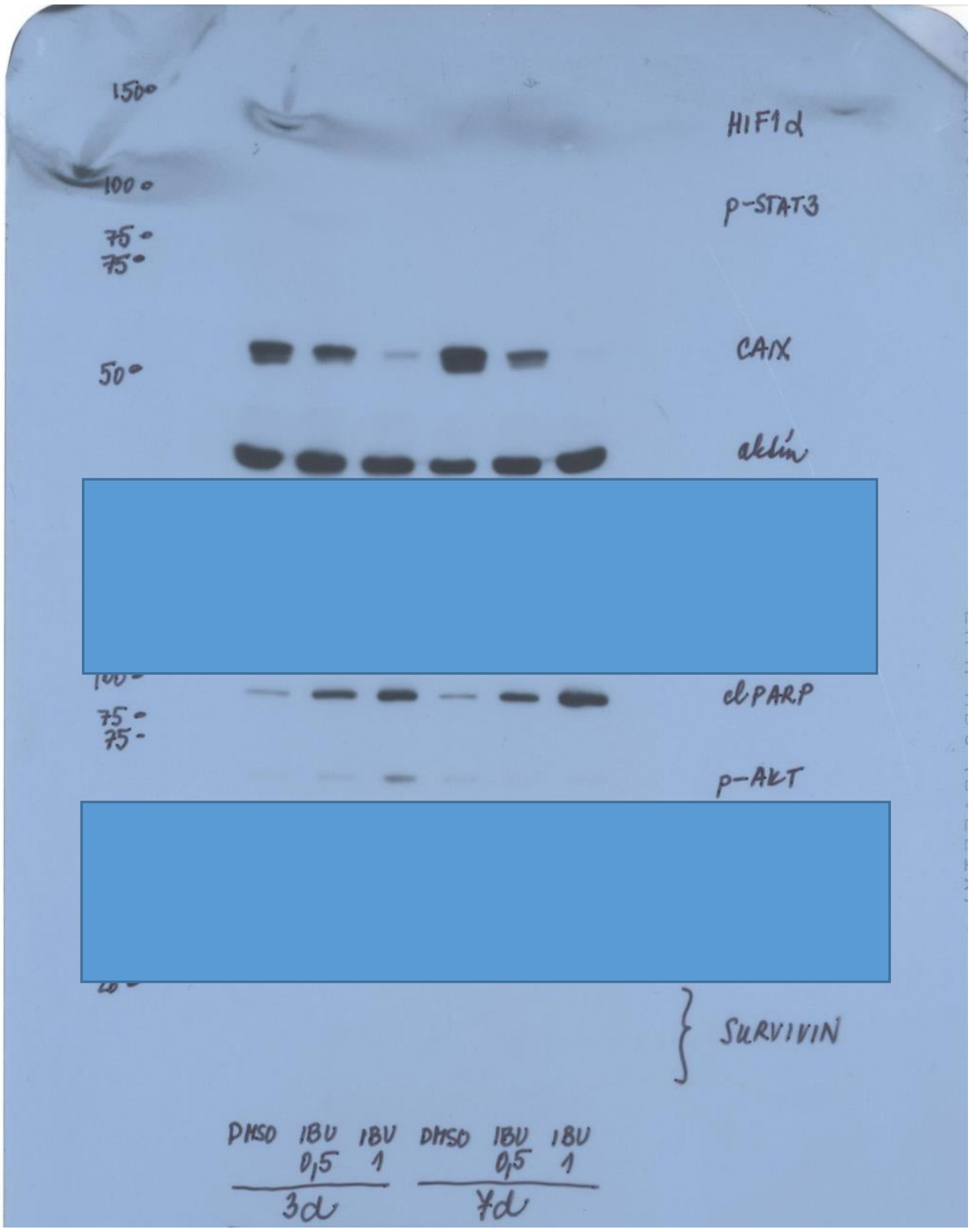

HCT116 3D – HIF-1alpha (Figure 2E – USED IN FIGURE 2E), CA IX (Figure 1D), downregulation of p-STAT3 in HCT116 cell line in 3D – not used in manuscript, but confirmed results from monolayer)

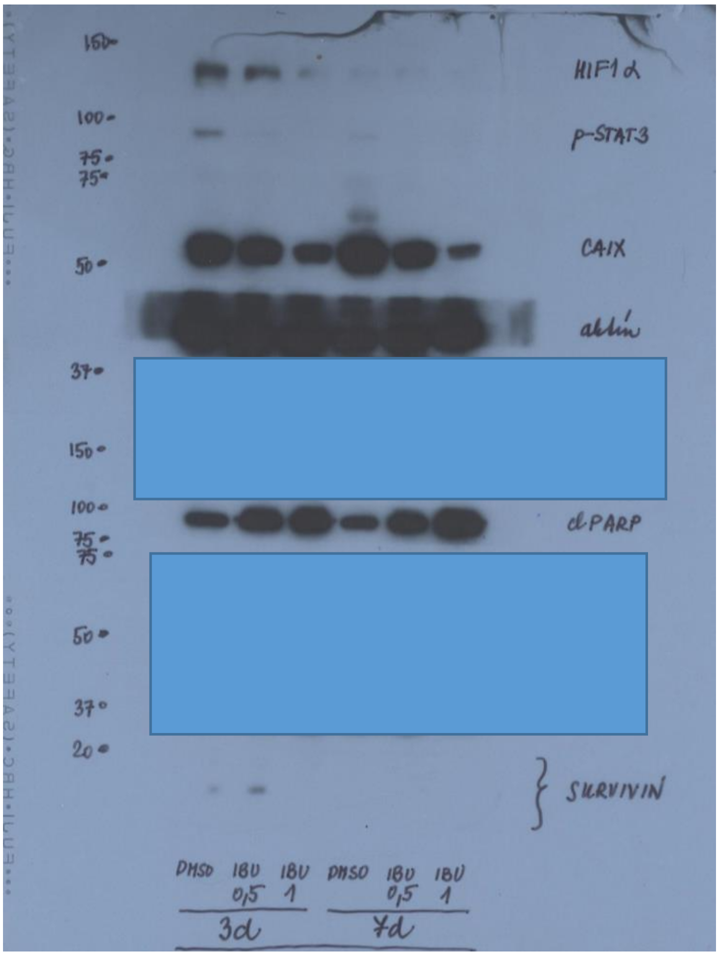

FaDu 3D – downregulation of p-STAT3 in FaDu 3D model – not used in manuscript, but confirmed results from monolayer , aktín = actin - USED IN Figure 1D to CA IX showe in next WB from the same lysate)

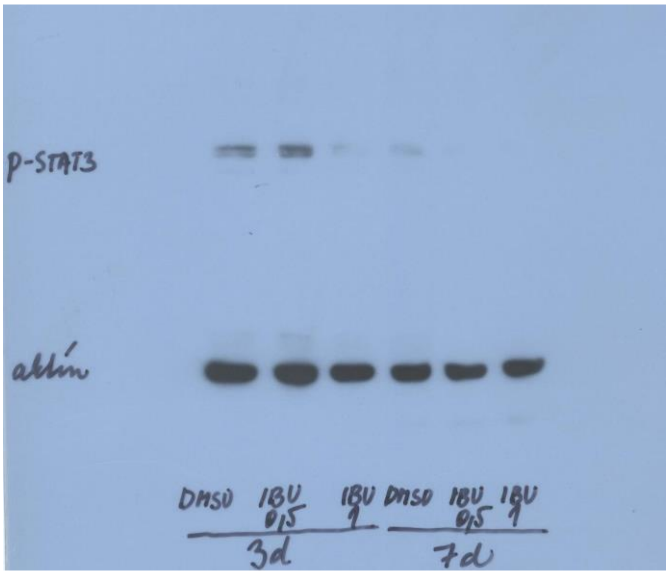

FaDu 3D – CA IX (USED IN FIGURE 1D – result was confirmed by immunohistochemistry in Figure 1E)

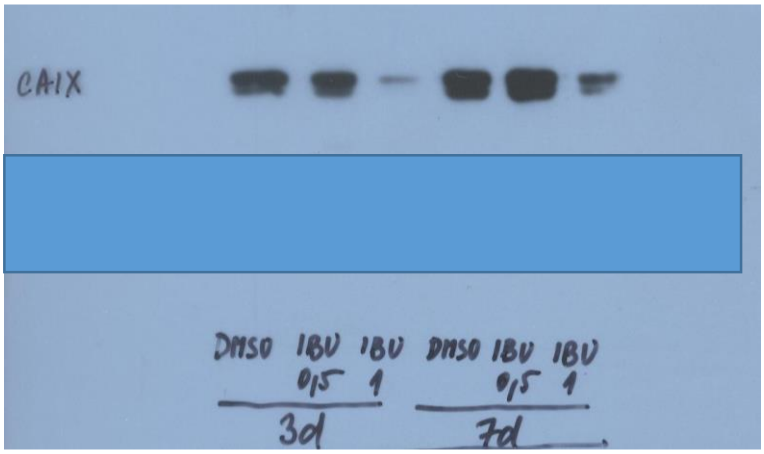

FaDu 3D – cl.PARP (USED IN FIGURE 5B), aktín = actin (USED IN FIGURE 5B)

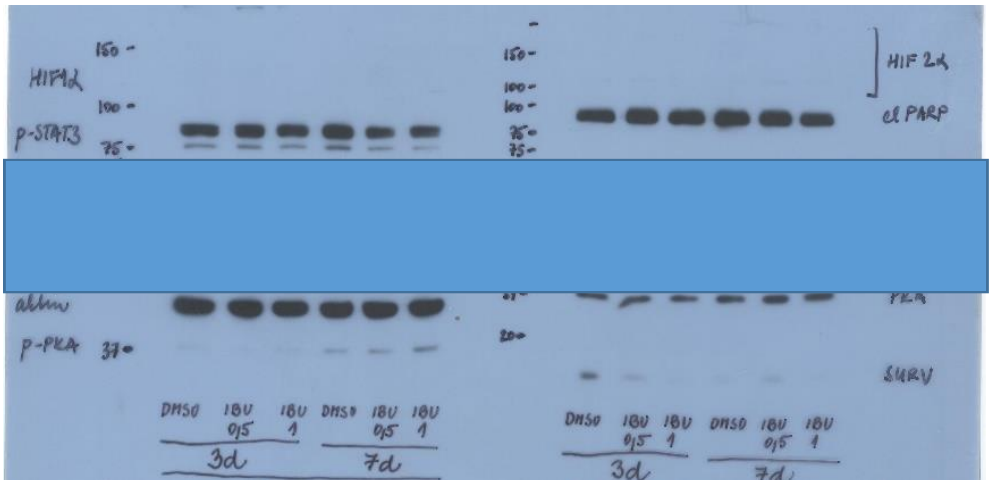

FaDu 3D – HIF-1alpha (Figure 2A – USED IN FIGURE)

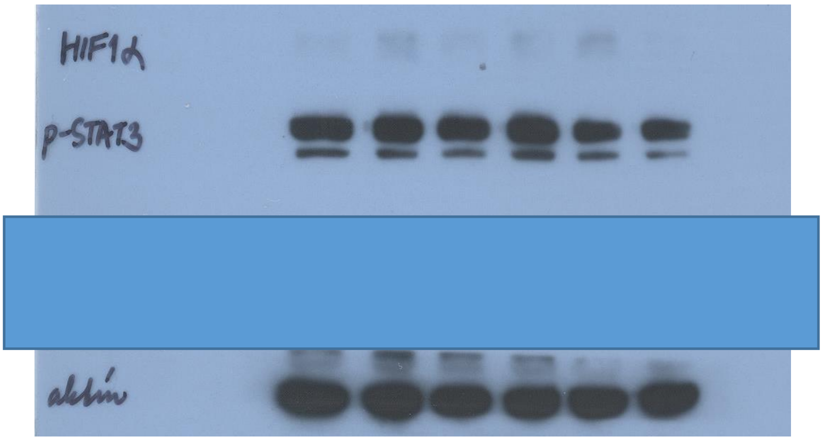

HCT116 48HY, RKO 48HY – HIF-1alpha (Figure 2C)

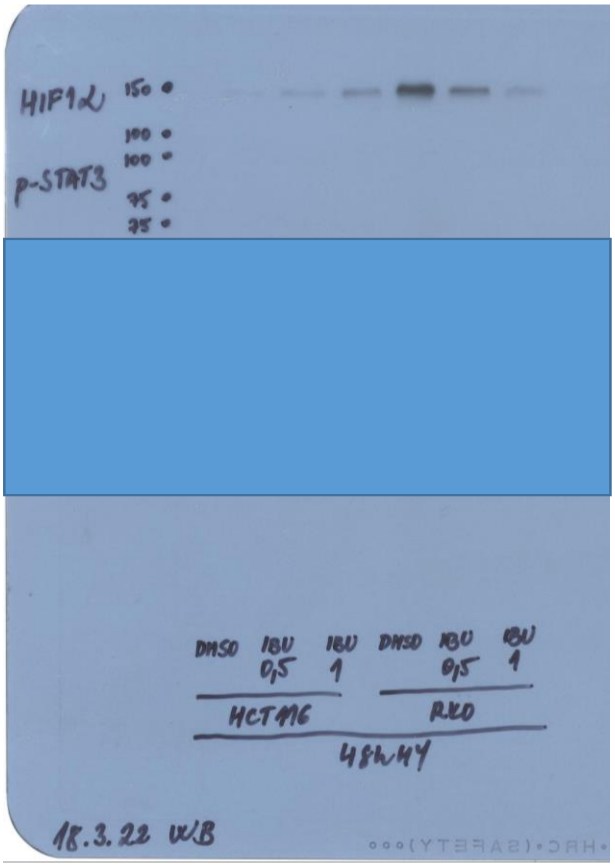

HCT116 48HY, RKO 48HY – HIF-1alpha (Figure 2C), pSTAT3 (Figure 2F)

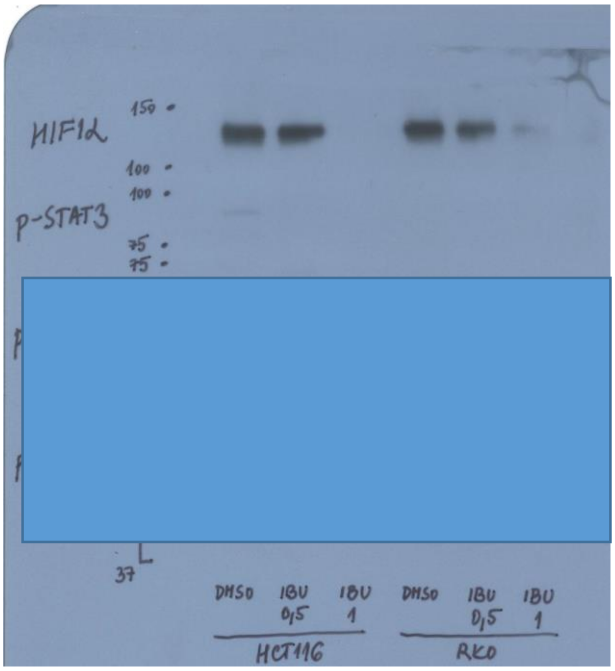

UM-22A – HIF-1alpha (Figure 2C – USED IN FIGURE), p-STAT3 (Figure 2F – USED IN FIGURE), cl. PARP (Figure 5A – USED IN FIGURE), CA IX (Figure 1A – USED IN FIGURE)

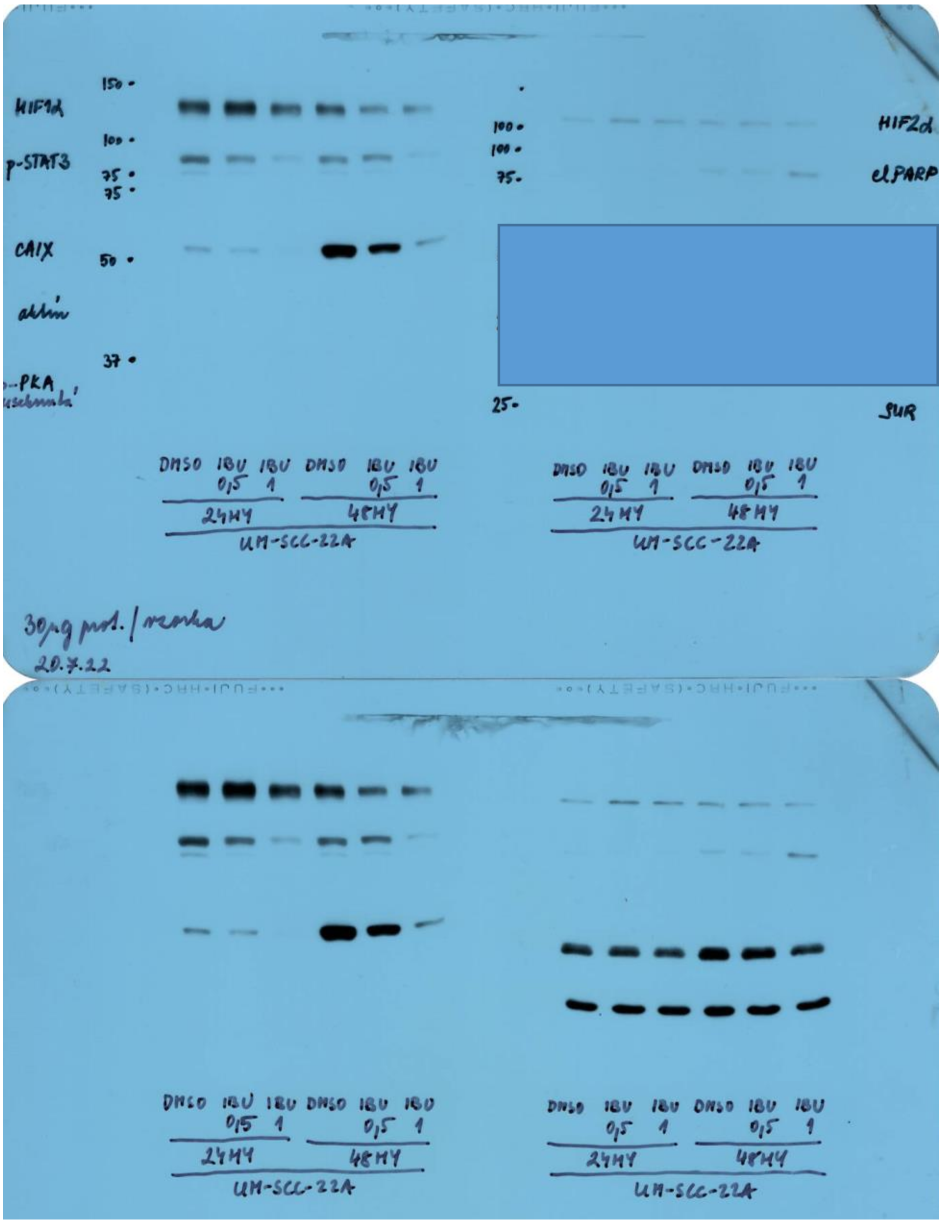

FaDu – Figure 5A (USED IN FIGURE)

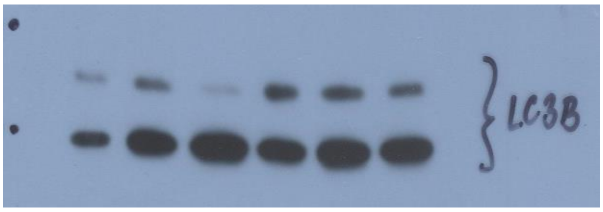

FaDu, UM-22A – LDHA (Figure 2D), COX2 – Figure 4B (COX2 UM-22A USED IN FIGURE 4B), NFkB p105 UM-22A – USED IN FIGURE 3A), NFkB p105 FaDu (Figure 3A)

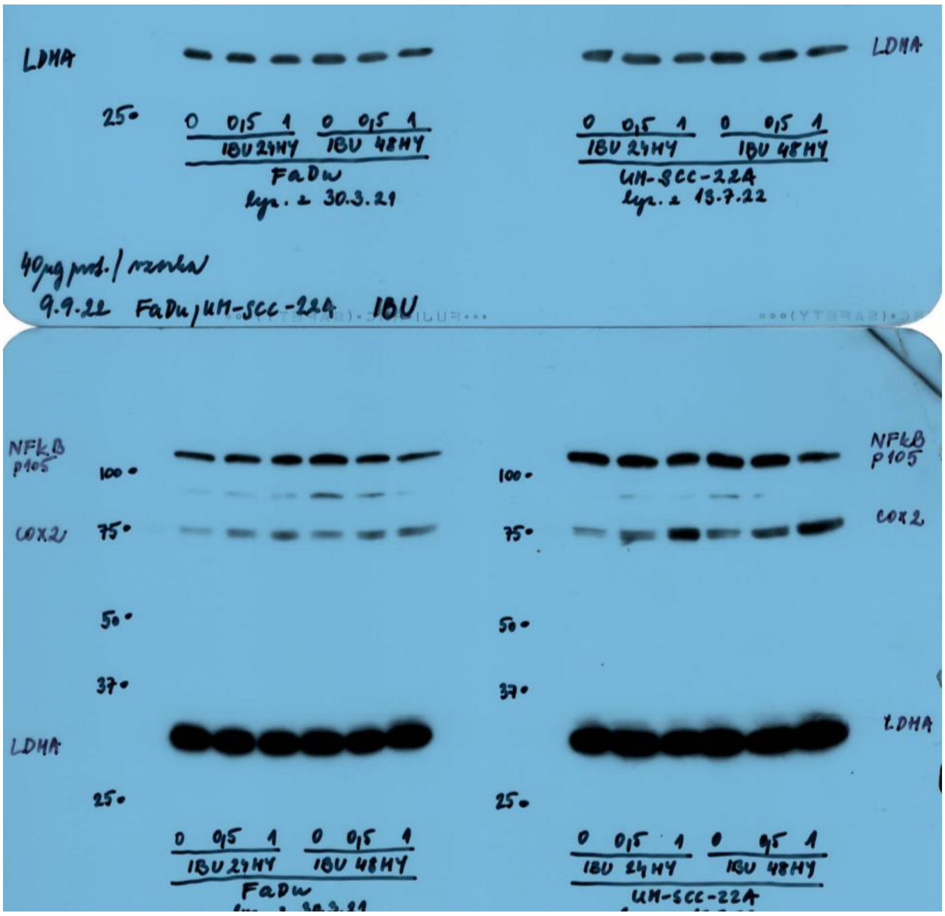

HCT116 – pAKT (Figure 4B). PDHK1 (Figure 2D – USED IN FIGURE 2D), LDHA (Figure 2D), NFkB p50 (Figure 3A)

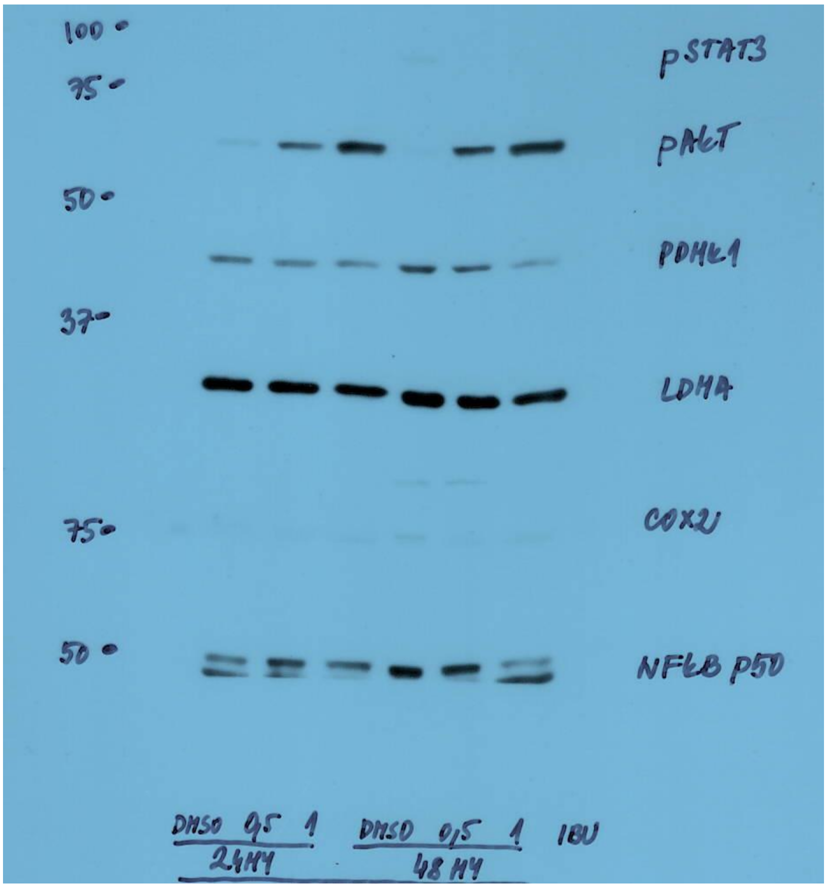

HCT116 - pSTAT3 (Figure 2F), p-AKT (Figure 4B)

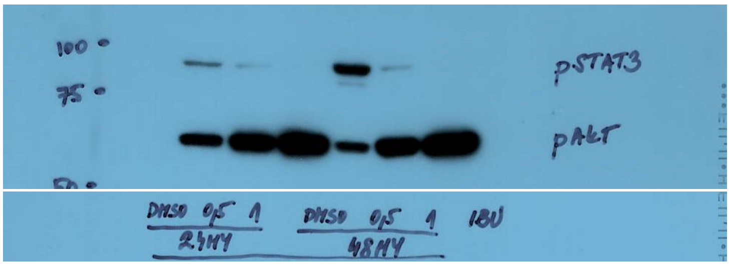

FaDu – HIF-1alpha (Figure 2C), COX2 (Figure 4B),

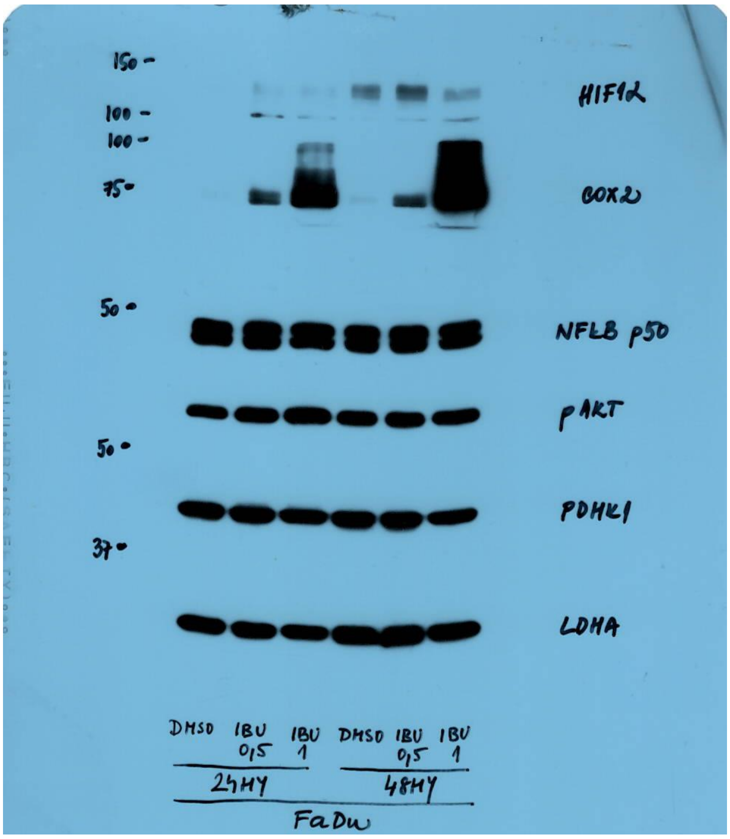

FaDu - COX2 (Figure 4B), NFkB p50 (Figure 3A – USED IN FIGURE), pAKT (Figure 4B), PDHK1 (Figure 2D), LDHA (Figure 2D)

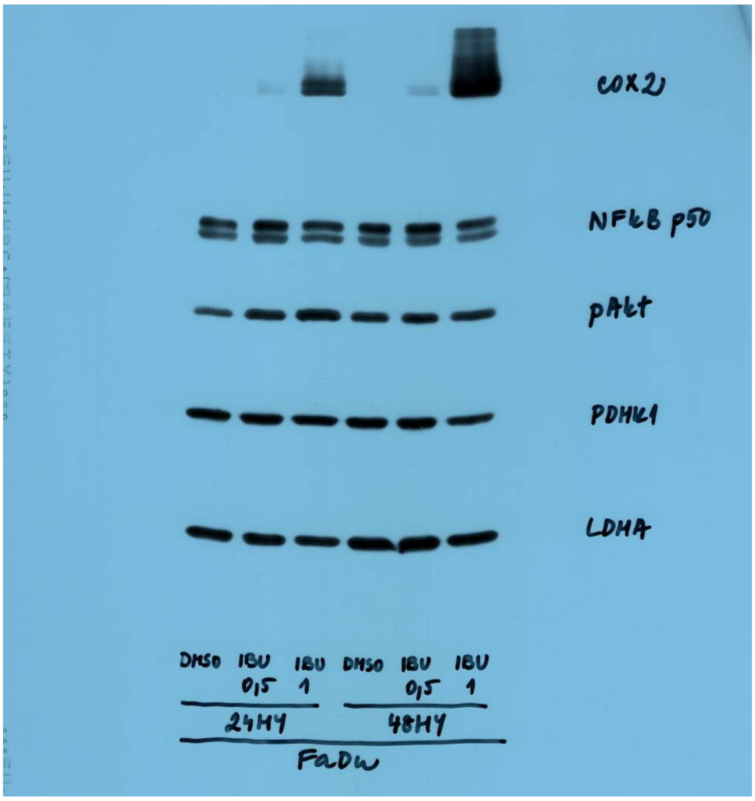

RKO – NFkB p50 (Figure 3A – USED IN FIGURE), pAKT (Figure 4B – USED IN FIGURE), PDHK1

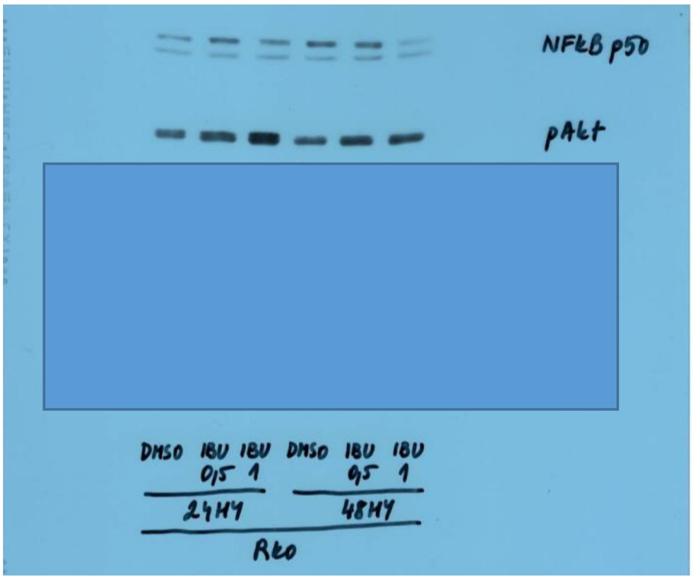

RKO – HIF-1alpha (Figure 2C – USED IN FIGURE), COX2 (Figure 4B – USED IN FIGURE), NFkB p50 (Figure 3A), pAKT (Figure 4B),

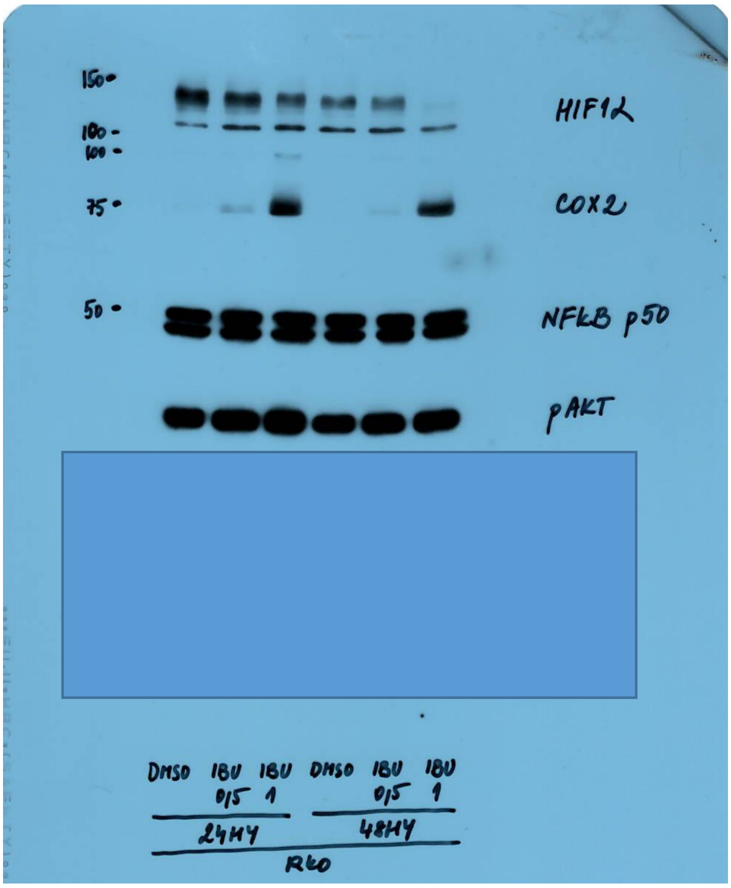

RKO, FaDu – NFkB p105 (Figure 3A – USED IN FIGURE), pSTAT3 (Figure 2F – USED IN FIGURE), aktín = actin FaDu (USED IN FIGURE 2F), CA IX RKO (Figure 1A – USED IN FIGURE), CA IX FaDu (Figure 1A – USED IN FIGURE)

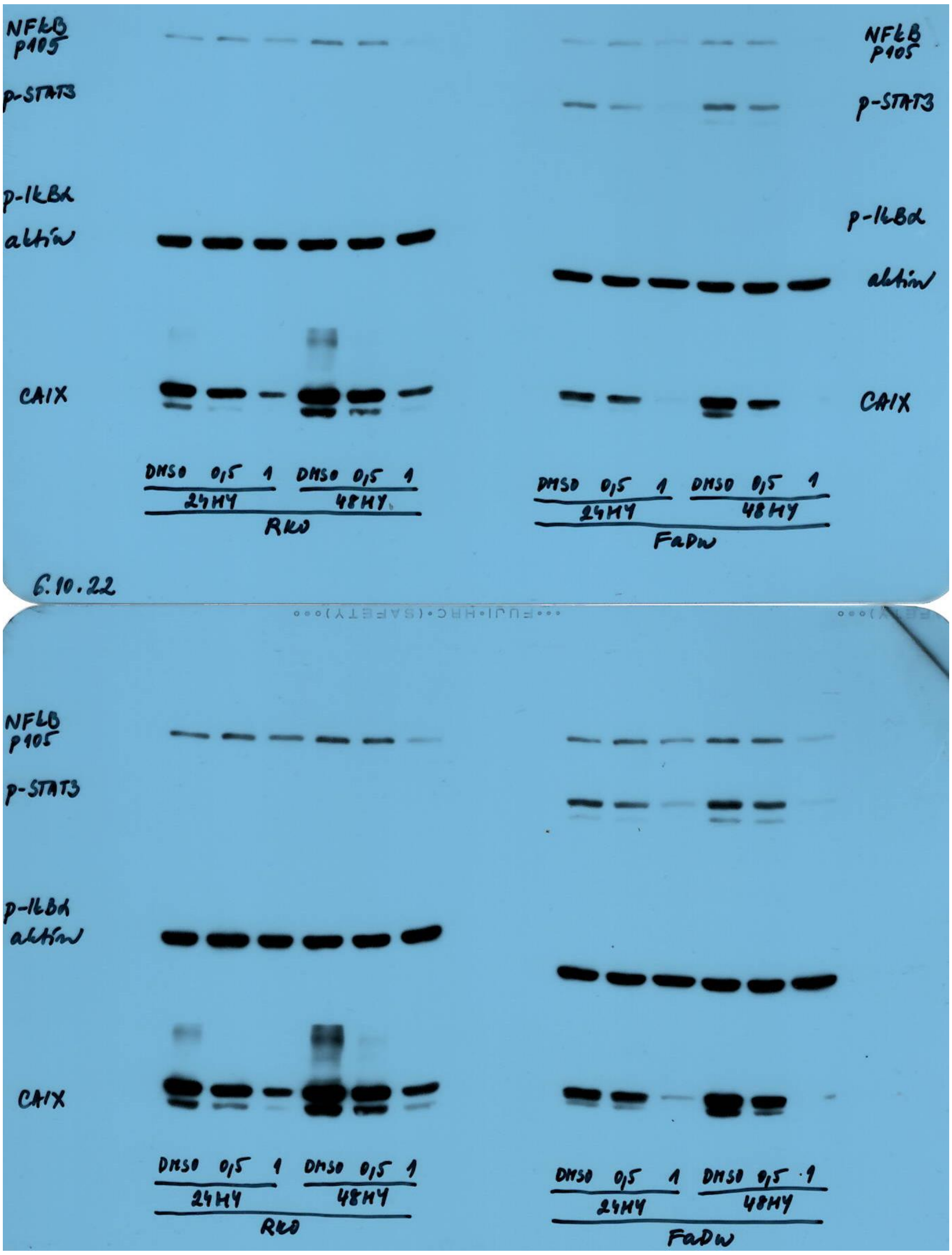

FaDu – STAT3 (Figure 2F), pAKT (Figure 4B), NFkB p50 (Figure 3A – USED IN FIGURE), LDHA (Figure 2D – USED IN FIGURE), HIF-1alpha (Figure 2C – USED IN FIGURE)

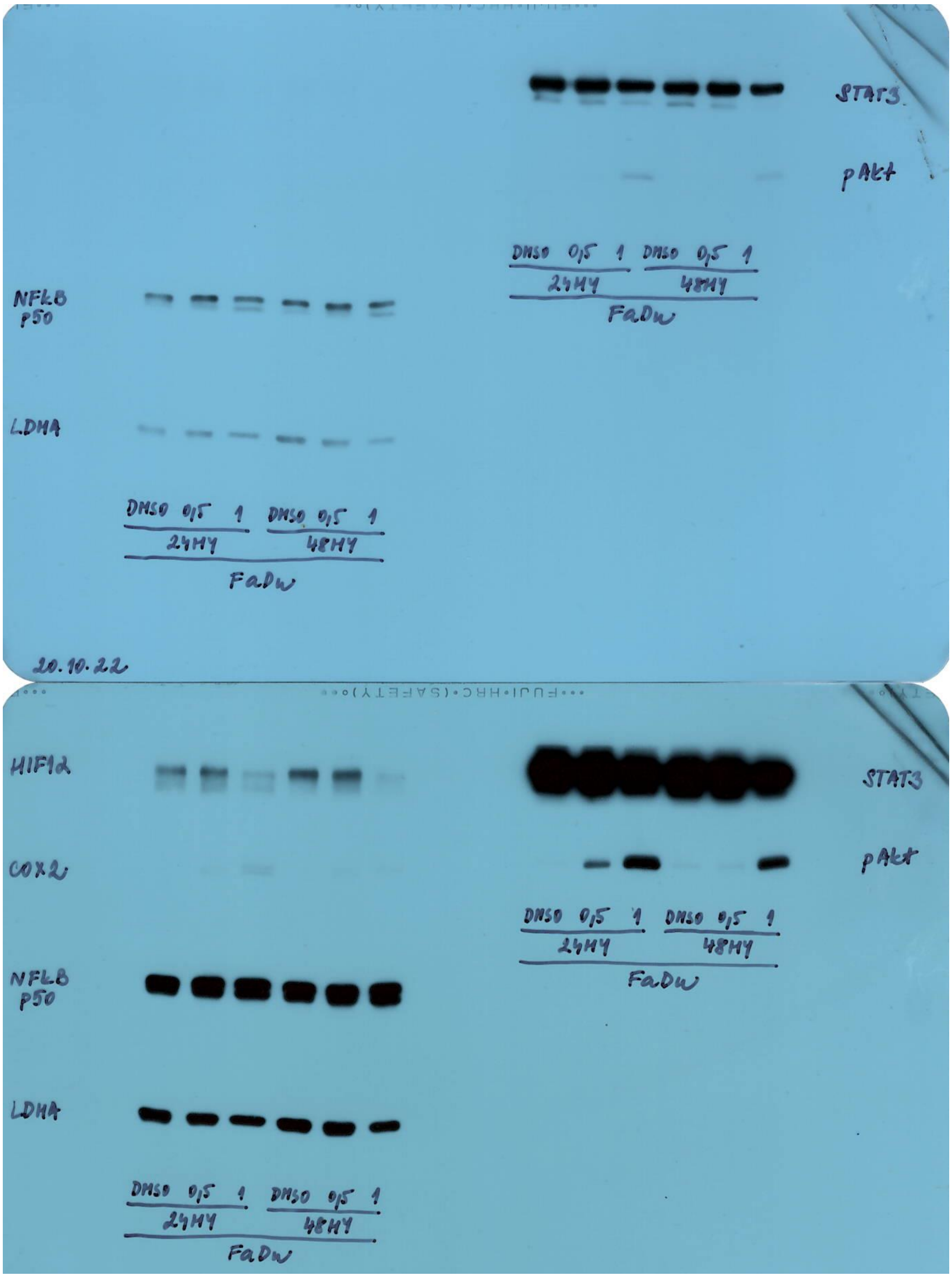

FaDu – HIF-1alpha (Figure 2C), COX2 (Figure 4B – USED IN FIGURE), LDHA (Figure 2D), pAKT (Figure 4B), NFkB p105 (Figure 3A – USED IN FIGURE), STAT3 (Figure 2F – USED IN FIGURE)

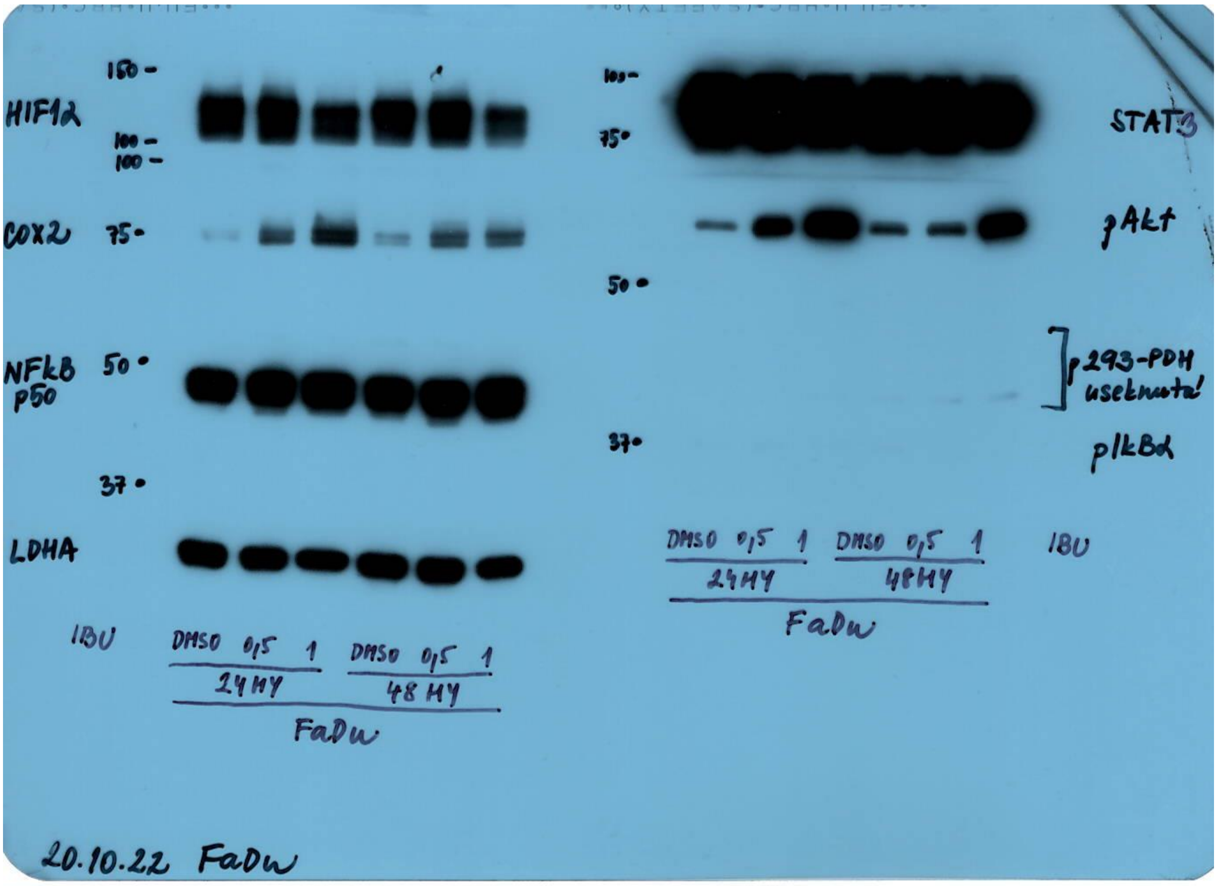

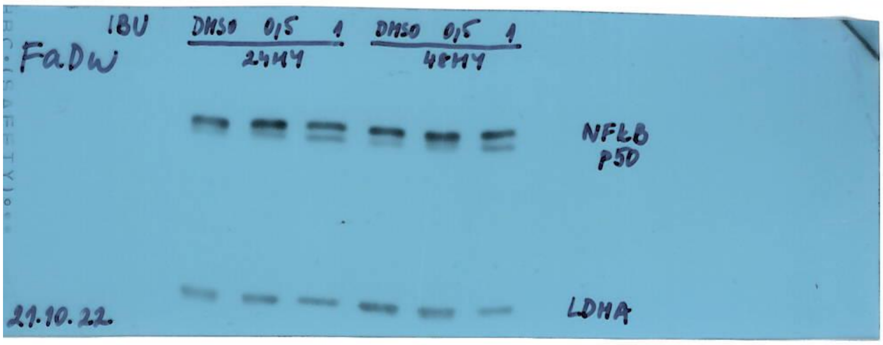

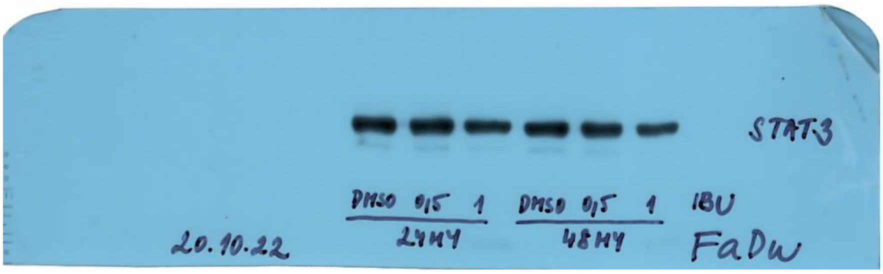

HCT116 – NFkB p105 (Figure 3A – USED IN FIGURE), STAT3 (Figure 2F – USED IN FIGURE), pAKT (Figure 4B – USED IN FIGURE), LDHA (Figure 2D – USED IN FIGURE)

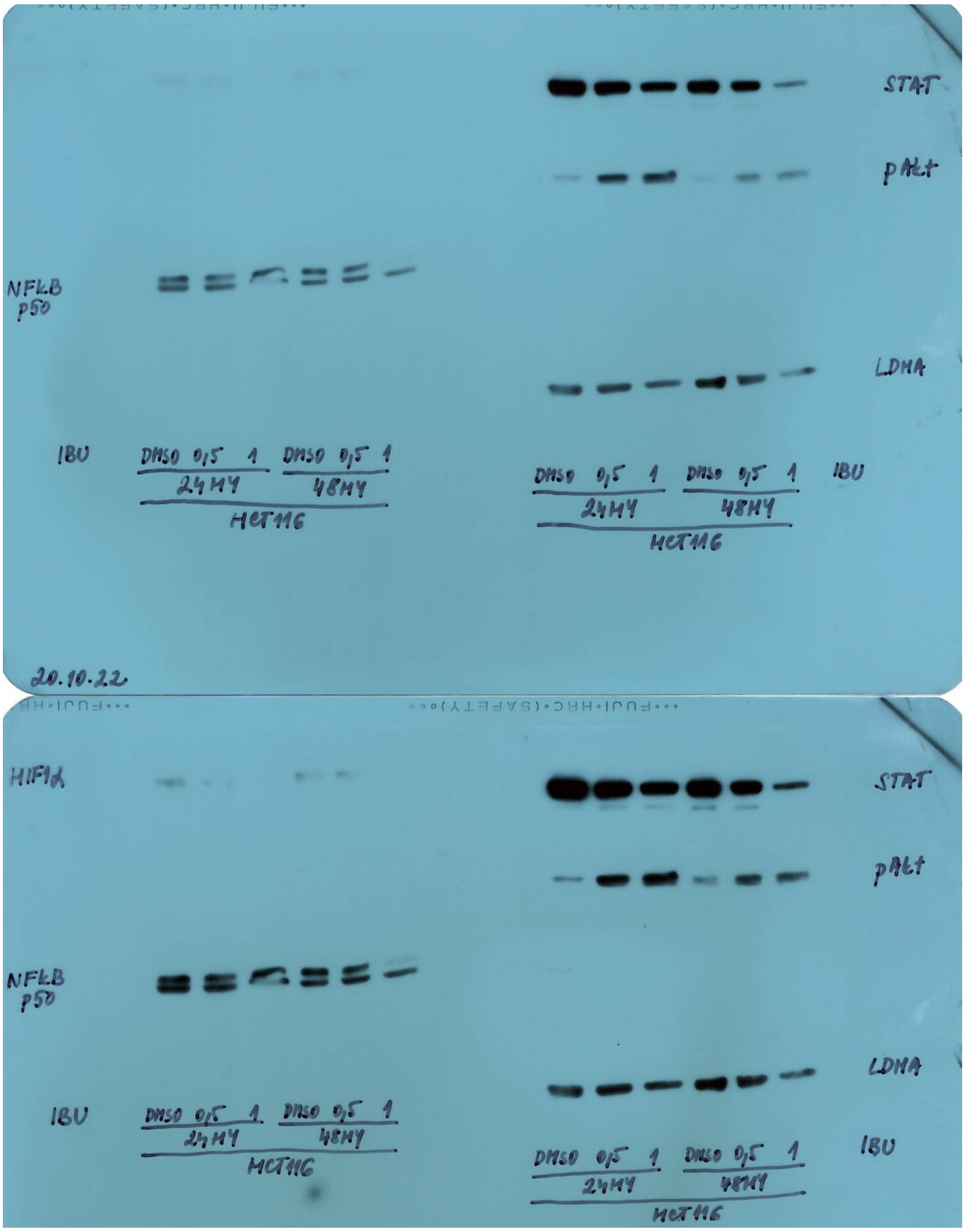

HCT116 – HIF-1alpha (Figure 2C), COX2 (Figure 4B), NFkB p50 (Figure 3A), STAT3 (Figure 2F), LDHA (Figure 2D)

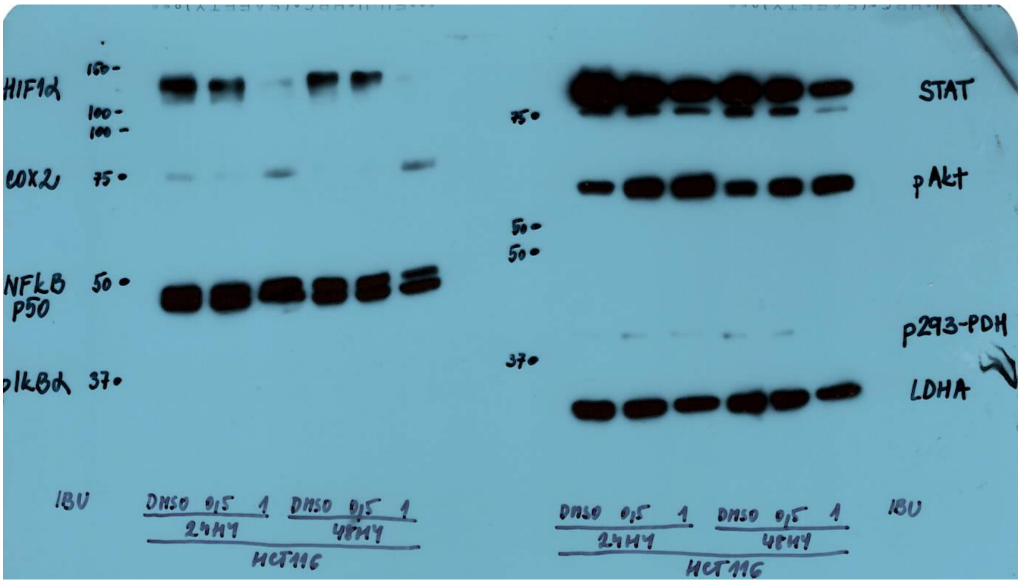

HCT116, FaDu – NFkB p105 (Figure 3A – USED IN FIGURE), CA IX (Figure 1A), PDHK1 (Figure 2D – USED IN FIGURE), NFkB p50 (Figure 3A – FaDu – USED IN FIGURE), pERK (Figure 4B – USED IN FIGURE)

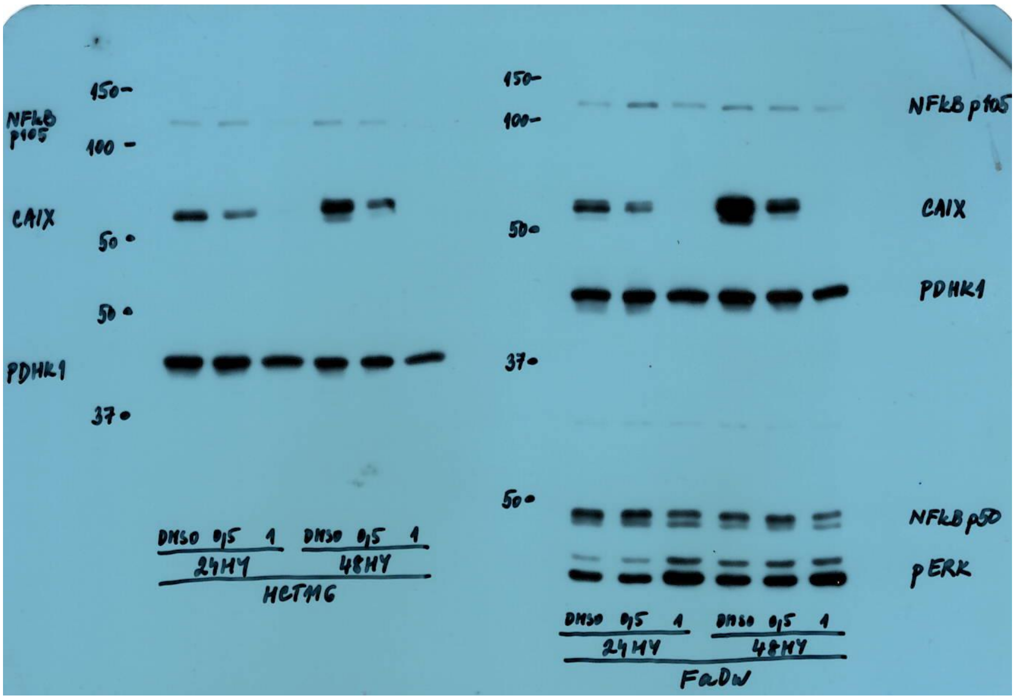

RKO, UM-22A – STAT3 (Figure 2F – USED IN FIGURE)

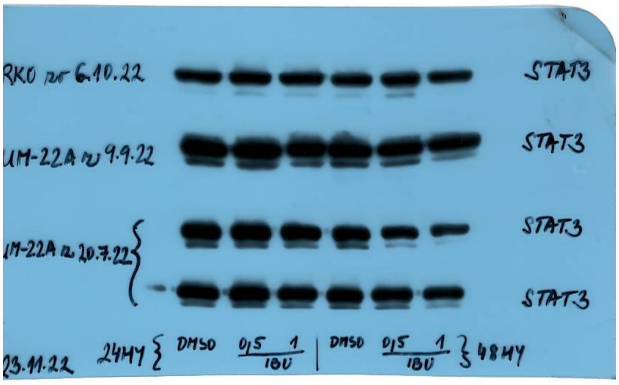

HCT116, RKO, FaDu, UM-22A – IkBa (Figure 3A – USED IN FIGURE)

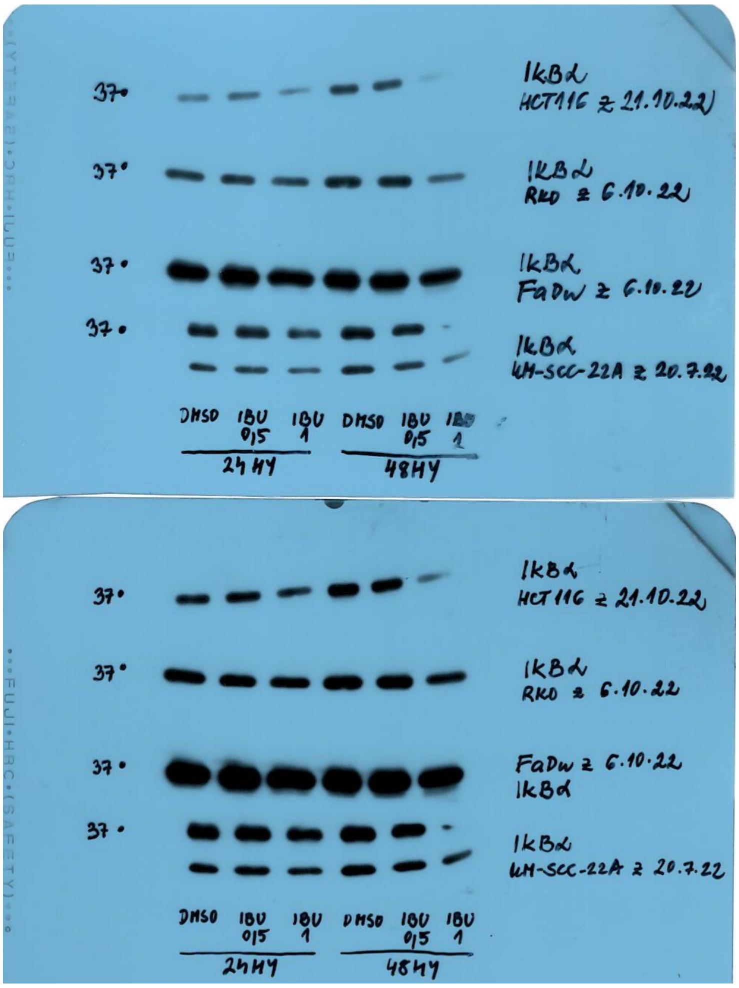

HCT116 – AKT (Figure 4B – USED IN FIGURE), pSer9-GSK3b (Figure 4B – USED IN FIGURE)

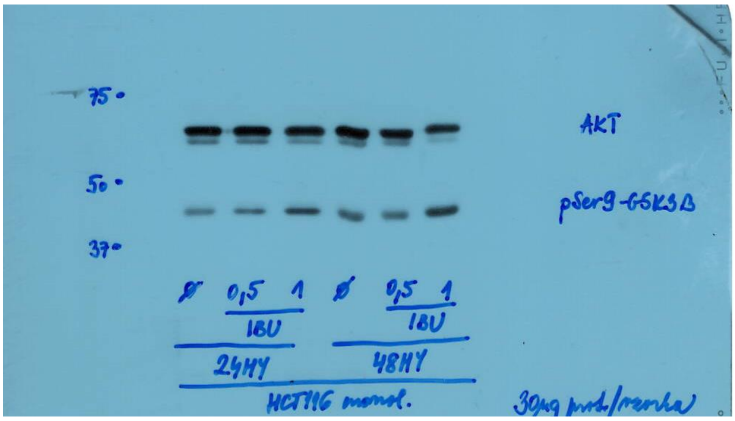

HCT116, RKO – NFkB p50 (Figure 3B)

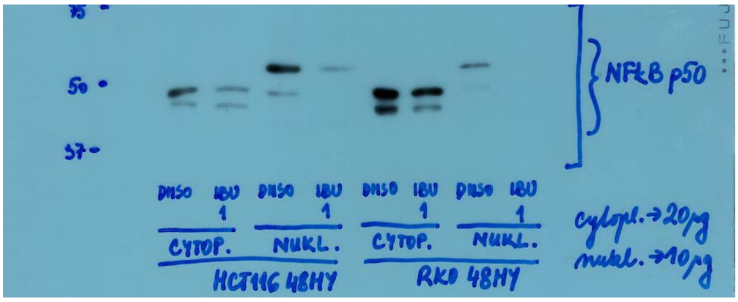

FaDu – cl.PARP (Figure 5A – USED IN FIGURE), AKT (Figure 4B – USED IN FIGURE), pS9-GSK3b (Figure 4B – USED IN FIGURE), LC3B (Figure 5A)

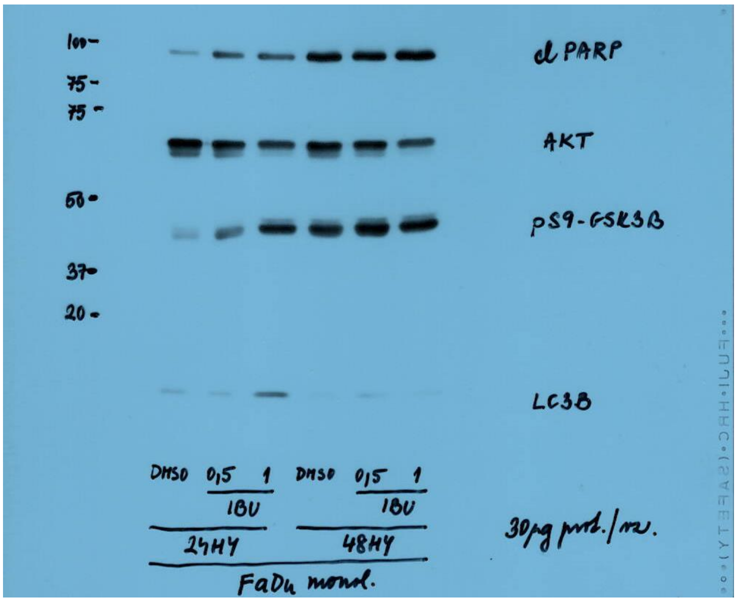

FaDu – cl.PARP (Figure 5A), AKT (Figure 4B), pS9-GSK3b (Figure 4B), LC3B (Figure 5A – USED IN FIGURE)

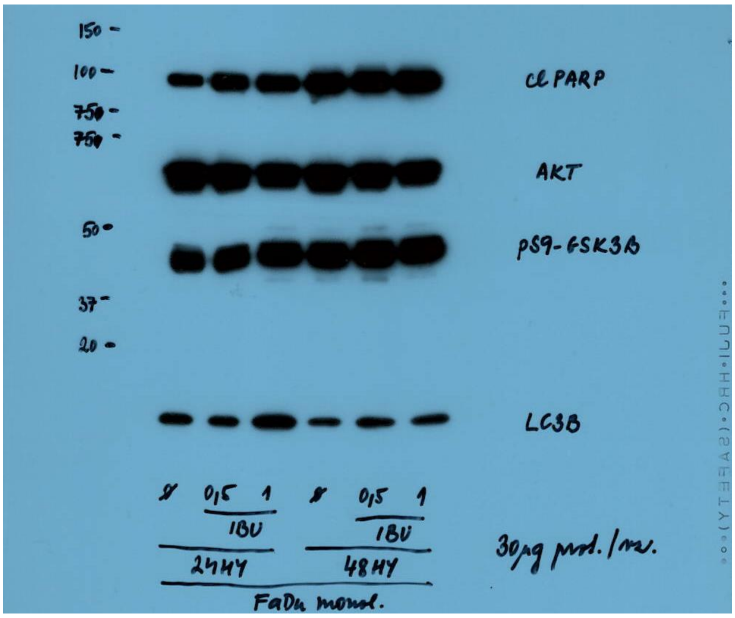

FaDu, UM-22A – NFkB p50 (Figure 3B)

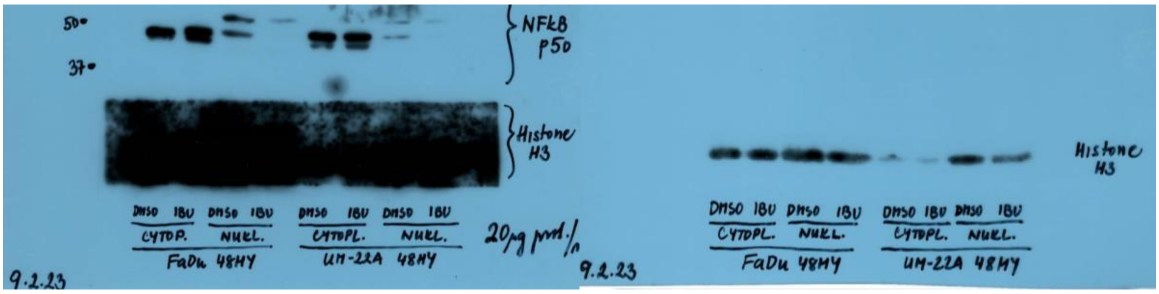

RKO, FaDu – pAKT (Figure 4C), pERK (Figure 4C), COX2 (Figure 4C)

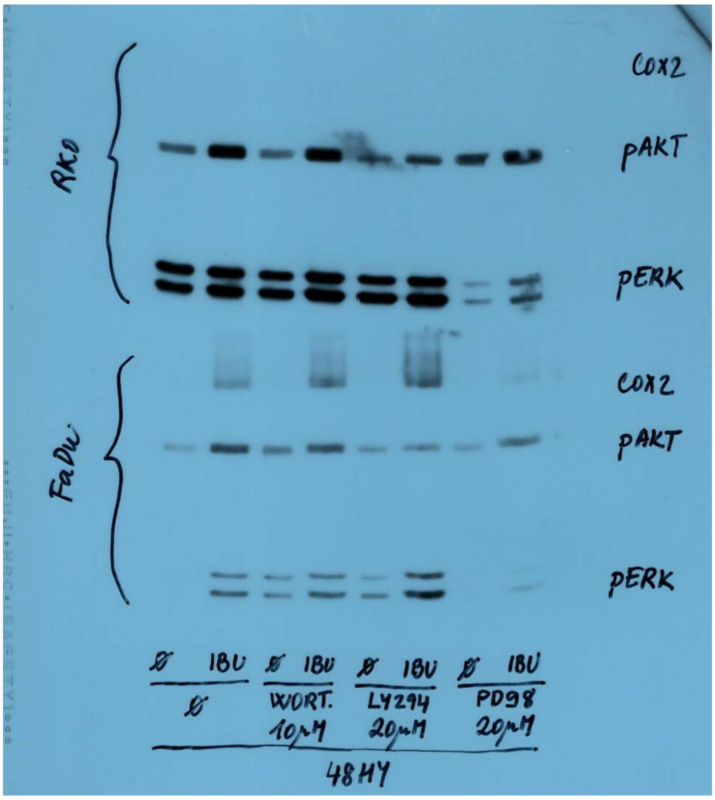

RKO – pS9-GSK3beta (Figure 4B – USED IN FIGURE), LC3B (Figure 5A), cl.PARP (Figure 5A), AKT (Figure 4B), p-ERK ½ (Figure 4B)

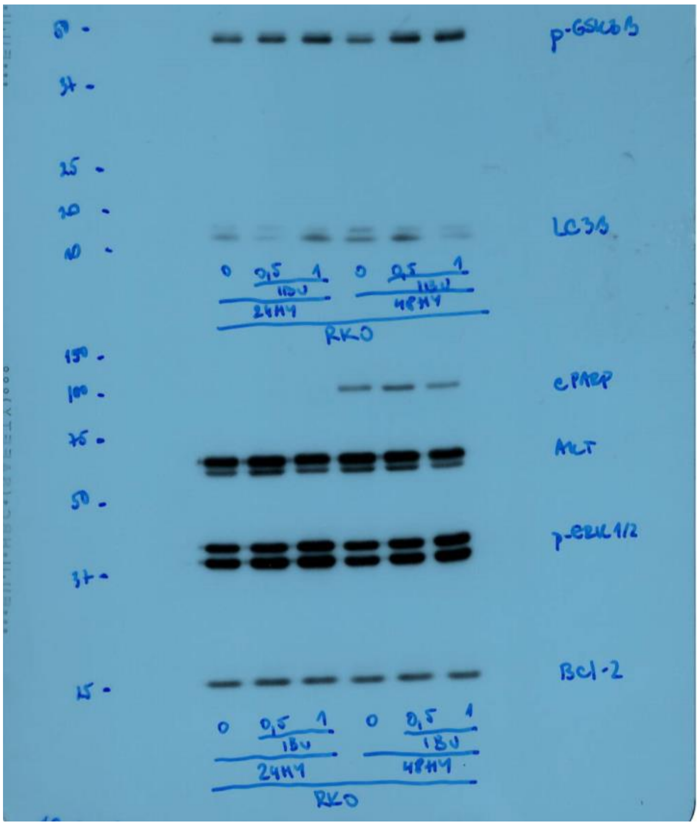

RKO – AKT (Figure 4B – USED IN FIGURE), p-ERK ½ (Figure 4B – USED IN FIGURE)

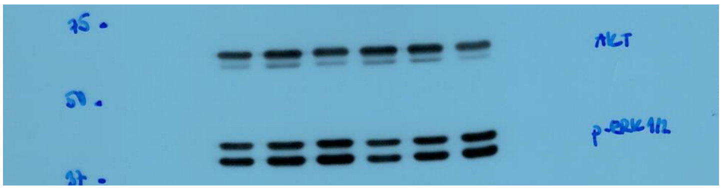

UM-22A – AKT (Figure 4B – USED IN FIGURE), pS9-GSK3beta (Figure 4B – USED IN FIGURE), cl. PARP (Figure 5A), p-AKT (Figure 4B), p-ERK ½ (Figure 4B – USED IN FIGURE)

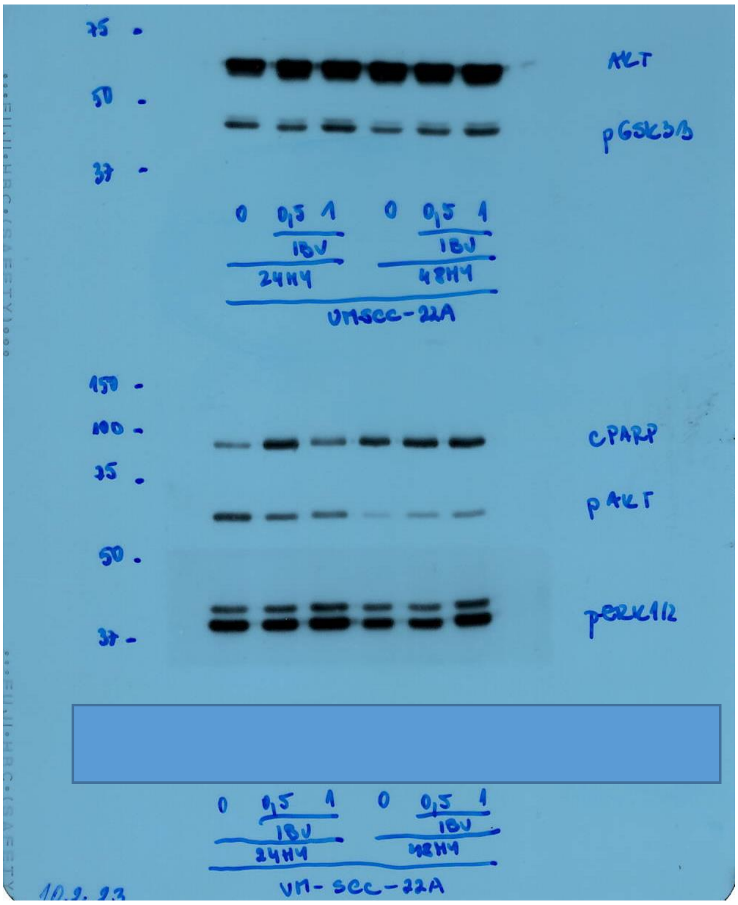

HCT116, UM-22A - p-AKT (Figure 4C – HCT116 - USED IN FIGURE), p-ERK ½ (Figure 4C)

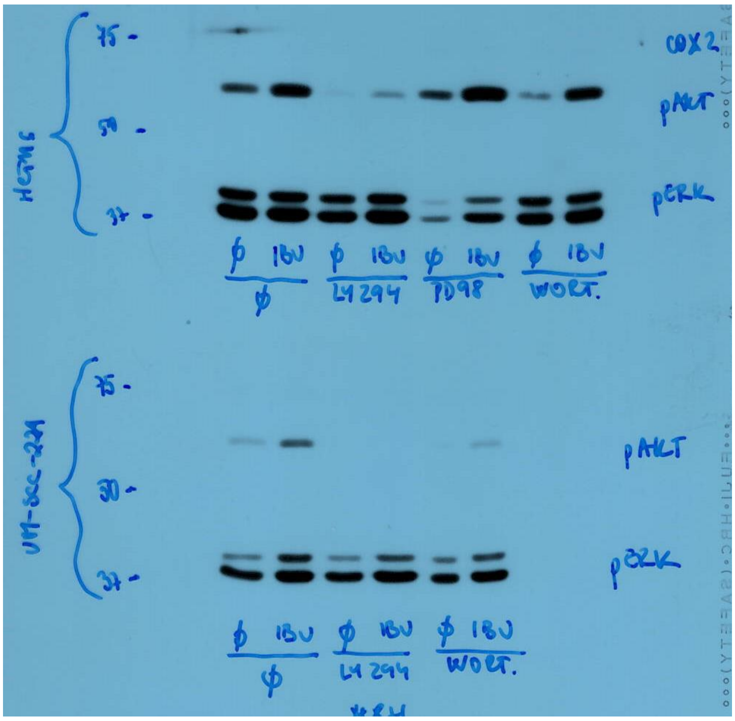

HCT116, UM-22A – aktín = actin (HCT116 Figure 4C – USED IN FIGURE)

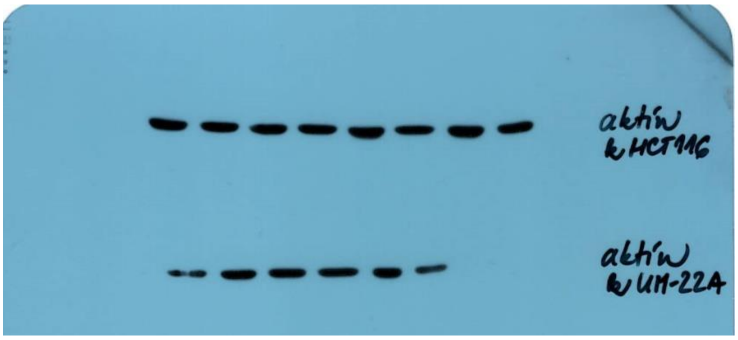

RKO – total ERK (Figure 4B – USED IN FIGURE)

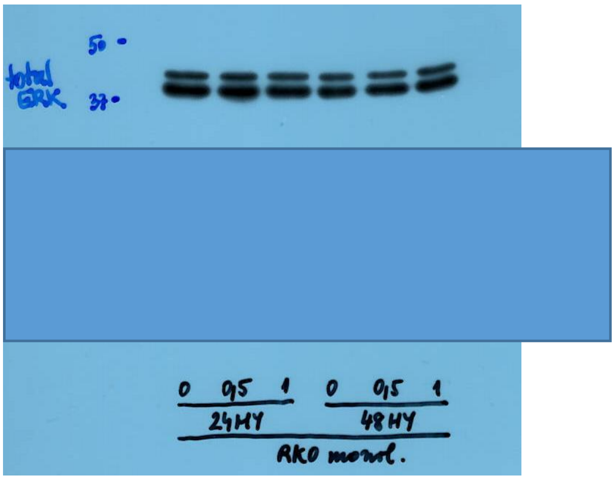

UM-22A – cl. PARP (Figure 5A), total ERK (Figure 4B – USED IN FIGURE), NFkB p50 (Figure 3A)

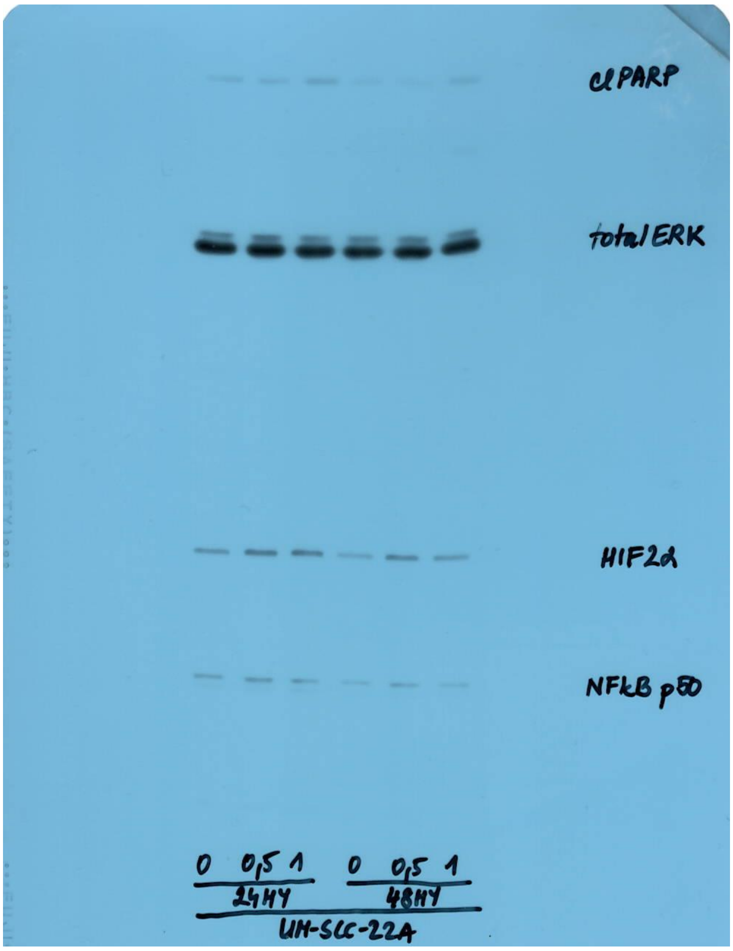

UM-22A – cl. PARP (Figure 5A), pAKT (Figure 4B), total ERK (Figure 4B), NFkB p50 (Figure 3A – USED IN FIGURE), SURV (Figure 3A)

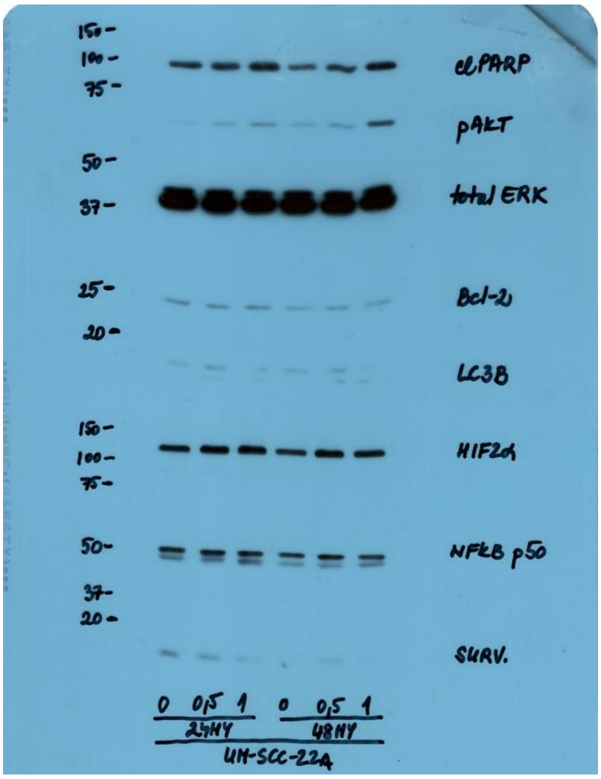

UM-22A – pAKT (Figure 4B – USED IN FIGURE)

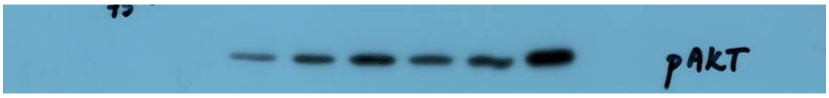

HCT116, RKO– NFkB p50 (Figure 3B – NUK= nuclear extracts)

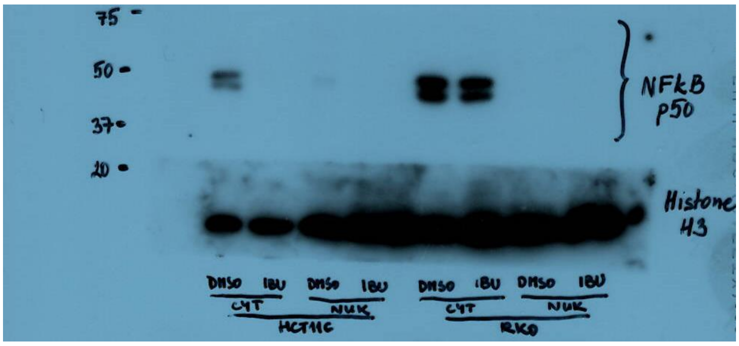

FaDu, UM-22A - (Figure 3B – NUK= nuclear extracts)

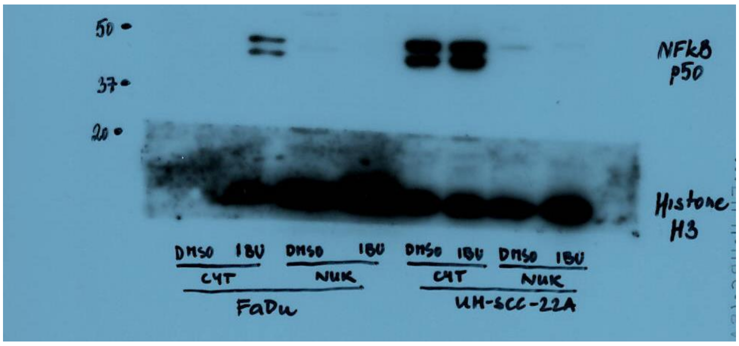

HCT116, RKO – pAKT (Figure 4C – RKO USED IN FIGURE), pERK ½ (Figure 4C – HCT116 and RKO USED IN FIGURE)

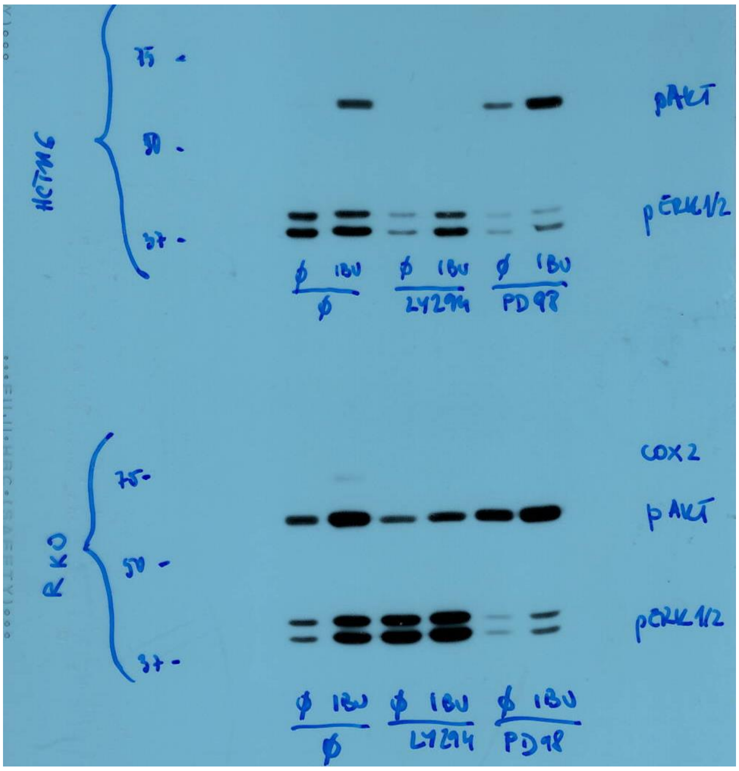

HCT116, UM-22A – COX2 Figure 4C (UM-22A USED IN FIGURE)

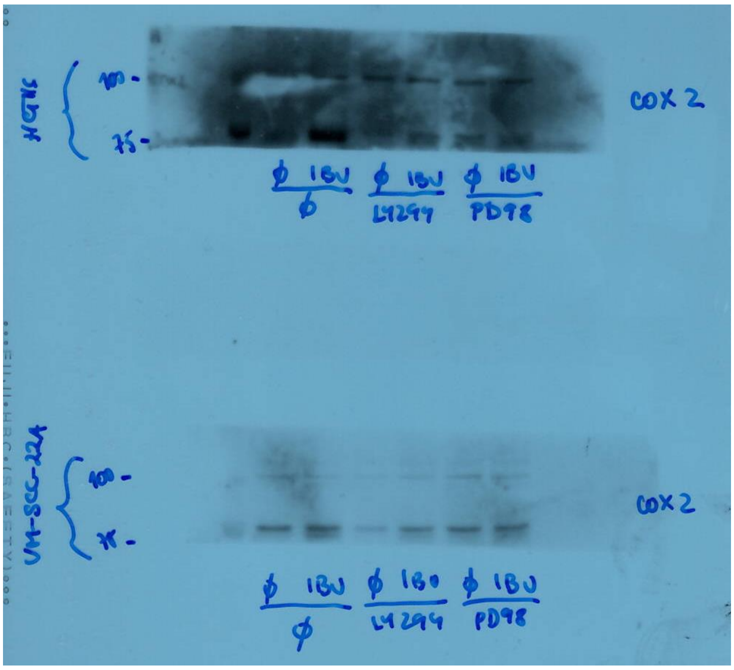

UM-22A – pAKT (Figure 4C – USED IN FIGURE), pERK ½ (Figure 4C – USED IN FIGURE)

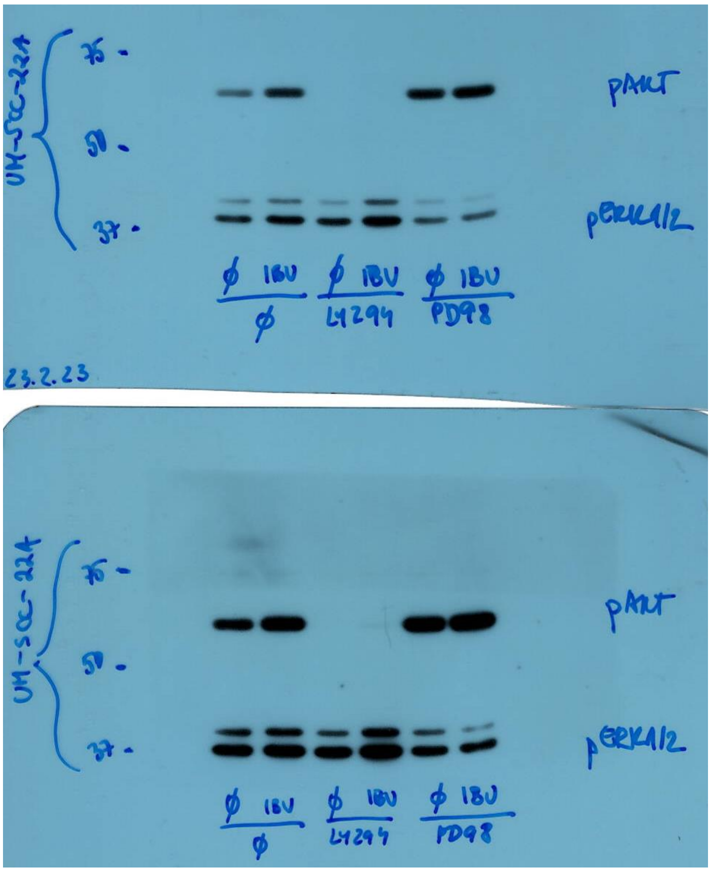

UM-22A, RKO – pERK (Figure 4C – USED IN FIGURE), betaAct = actin UM-22A (Figure 4C - USED IN FIGURE)

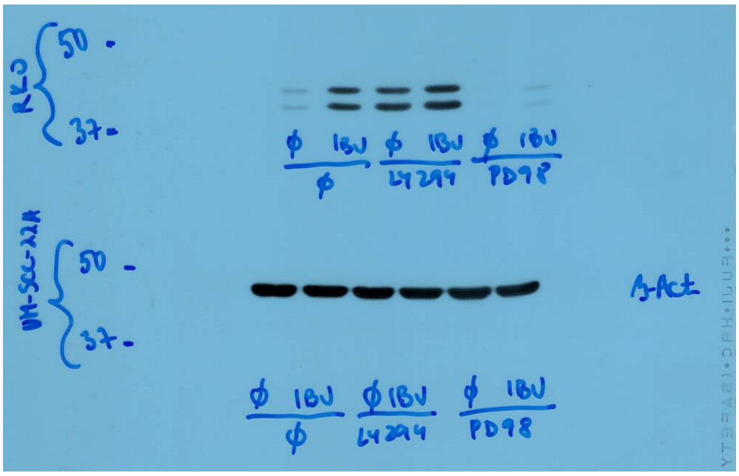

HCT116, UM-22A – COX2 (Figure 4C)

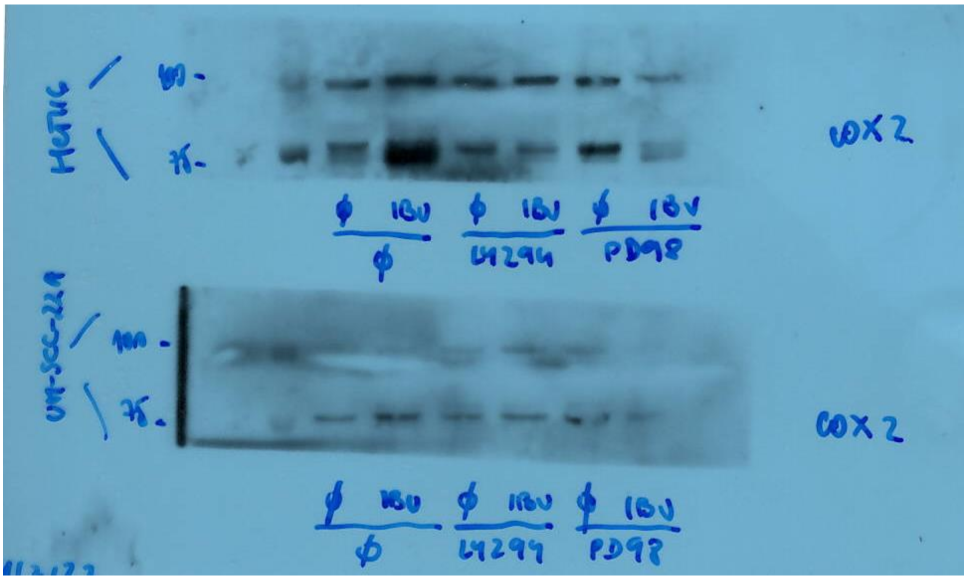

RKO, FaDu – above - betaAct = actin RKO (Figure 4C – USED IN FIGURE), bottom betaAct = actin FaDu (Figure 4C – USED IN FIGURE)

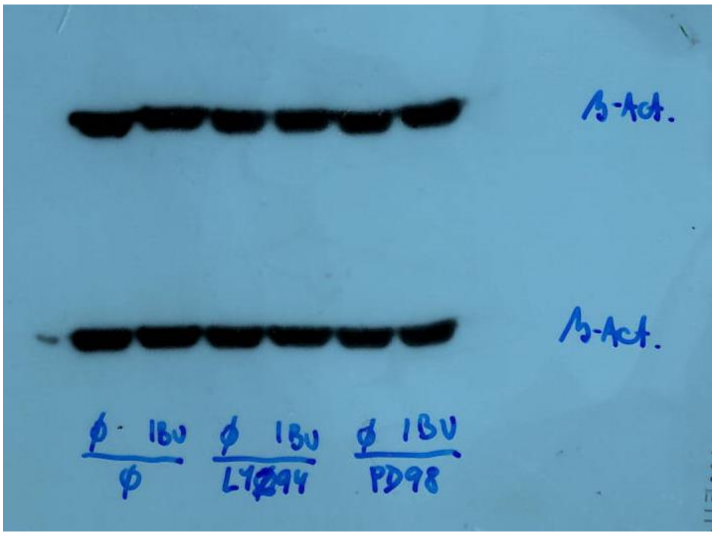

HCT116, RKO, FaDu – Lamin A (Figure 3B – USED IN FIGURE), p65 = NFkB p65 (Figure 3B), p50 = NFkB p50 (Figure

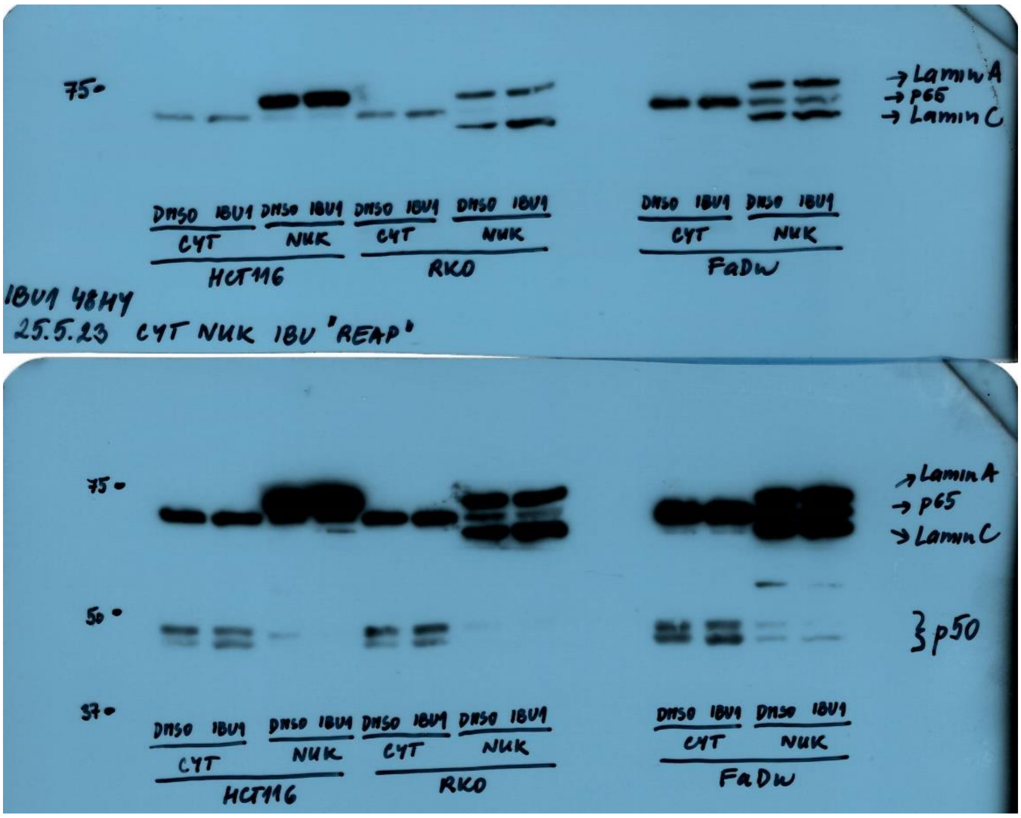

HCT116, RKO, FaDu – p50 = NFkB p50 (Figure 4B – USED IN FIGURE)

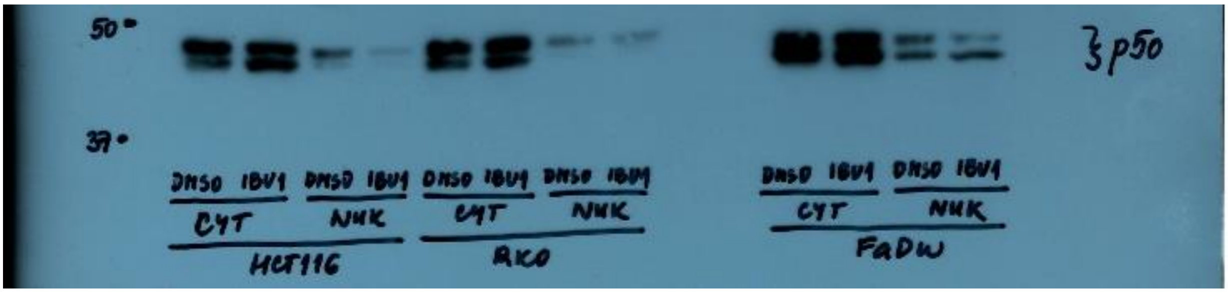

FaDu – p65 = NFkB p65 (Figure 3B – USED IN FIGURE)

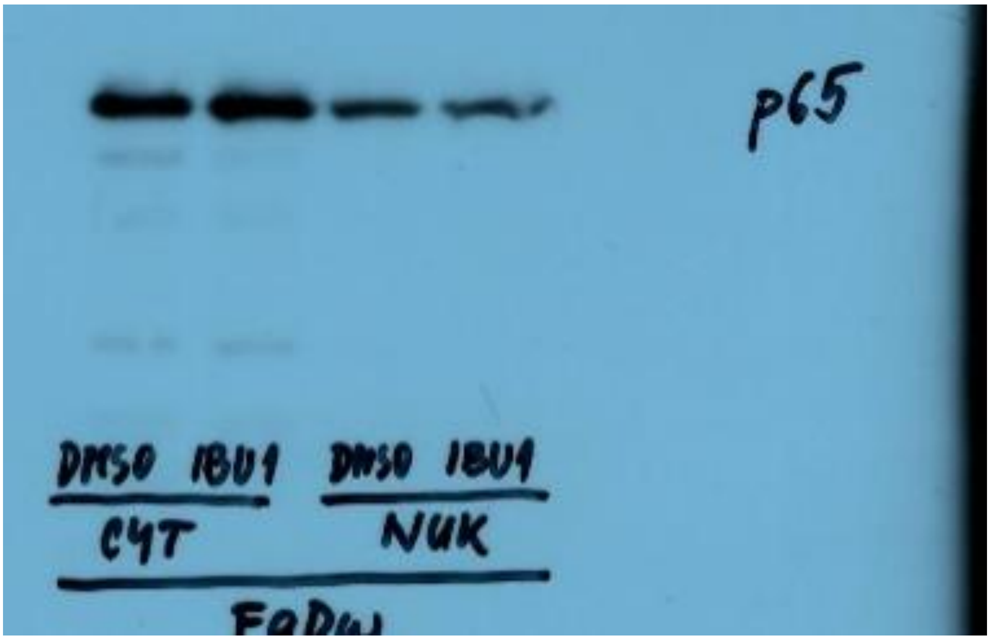

HCT116, RKO, FaDu, UM-22A – aktín = actin to different experiments/Western blots

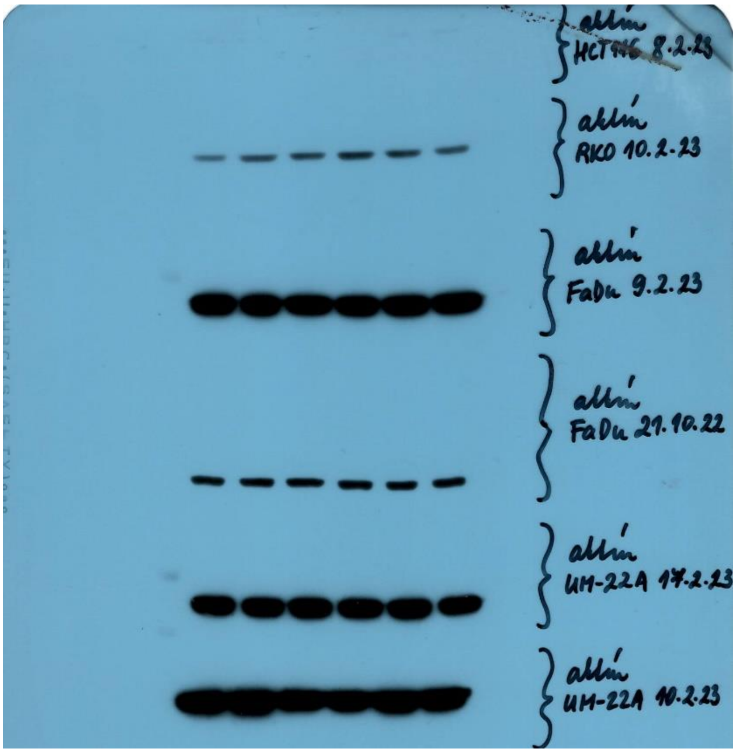

HCT116, RKO, UM-22A, FaDu – p65 (Figure 4A – USED IN FIGURE)

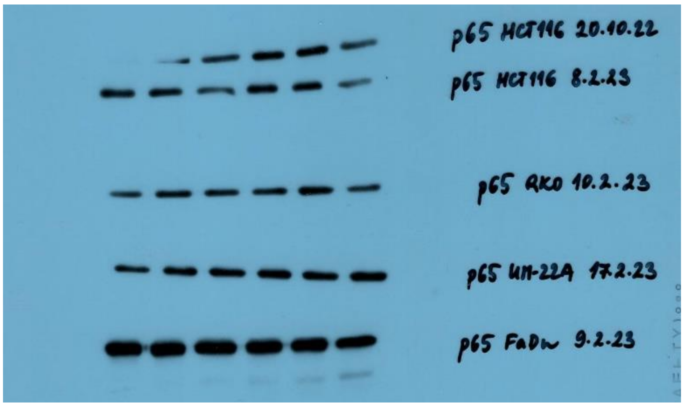

HCT116 – aktín = actin (Figure 4A – USED IN FIGURE)

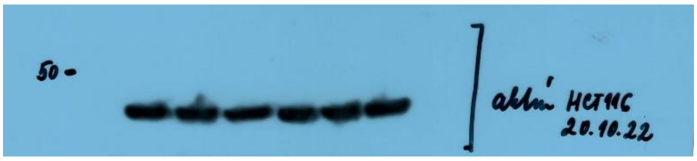

RKO – SURV = Survivin (Figure 4A – USED IN FIGURE)

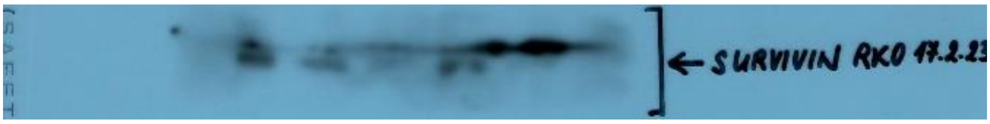

FaDu, UM-22A – p65 = NFkB p65 UM-22A (Figure 4B)

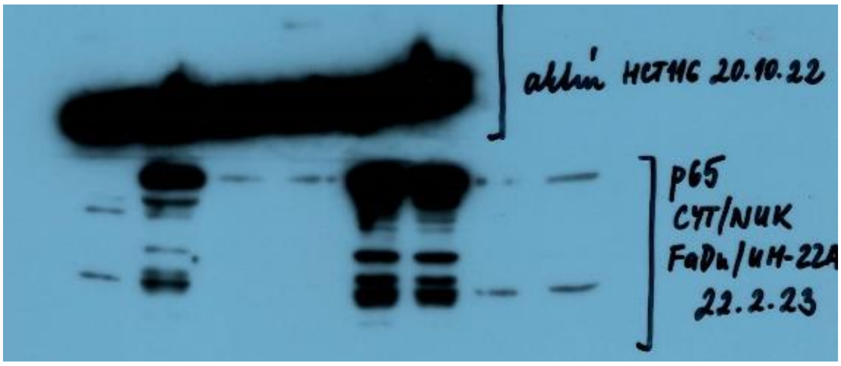

HCT116, UM-22A – HCT116 p-ERK ½ (Figure 5B – USED IN FIGURE), akín = actin = actins to different experiments/Western blots

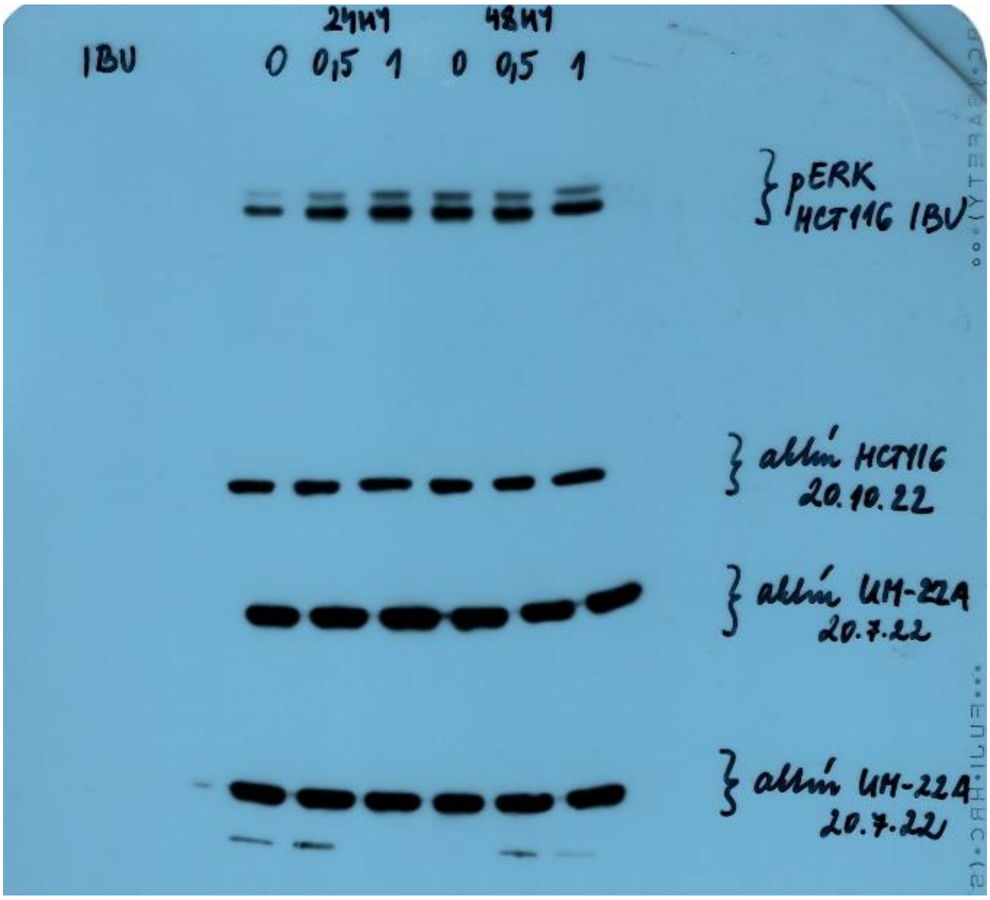

FaDu, UM-22A – p65 = NFkB p65 (Figure 4B – UM-22A USED IN FIGURE)

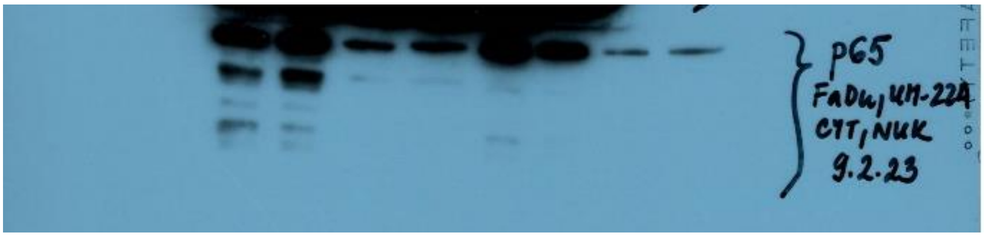

UM-22A – Lamin A (Figure 4B – USED IN FIGURE)

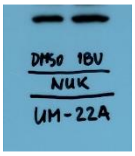

HCT116 sf = HCT116 3D, FaDu sf = FaDu 3D – cl PARP (Figure 5B), CA IX (Figure 1D), aktín = actin

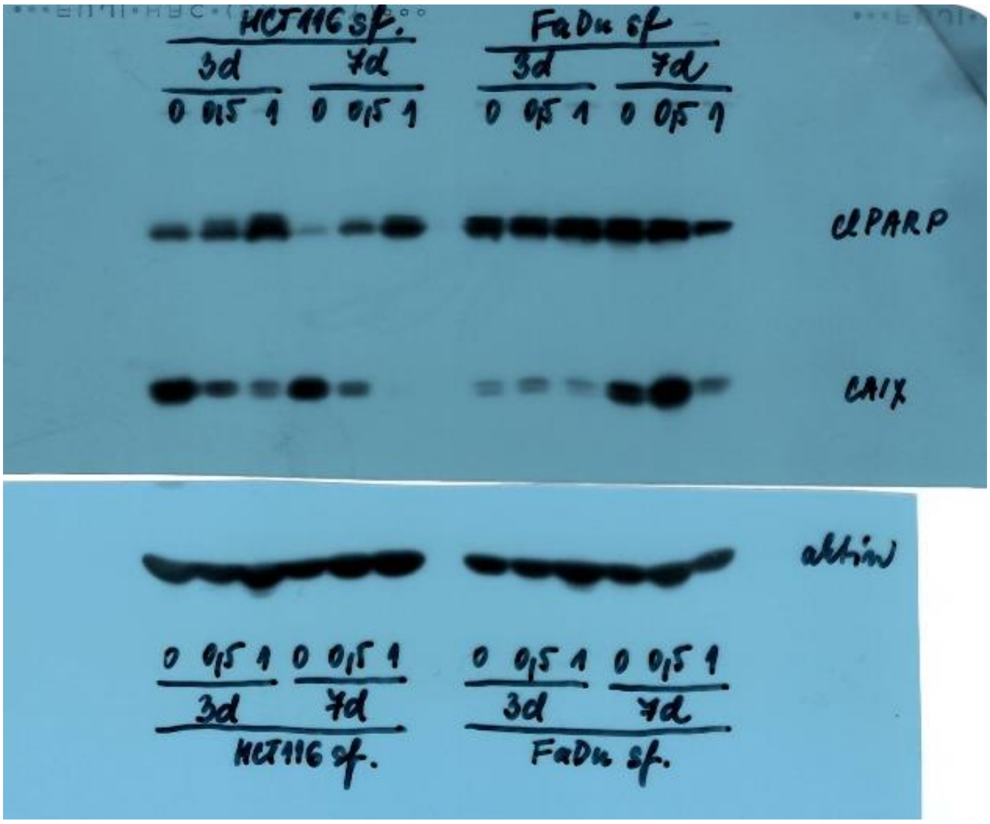

HCT116 – total ERK ½ (Figure 5B), LDHA (Figure 2D), COX2 (Figure 5B)

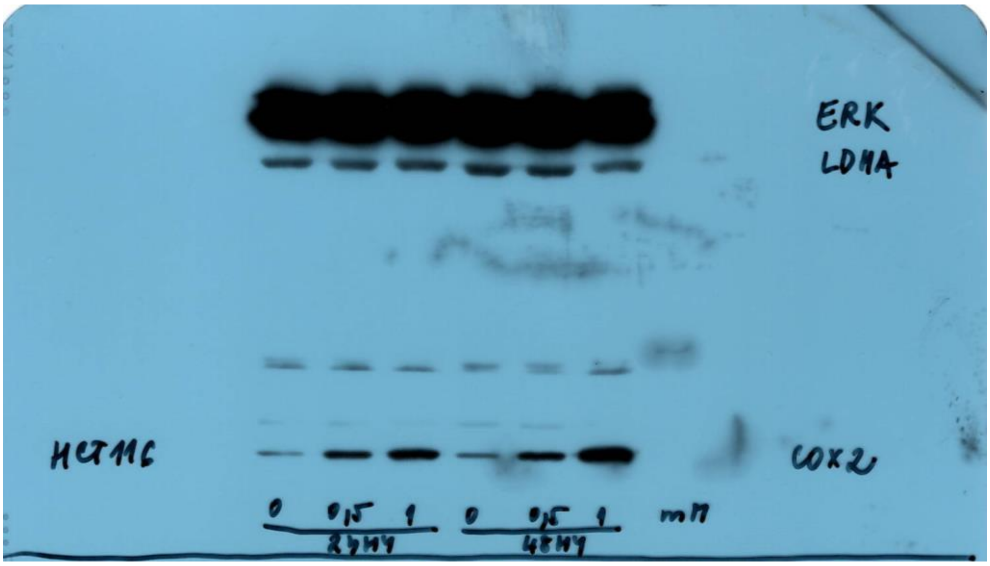

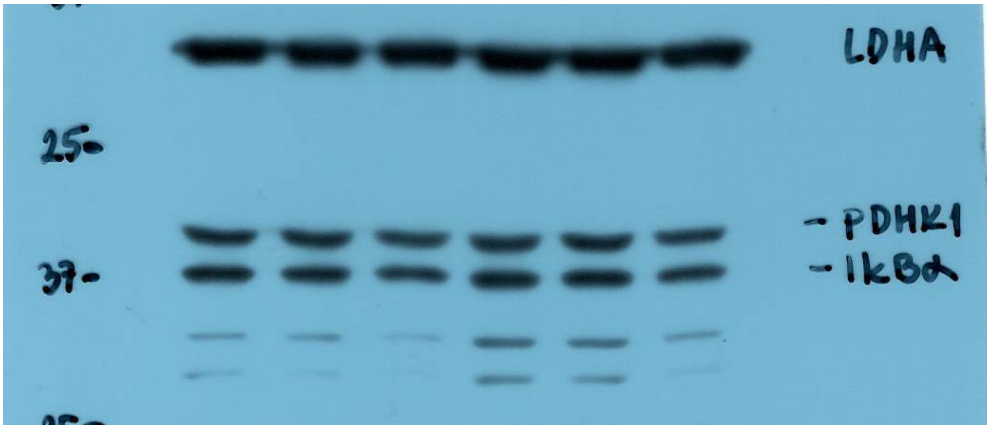

HCT116 - NFkB p105 (Figure 4A), pAKT (Figure 5B), pS9-GSK3beta (Figure 5B), Survivin (Figure 4A), HIF-1a (Figure 2C), p50 = NFkB p50 (Figure 4A), IkBa (Figure 4A), LC3B (Figure 6A)

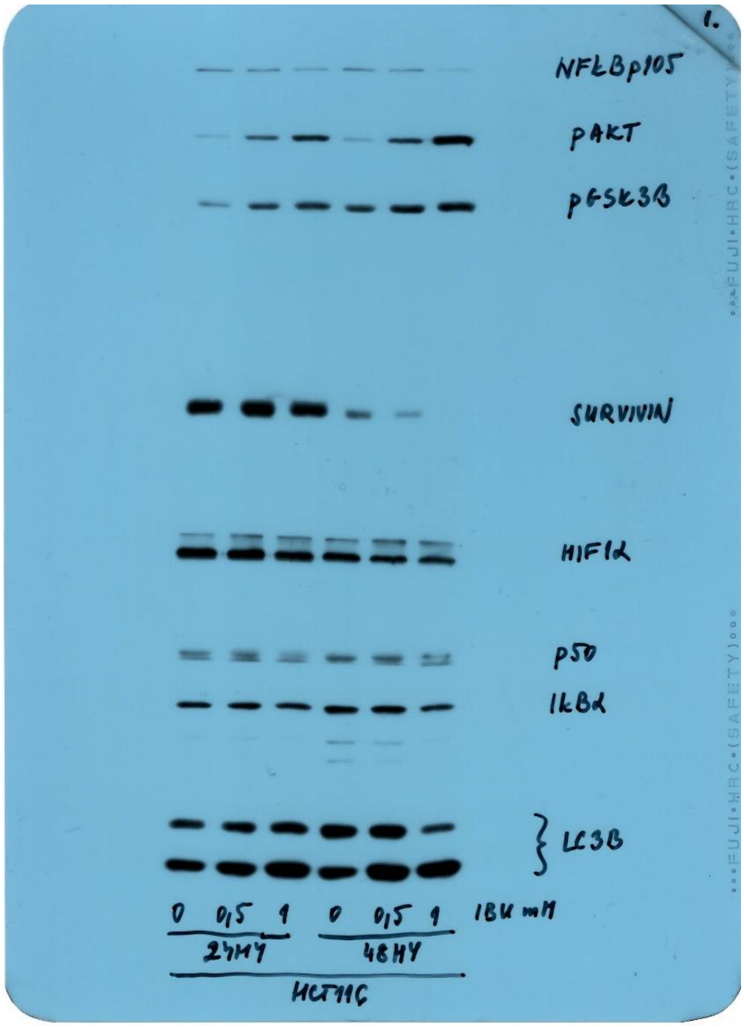

HCT116 – actin to HCT116 WB above

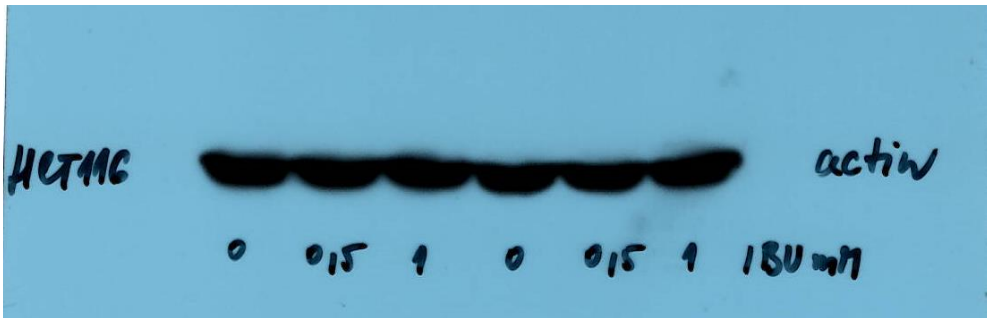

FaDu – total ERK ½ (Figure 5B), pSer9-GSK3beta (Figure 5B), LDHA (Figure 2D), PDHK1 (Figure 2D), Survivin (Figure 4A), COX2 (Figure 5B)

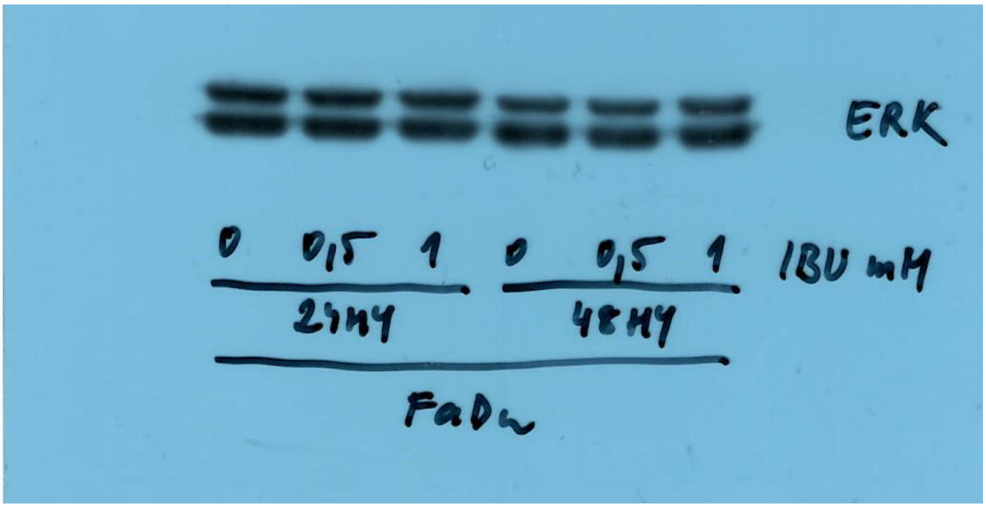

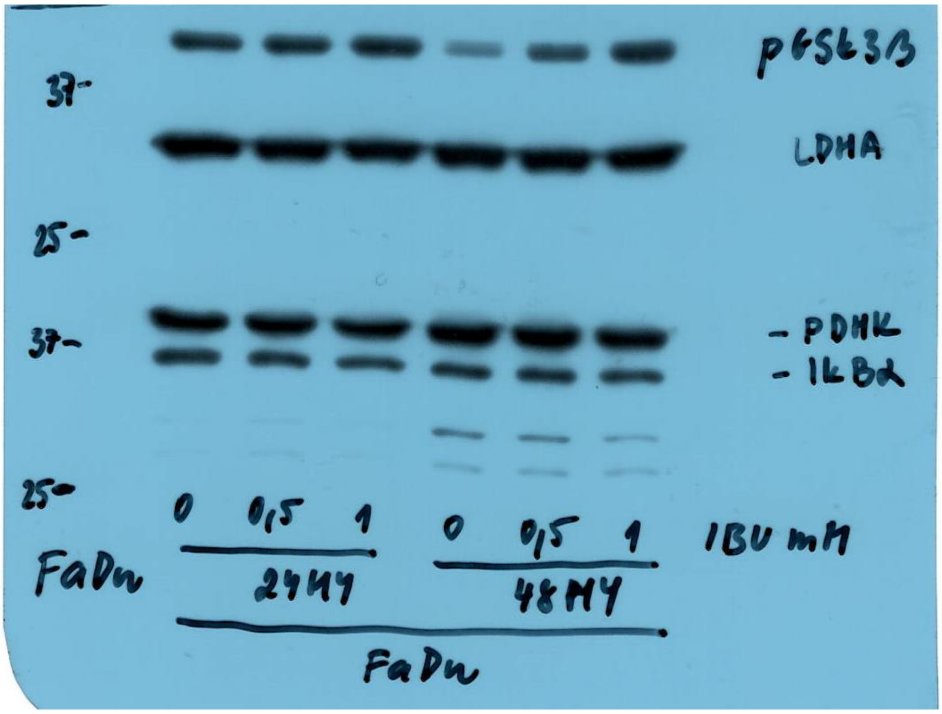

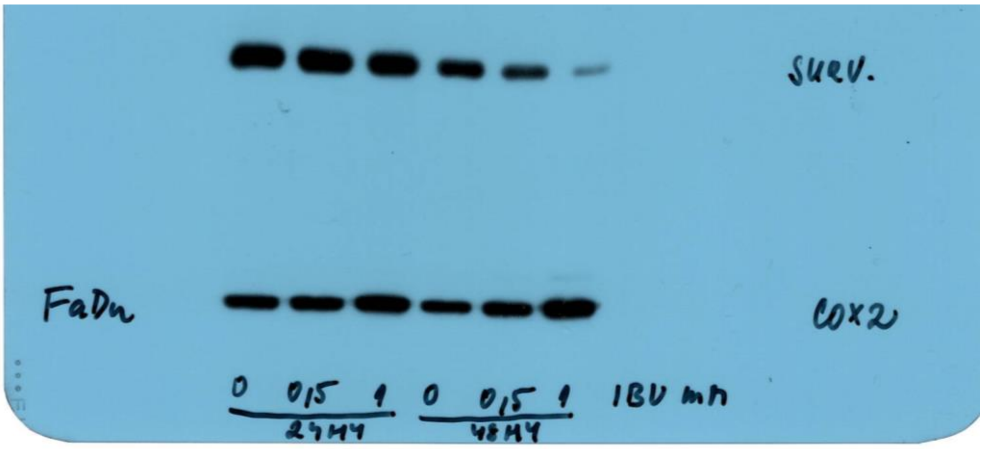

FaDu – NFkB p105 (Figure 4A), pAKT (Figure 5B), pSer9-GSK3beta (Figure 5B), HIF-1alpha (Figure 2C), NFkB p50 (Figure 4A), IkBa (Figure 4A), LC3B (Figure 6A)

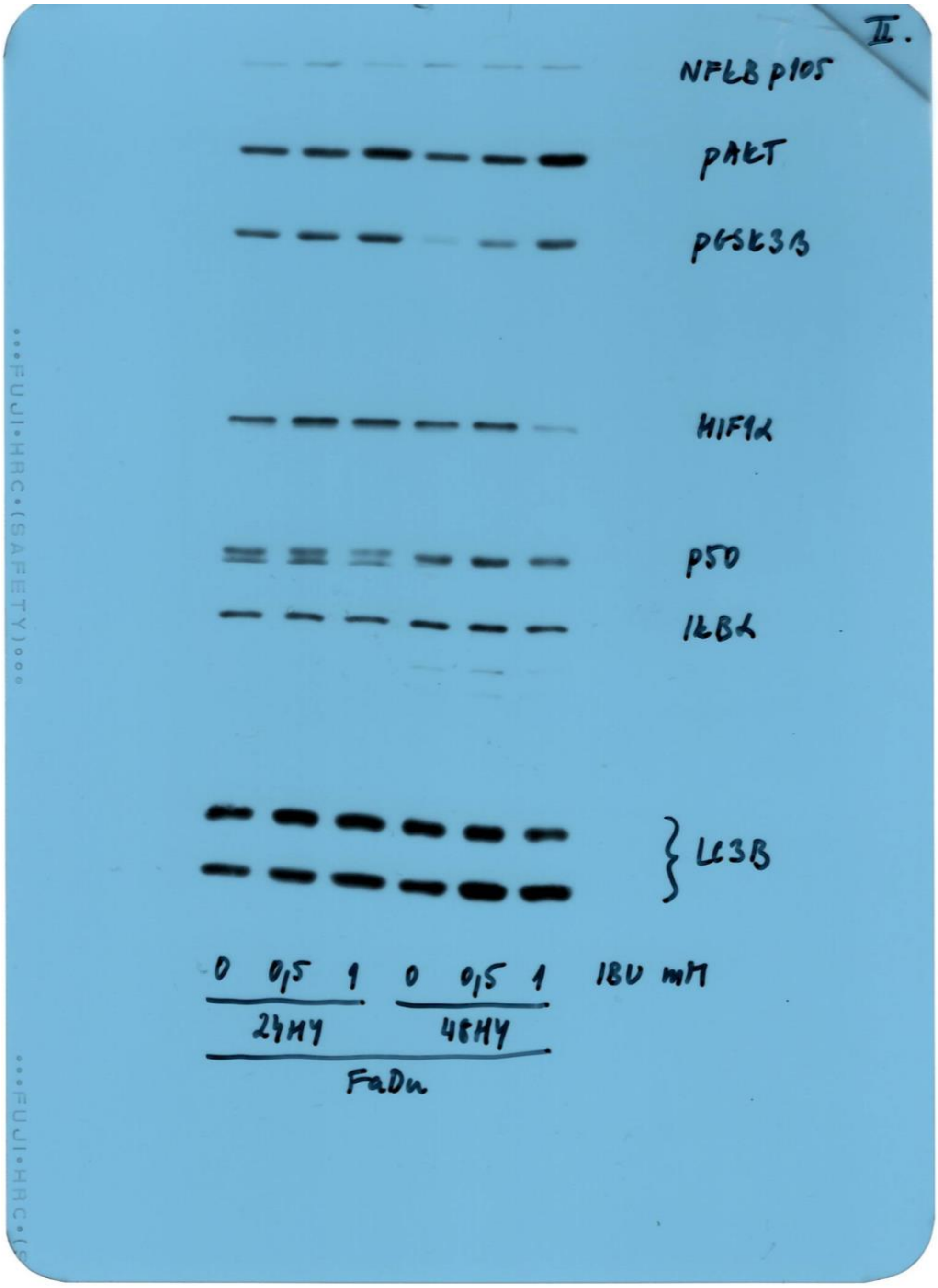

FaDu – actin to FaDu WB above

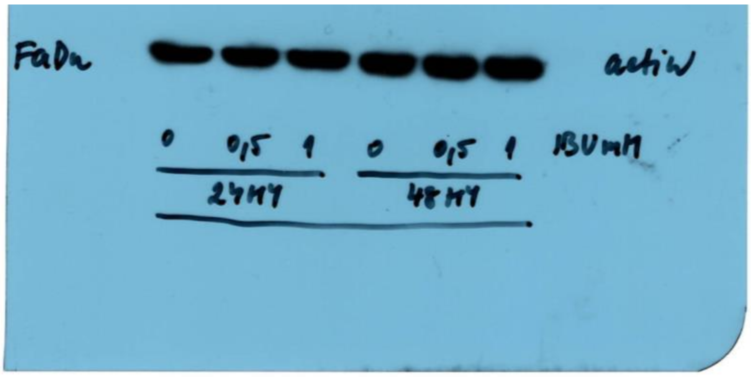

Supplement: S1 File — (PDF) [file pone.0323635.s001.pdf]
